# Biallelic Mutations in *ATP5F1D*, which Encodes a Subunit of ATP Synthase, Cause a Metabolic Disorder

**DOI:** 10.1016/j.ajhg.2018.01.020

**Published:** 2018-02-22

**Authors:** Monika Oláhová, Wan Hee Yoon, Kyle Thompson, Sharayu Jangam, Liliana Fernandez, Jean M. Davidson, Jennifer E. Kyle, Megan E. Grove, Dianna G. Fisk, Jennefer N. Kohler, Matthew Holmes, Annika M. Dries, Yong Huang, Chunli Zhao, Kévin Contrepois, Zachary Zappala, Laure Frésard, Daryl Waggott, Erika M. Zink, Young-Mo Kim, Heino M. Heyman, Kelly G. Stratton, Bobbie-Jo M. Webb-Robertson, Undiagnosed Diseases Network, David R. Adams, Mercedes E. Alejandro, Patrick Allard, Mahshid S. Azamian, Carlos A. Bacino, Ashok Balasubramanyam, Hayk Barseghyan, Gabriel F. Batzli, Alan H. Beggs, Babak Behnam, Anna Bican, David P. Bick, Camille L. Birch, Devon Bonner, Braden E. Boone, Bret L. Bostwick, Lauren C. Briere, Donna M. Brown, Matthew Brush, Elizabeth A. Burke, Lindsay C. Burrage, Shan Chen, Gary D. Clark, Terra R. Coakley, Joy D. Cogan, Cynthia M. Cooper, Heidi Cope, William J. Craigen, Precilla D’Souza, Mariska Davids, Jyoti G. Dayal, Esteban C. Dell’Angelica, Shweta U. Dhar, Ani Dillon, Katrina M. Dipple, Laurel A. Donnell-Fink, Naghmeh Dorrani, Daniel C. Dorset, Emilie D. Douine, David D. Draper, David J. Eckstein, Lisa T. Emrick, Christine M. Eng, Ascia Eskin, Cecilia Esteves, Tyra Estwick, Carlos Ferreira, Brent L. Fogel, Noah D. Friedman, William A. Gahl, Emily Glanton, Rena A. Godfrey, David B. Goldstein, Sarah E. Gould, Jean-Philippe F. Gourdine, Catherine A. Groden, Andrea L. Gropman, Melissa Haendel, Rizwan Hamid, Neil A. Hanchard, Lori H. Handley, Matthew R. Herzog, Ingrid A. Holm, Jason Hom, Ellen M. Howerton, Yong Huang, Howard J. Jacob, Mahim Jain, Yong-hui Jiang, Jean M. Johnston, Angela L. Jones, Isaac S. Kohane, Donna M. Krasnewich, Elizabeth L. Krieg, Joel B. Krier, Seema R. Lalani, C. Christopher Lau, Jozef Lazar, Brendan H. Lee, Hane Lee, Shawn E. Levy, Richard A. Lewis, Sharyn A. Lincoln, Allen Lipson, Sandra K. Loo, Joseph Loscalzo, Richard L. Maas, Ellen F. Macnamara, Calum A. MacRae, Valerie V. Maduro, Marta M. Majcherska, May Christine V. Malicdan, Laura A. Mamounas, Teri A. Manolio, Thomas C. Markello, Ronit Marom, Julian A. Martínez-Agosto, Shruti Marwaha, Thomas May, Allyn McConkie-Rosell, Colleen E. McCormack, Alexa T. McCray, Matthew Might, Paolo M. Moretti, Marie Morimoto, John J. Mulvihill, Jennifer L. Murphy, Donna M. Muzny, Michele E. Nehrebecky, Stan F. Nelson, J. Scott Newberry, John H. Newman, Sarah K. Nicholas, Donna Novacic, Jordan S. Orange, J. Carl Pallais, Christina G.S. Palmer, Jeanette C. Papp, Neil H. Parker, Loren D.M. Pena, John A. Phillips, Jennifer E. Posey, John H. Postlethwait, Lorraine Potocki, Barbara N. Pusey, Chloe M. Reuter, Amy K. Robertson, Lance H. Rodan, Jill A. Rosenfeld, Jacinda B. Sampson, Susan L. Samson, Kelly Schoch, Molly C. Schroeder, Daryl A. Scott, Prashant Sharma, Vandana Shashi, Edwin K. Silverman, Janet S. Sinsheimer, Kevin S. Smith, Rebecca C. Spillmann, Kimberly Splinter, Joan M. Stoler, Nicholas Stong, Jennifer A. Sullivan, David A. Sweetser, Cynthia J. Tifft, Camilo Toro, Alyssa A. Tran, Tiina K. Urv, Zaheer M. Valivullah, Eric Vilain, Tiphanie P. Vogel, Colleen E. Wahl, Nicole M. Walley, Chris A. Walsh, Patricia A. Ward, Katrina M. Waters, Monte Westerfield, Anastasia L. Wise, Lynne A. Wolfe, Elizabeth A. Worthey, Shinya Yamamoto, Yaping Yang, Guoyun Yu, Diane B. Zastrow, Allison Zheng, Michael Snyder, Jason D. Merker, Stephen B. Montgomery, Paul G. Fisher, René G. Feichtinger, Johannes A. Mayr, Julie Hall, Ines A. Barbosa, Michael A. Simpson, Charu Deshpande, Katrina M. Waters, David M. Koeller, Thomas O. Metz, Andrew A. Morris, Susan Schelley, Tina Cowan, Marisa W. Friederich, Robert McFarland, Johan L.K. Van Hove, Gregory M. Enns, Shinya Yamamoto, Euan A. Ashley, Michael F. Wangler, Robert W. Taylor, Hugo J. Bellen, Jonathan A. Bernstein, Matthew T. Wheeler

**Affiliations:** 1 Wellcome Centre for Mitochondrial Research, Institute of Neuroscience, Newcastle University, Newcastle upon Tyne NE2 4HH, UK; 2 Howard Hughes Medical Institute, Baylor College of Medicine, Houston, TX 77030, USA; 3 Department of Molecular and Human Genetics, Baylor College of Medicine, Houston, TX 77030, USA; 4 Center for Undiagnosed Diseases, Stanford University, Stanford, CA 94305, USA; 5 Biological Sciences Division, Earth and Biological Sciences Directorate, Pacific Northwest National Laboratory, Richland, WA 99352, USA; 6 Clinical Genomics Program, Stanford Health Care, Stanford, CA 94305, USA; 7 Department of Genetics, Stanford University School of Medicine, Stanford, CA 94305, USA; 8 Department of Pathology, Stanford University, Stanford, CA 94305, USA; 9 Computing & Analytics Division, National Security Directorate, Pacific Northwest National Laboratory, Richland, WA 99352, USA; 10 Department of Pediatrics, Paracelsus Medical University, 5020 Salzburg, Austria; 11 Department of Neuroradiology, Royal Victoria Infirmary, Newcastle upon Tyne NE1 4LP, UK; 12 Department of Medical and Molecular Genetics, King’s College London School of Basic and Medical Biosciences, London SE1 9RT, UK; 13 Clinical Genetics Unit, Guys and St. Thomas’ NHS Foundation Trust, London SE1 9RT, UK; 14 Department of Molecular & Medical Genetics, Oregon Health & Science University, Portland, OR 97239, USA; 15 Institute of Human Development, University of Manchester, Manchester M13 9PL, UK; 16 Willink Metabolic Unit, Genomic Medicine, Saint Mary’s Hospital, Manchester University NHS Foundation Trust, Manchester M13 9WL, UK; 17 Department of Pediatrics, Stanford University School of Medicine, Stanford, CA 94305, USA; 18 Clinical Genetics and Metabolism, Department of Pediatrics, University of Colorado at Denver, Aurora, CO 80045, USA; 19 Program in Developmental Biology, Baylor College of Medicine, Houston, TX 77030, USA; 20 Jan and Duncan Neurological Research Institute, Texas Children’s Hospital, Houston, TX 77030, USA; 21 Department of Neuroscience, Baylor College of Medicine, Houston, TX 77030, USA; 22 Department of Medicine, Stanford University School of Medicine, Stanford, CA 94305, USA

**Keywords:** mitochondrial disease, complex V, ATP synthase, exome sequencing, oxidative phosphorylation, lactic acidosis, hyperammonemia, 3-methylglutaric aciduria, model organism, fibroblast

## Abstract

ATP synthase, H^+^ transporting, mitochondrial F1 complex, δ subunit (ATP5F1D; formerly ATP5D) is a subunit of mitochondrial ATP synthase and plays an important role in coupling proton translocation and ATP production. Here, we describe two individuals, each with homozygous missense variants in *ATP5F1D*, who presented with episodic lethargy, metabolic acidosis, 3-methylglutaconic aciduria, and hyperammonemia. Subject 1, homozygous for c.245C>T (p.Pro82Leu), presented with recurrent metabolic decompensation starting in the neonatal period, and subject 2, homozygous for c.317T>G (p.Val106Gly), presented with acute encephalopathy in childhood. Cultured skin fibroblasts from these individuals exhibited impaired assembly of F_1_F_O_ ATP synthase and subsequent reduced complex V activity. Cells from subject 1 also exhibited a significant decrease in mitochondrial cristae. Knockdown of *Drosophila ATPsynδ*, the *ATP5F1D* homolog, in developing eyes and brains caused a near complete loss of the fly head, a phenotype that was fully rescued by wild-type human *ATP5F1D.* In contrast, expression of the *ATP5F1D* c.245C>T and c.317T>G variants rescued the head-size phenotype but recapitulated the eye and antennae defects seen in other genetic models of mitochondrial oxidative phosphorylation deficiency. Our data establish c.245C>T (p.Pro82Leu) and c.317T>G (p.Val106Gly) in *ATP5F1D* as pathogenic variants leading to a Mendelian mitochondrial disease featuring episodic metabolic decompensation.

## Main Text

Mitochondrial diseases are clinically and genetically heterogeneous. Findings such as hyperammonemia, lactic acidosis, and rhabdomyolysis suggest mitochondrial dysfunction and can occur as a result of defects in fatty acid oxidation as well as disorders of the respiratory chain. Defects in the electron transport chain (ETC), which underlies oxidative phosphorylation (OXPHOS), can be caused by mutations in the nuclear or mitochondrial genome.^1^, ^2^ Accordingly, inheritance can be autosomal, sex linked, or maternal. Presentations vary widely and range from lethal neonatal metabolic decompensation to chronic progressive disorders of adulthood.

Complex V is the final multi-subunit complex of the OXPHOS system. It harnesses energy from the proton electrochemical gradient to synthesize ATP from ADP^3^ and inorganic phosphate, which is the main source of energy for intracellular metabolic pathways.^4^ Mitochondrial ATP synthase consists of two main functional domains, the soluble F_1_ catalytic portion in the mitochondrial matrix and the inner-membrane-embedded F_O_, which allows protons to pass from the intermembrane space to the matrix (reviewed by Jonckheere et al.^5^). Two subunits of the F_O_ (a and A6L) are encoded by mtDNA (*MT-ATP6* and *MT-ATP8*), whereas the other subunits and accessory factors are encoded by the nuclear genome. Although mitochondrial disorders due to defects in mitochondrial complex V have been reported, they are very rare in comparison with those due to mutations in the genes encoding the proteins of the other complexes (I–IV).^6^, ^7^

We report the clinical and genetic findings of two children with suspected mitochondrial disease from unrelated families. Subject 1 is the only child of first-cousin Mexican-American parents. On the second day of life, she presented with lethargy and severe anion-gap acidosis. Initial laboratory investigations showed hypoglycemia (28 mg/dL [normal 45–100]), lactic acidosis (34 mmol/L [normal < 2.1]), and hyperammonemia (359 μmol/L [normal < 30]). Initial management included intravenous fluids with dextrose and intravenous lipid administration. Within 24 hr, lactic acid and ammonia had decreased to 4.8 mmol/L and 70 μmol/L, respectively. Ammonia-scavenging medications were not administered. Qualitative organic acid studies showed moderate to marked elevation of lactic, fumaric, malic, p-hydroxyphenyllactic, and 3-methylglutaconic acids. An acylcarnitine profile showed nonspecific elevations of numerous short-, medium-, and long-chain acylcarnitine species. Creatine kinase was not assessed during her initial presentation. Brain MRI with magnetic resonance spectroscopy was normal. Her most recent evaluation was at 9 years of age. 3-methylglutaconic aciduria has been a persistent finding in urine organic acid analysis. She has mild developmental delays and short stature. Between the ages of 1 and 4 years, she was noted to have dilated cardiomyopathy and subsequent normalization of resting systolic function. Ophthalmologic examination at 8 years of age showed a prominent macular reflex. No other findings were noted. Neurologic examination at 9 years of age showed mild proximal weakness (4/5) greater than distal weakness (5/5) in her extremities. She additionally had gait imbalance and ankle contractures with reduced reflexes (1+). Cranial nerve examination showed slightly decreased strength with eye closure. Cerebellar examination and sensation were normal. She has had at least nine episodes of metabolic decompensation manifesting with lactic acidosis and muscle breakdown, which required hospital admission. During decompensation, serum creatine kinase has been repeatedly elevated to greater than 500 U/L and as high as 1,109 U/L. These episodes have been responsive to intravenous fluids with dextrose. Severe hyperammonemia has not recurred since the newborn period. She has been treated with oral supplements including alpha-lipoic acid, ubiquinone, riboflavin, thiamine, biotin, pantothenic acid, and ascorbic acid and has experienced subjective improvement in her physical stamina.

Subject 2 is the first child born to healthy first-cousin UK Asian parents, and he has a healthy younger brother. He was born at term by vacuum-assisted delivery after an uneventful pregnancy. There were no perinatal problems. His speech was delayed, and he received speech therapy, but he otherwise met typical developmental milestones. At age 4 years and 10 months, he presented with an encephalopathic illness after 24 hr of coryza and fever. He was witnessed to have a progressive deterioration in the level of consciousness over several hours and had a brief tonic-clonic seizure, which was managed with phenobarbital. Ultimately, he required intubation and mechanical ventilation, which was maintained for 2 days. He had ketoacidosis and hyperammonemia (maximum 262 μmol/L [normal < 30]). Plasma lactate was 5.3 mmol/L (normal < 2.1) at presentation but decreased to 2.1 mmol/L within 5 hr and subsequently to 1.1 mmol/L, at which stage the cerebrospinal fluid lactate was 1.8 mmol/L (normal < 2.5). Initial treatment included intravenous fluids with dextrose, intravenous carnitine (100 mg/kg/day), and sodium benzoate (250 mg/kg/day). The ammonia level normalized within 24 hr. Neuroimaging showed diffuse swelling of the cerebral cortex bilaterally, especially in the temporal lobes, as well as lesser changes in the cerebellar hemispheres (Figure S1). There was swelling and signal change in the subcortical and deep white matter, although the periventricular white matter was spared. There were also signal changes in the thalami, midbrain, pons, corpus callosum, and basal ganglia. MRI 1 year later showed resolution of these abnormal findings. The transient nature of the MRI findings was interpreted as evidence that they might have reflected the presence of edema that resolved over time. After this episode, he made a full recovery to his prior baseline. He attends a regular school, and at 6 years of age he had a full-scale IQ of 81 (Wechsler Preschool and Primary Scale of Intelligence^8^) and poor attention (as assessed by a score of 51 [first percentile] on the Attention & Concentration Index of the Children’s Memory Scale^9^). He now has mildly impaired exercise tolerance, tires easily, and uses a wheelchair for long distances. Neurologic examination after his initial presentation noted mild hypotonia, but this has since resolved. He has pes planus, pes adductus, and dyspraxia of gait but no other abnormalities on detailed neurologic examination. The cranial nerve, motor, sensory, and cerebellar examinations have otherwise been normal. On recent routine evaluation, 12-lead electrocardiography and echocardiography were normal. Organic acid analysis has persistently shown a mild increase in 3-methylglutaconic and 3-methylglutaric acid excretion. He has been a fussy eater since infancy and receives much of his nutrition as liquid formula. He periodically develops lethargy and emesis typically in association with febrile illness. Symptoms are improved by oral dextrose containing fluids. He experiences emesis approximately twice a week and has frequent stomach aches. He has a history of intermittent squint and has developed amblyopia of the left eye, despite patching of the right eye. There are no other ophthalmological abnormalities. His linear growth has been typical for his age, and physical examination shows no significant findings. The parents and younger sibling (currently 4 years of age) are in good health.

Informed consent for diagnostic and research studies was obtained for both subjects in accordance with the Declaration of Helsinki protocols and approved by the central institutional review board (IRB) at the NIH National Human Genome Research Institute for the Undiagnosed Diseases Network (subject 1) and by the local IRB in Newcastle upon Tyne, UK (subject 2).

Initial diagnostic analyses of cultured skin fibroblasts for pyruvate carboxylase, pyruvate dehydrogenase, and enzyme activities of respiratory chain complexes I–IV in subject 1 were normal. Complex V was not assessed during these studies. Subsequent blue-native PAGE (BN-PAGE) with in-gel activity staining showed qualitatively decreased activity of complex V (Figure S2). For subject 2, complexes I–IV of the mitochondrial respiratory chain were all within normal ranges in muscle, as were routine histology and histochemistry. Pyruvate dehydrogenase activity was normal in cultured skin fibroblasts. Subsequent analysis of the activity of respiratory chain complexes in fibroblasts from each affected individual showed a marked decrease in complex V enzymatic activity (Table 1).

**Table 1:** Genetic, Biochemical, and Clinical Findings in Individuals with Biallelic *ATP5F1D* Variants

**ID**	**Sex**	***ATP5F1D* Variants**		**OXPHOS Activities in Cultured Skin Fibroblasts**					**Clinical Presentation**	
		**cDNA (GenBank:****NM_001687.4****)**	**Protein (GenBank:****NP_001687.1****)**	**Respiratory Chain Complex**	**Mean Enzyme Activity (%)**	**Absolute Values**	**Normal Range of Activities**	**Muscle Biopsy**	**Age at Presentation**	**Salient Clinical Features**
S1	female	c.[245C>T];[245C>T]	p.[Pro82Leu];[Pro82Leu]	I	83%	24	18–53	normal histology and respiratory chain enzymes	2 days	hyperammonemia, cardiomyopathy, lactic acidosis, rhabdomyolysis fatigability, short stature
				I + III	267%	310	61–220			
				II	92%	71	54–124			
				II + III	130%	180	79–219			
				IV	44%	162	270–659			
				V	5% (↓↓↓)	7	78–287			
				CS	63%	197	225–459			
S2	male	c.[317T>G];[317T>G]	p.[Val106Gly];[Val106Gly]	I	93%	27	18–53	normal histology and respiratory chain enzymes	4 years and 10 months	hyperammonemia, ketoacidosis, delayed speech
				I + III	151%	174	61–220			
				II	98%	76	54–124			
				II + III	139%	193	79–219			
				IV	142%	519	270–659			
				V	16% (↓↓)	23	78–287			
				CS	101%	314	225–459			

Analysis of mtDNA from blood in both affected individuals showed no mtDNA rearrangements or point mutations, and the mtDNA copy number was normal. Whole-exome sequencing (WES) was performed according to previously described methodologies and filtering pipelines.^10^, ^11^, ^12^, ^13^ In subject 1, exome sequencing was performed with VCRome 2.1 in-solution exome probes, as well as additional probes for over 2,600 Mendelian-disease-related genes. Library DNA was sequenced on an Illumina HiSeq for 100 bp paired-end reads. Data analysis was performed with Mercury 1.0 and was followed by reanalysis using phenotype- and inheritance-model-based filters with Ingenuity Variant Analysis (QIAGEN) and a curated list of mitochondrial expressed genes. Variants were confirmed by Sanger sequencing of DNA samples from the affected subject and parents. In subject 2, exome sequencing was performed in the family trio with Agilent SureSelectXT All Exon V5 on a HiSeq 2500 with 100 bp paired-end reads. Variant calls were generated with an in-house pipeline as previously described with minor alterations.^10^ Variant files were annotated with respect to genes and variant functional consequences with the ANNOVAR tool. Further annotation included information on variant novelty and estimated population frequencies from cross-referencing identified variants with publicly available data and >1,000 control exomes processed with a Novoalign-based pipeline.

**Figure 1:**
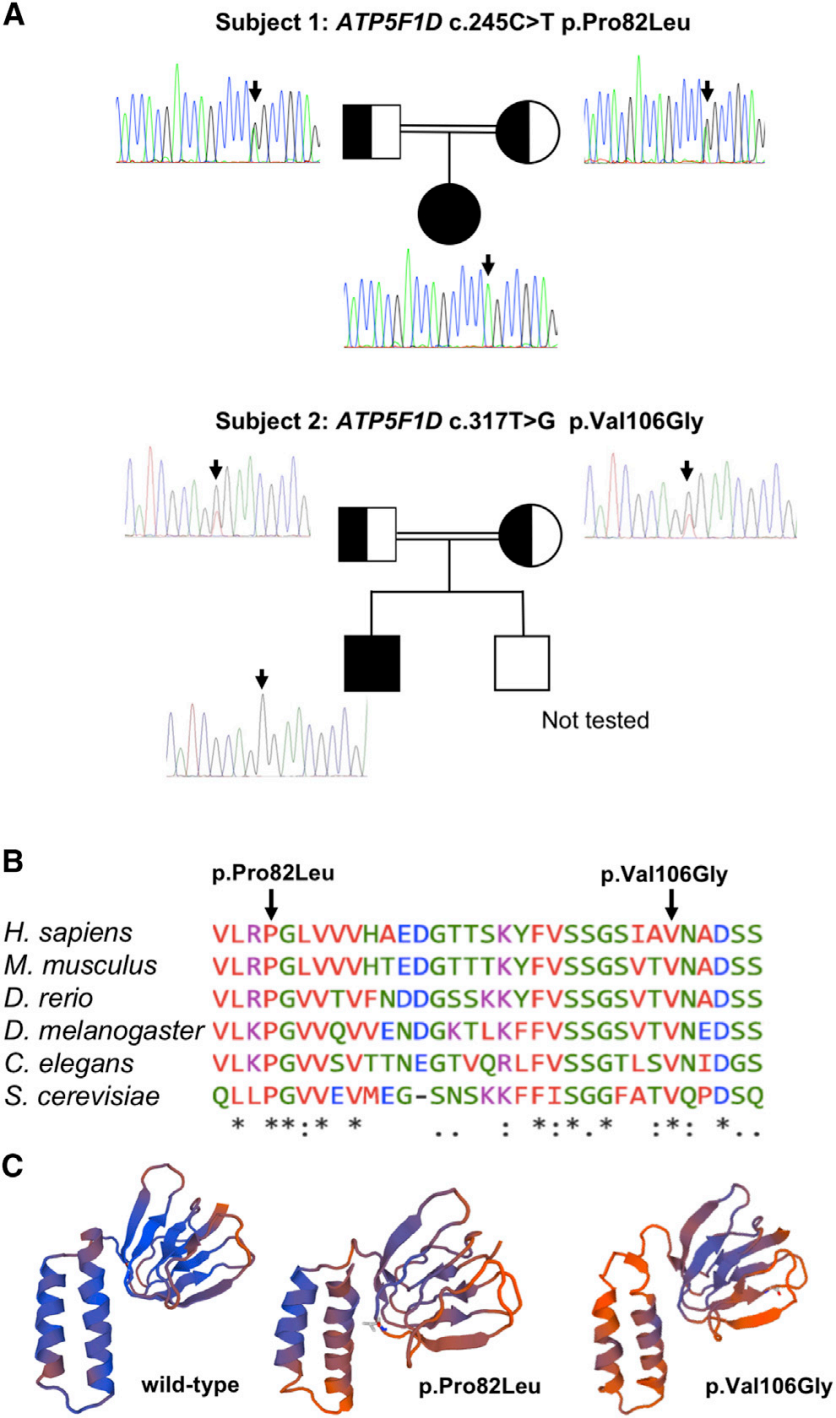
Molecular Genetic Studies of *ATP5F1D* Variants (A) Pedigrees and sequencing chromatograms of the two affected families show segregation of the homozygous *ATP5F1D* variant c.245C>T (p.Pro82Leu) in subject 1 and c.317T>G (p.Val106Gly) in subject 2. (B) Multiple-sequence alignment confirms evolutionary conservation of p.Pro82Leu and p.Val106Gly in both human and flies. (C) SWISS-MODEL-predicted structure of wild-type, p.Pro82Leu, and p.Val106Gly ATP5F1D.^15^

In both subjects, WES identified biallelic variants in *ATP5F1D* (formerly *ATP5D* [MIM: 603150; GenBank: NM_001687.4]), which encodes the F_1_ δ subunit of complex V.^14^ *ATP5F1D* is located at 19p13.3 (1,241,750–1,244,825 [GRCh38.p7]). The predominant transcript consists of four exons encoding a 146 amino acid mature protein with a 22 amino acid presequence.^14^ Research reanalysis of proband-only clinical WES data from subject 1 identified a homozygous c.245C>T (p.Pro82Leu) variant in *ATP5F1D*. Sanger sequencing confirmed bi-parental inheritance of the c.245C>T variant (Figure 1A). There was no detectable abnormality in the abundance or splicing of the *ATP5F1D* transcript (Figure S3). In parallel, WES was undertaken in the family trio of subject 2, revealing a homozygous c.317T>G (p.Val106Gly) variant in exon 3 of *ATP5F1D*. Analysis of WES and Sanger confirmation in the parents demonstrated bi-parental inheritance of the c.317T>G (p.Val106Gly) variant (Figure 1A). The identified variants (p.Pro82Leu and p.Val106Gly) affect highly conserved amino acids (Figure 1B). The c.245C>T variant has been observed in 1 of 142,292 total alleles (1 of 23,192 alleles of Latino ethnicity) in the gnomAD dataset and has not been seen in other publicly searchable datasets, whereas c.317T>G had not been observed in any dataset.^16^ *In silico* structural modeling indicated that each amino acid variant induces a change in the predicted protein structure (Figure 1C).^15^

Although the two subjects both had features of mitochondrial disease and metabolic decompensation, they differed in that subject 1 presented a few days after birth, had elevated creatine kinase, and had normal brain MRI. Subject 2 was not evaluated for mitochondrial phenotypes until after 4 years of age. Because both had homozygous missense variants in *ATP5F1D* and because no disease annotation for *ATP5F1D* is known, we undertook additional studies in subject cells and in *Drosophila melanogaster* to determine whether these missense changes were pathogenic.

**Figure 2:**
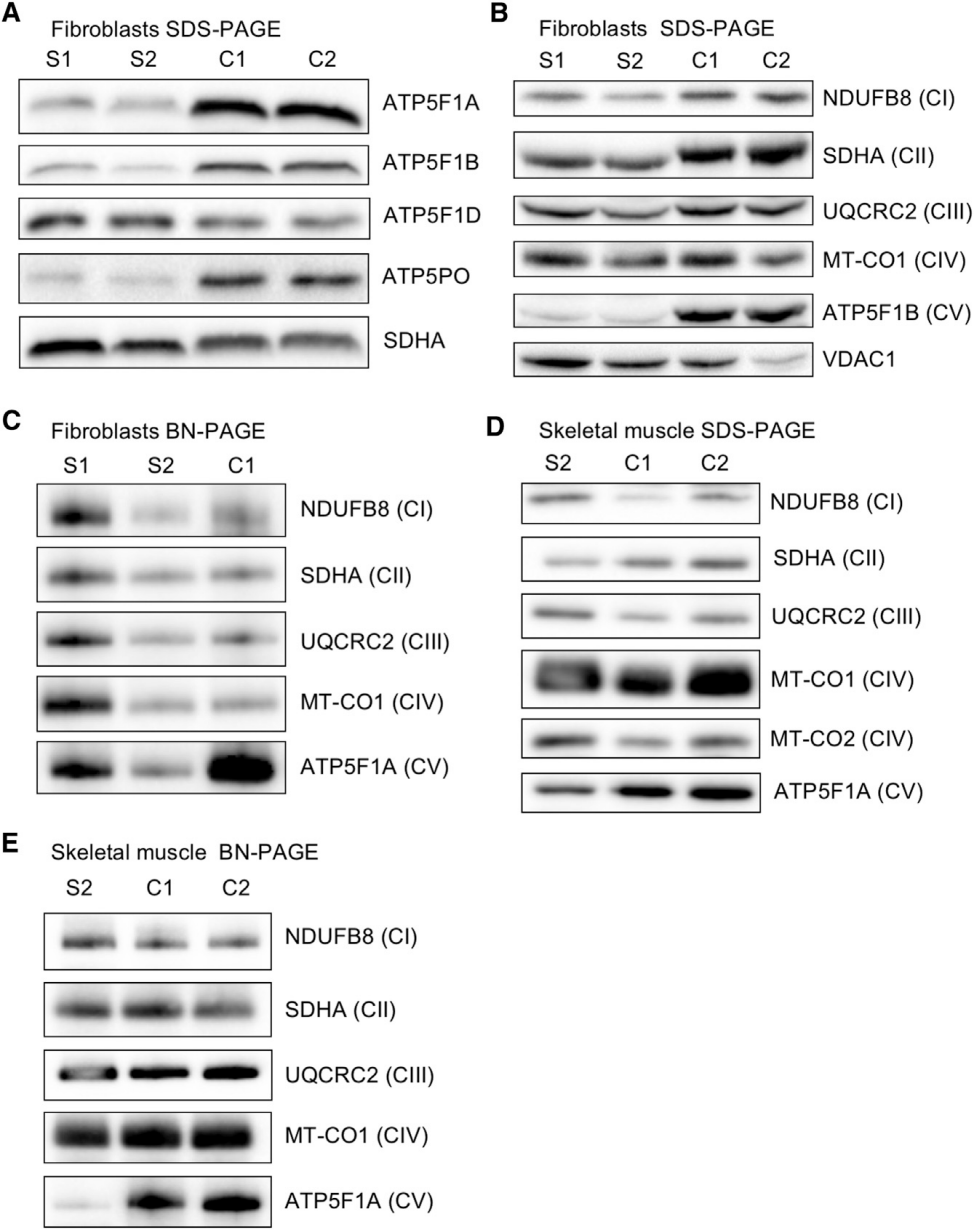
Biallelic Variants in *ATP5F1D* Impair the Steady-State Amounts of the F_1_F_O_ ATP Synthase Complex and Subunits Immunoblot and BN-PAGE analysis were carried out on subject cultured skin fibroblasts and skeletal muscle samples as previously described.^11^, ^17^, ^18^ SDS-PAGE and immunoblot analysis of whole-cell lysates (40 μg) isolated from cultured skin fibroblasts of affected subjects 1 (S1) and 2 (S2) and age-matched control individuals show (A) the steady-state amounts of complex V subunits (ATP5F1A, ATP5F1B, ATP5F1D, and ATP5PO) and (B) the amounts of individual OXPHOS complex subunits. One-dimensional BN-PAGE analysis was performed for assembled OXPHOS complexes in n-dodecyl-β-D-maltoside (DDM; 850520P, Sigma)-solubilized mitochondrial extracts isolated from control, S1, and S2 fibroblasts (C). Steady-state amounts (D) and assembly (E) of OXPHOS complexes and subunits in DDM-solubilized mitochondrial extracts from control and subject 2 skeletal muscle demonstrate a decrease in complex V. In (C) and (E), mitochondrial lysates (100 μg) were loaded on a 4%–16% native gel (Life Technologies), and then protein complexes were immobilized onto polyvinylidene difluoride membranes and subjected to immunoblotting with the indicated OXPHOS-subunit-specific antibodies. In (A)–(E), nuclear-encoded SDHA (ab14715, Abcam) or porin (VDAC1, ab14734, Abcam) was used as a loading control. Abbreviations are as follows: BN, blue native; CI, complex I; CII, complex II; CIII, complex III; CIV, complex IV; and CV, complex V.

To investigate the functional effects of the identified *ATP5F1D* variants, we performed OXPHOS protein analysis from cultured skin fibroblasts of each affected individual. Immunoblotting of protein extracts from subject fibroblasts showed that steady-state amounts of ATP5F1D were not affected (Figure 2A). However, other complex V subunits (ATP5F1A, ATP5F1B, and ATP5PO) were clearly decreased in abundance (Figure 2A). Double immunofluorescence staining of fibroblasts from subjects 1 and 2 (Figure S4) revealed lower signal of the complex V subunit ATP5F1A than of that in age-matched control cells, confirming abnormality of complex V. The abundance of other OXPHOS complex subunits was not decreased, whereas complex V subunits showed a marked reduction (Figure 2B). This was confirmed by BN-PAGE analysis, which showed a loss of complex V assembly, whereas other complexes were relatively unaffected (Figure 2C). We confirmed these findings in skeletal muscle extracts from subject 2, given that steady-state amounts of CI–CIV subunits and complexes were not affected, whereas the amounts of complex V subunit ATP5F1A (Figure 2D) and fully assembled complex V (Figure 2E) were markedly decreased. These data show that cells from the subjects exhibited reduced amounts of complex V. We posit that the missense changes present in both subjects do not alter the amount of ATP5F1D but instead lead to an inability of ATP5F1D to bind other F_1_ subunits correctly and thus result in reduced assembly of complex V.

**Figure 3:**
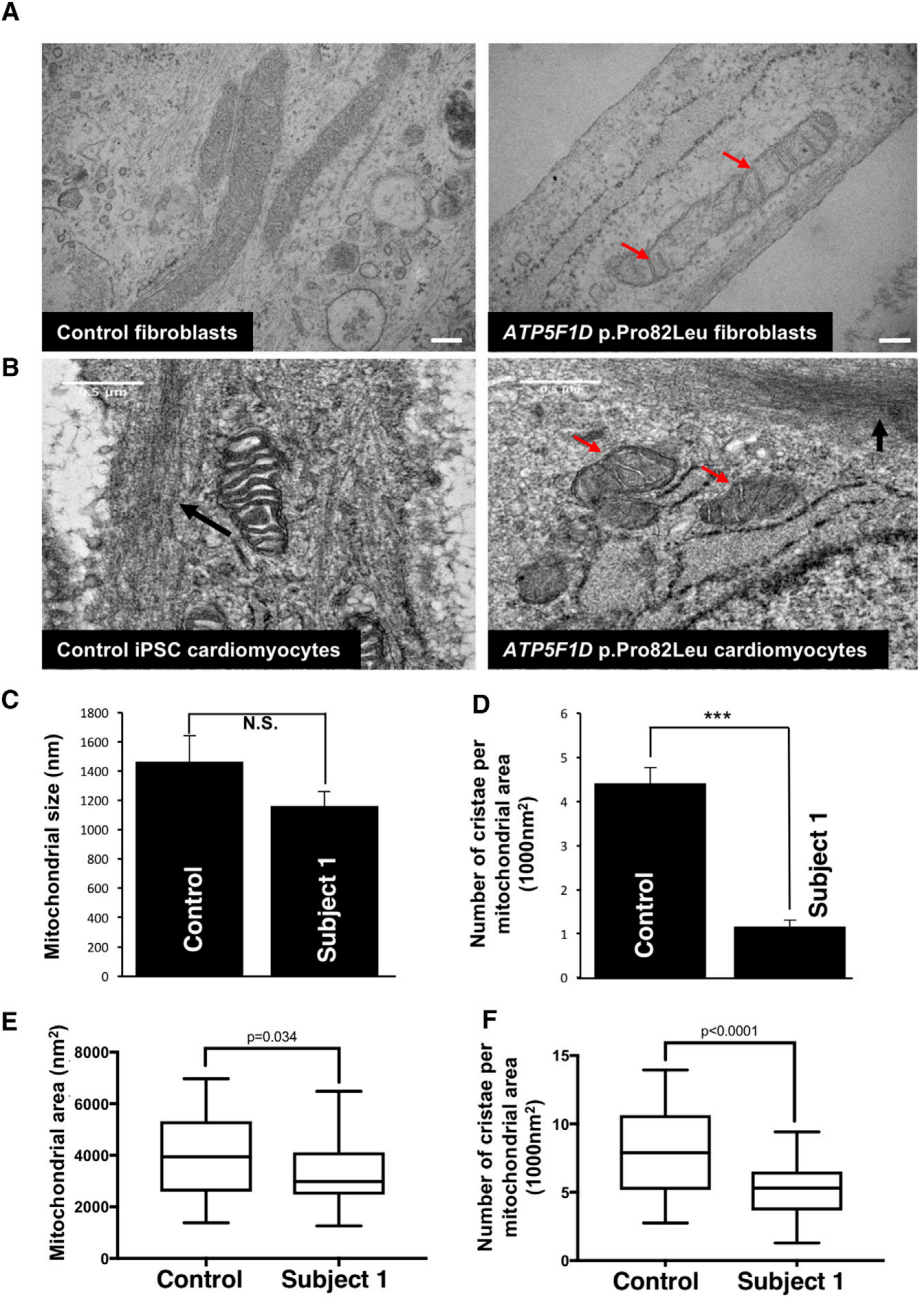
Subject-Derived Cells Carrying a c.245C>T (p.Pro82Leu) *ATP5F1D* Variant Exhibit a Decreased Number of Cristae (A) TEM of cultured skin fibroblasts from an unaffected control individual and subject 1 (S1) (p.Pro82Leu). (B) TEM of iPSC-derived cardiomyocytes. Red arrows show mitochondria devoid of cristae in cells from affected individual S1 (p.Pro82Leu). Black arrows indicate nascent sarcomeres. Scale bar: 500 nm. (C) Quantification of mitochondrial size in control and subject 1 (p.Pro82Leu) fibroblasts. Error bars indicate SEM, and p values were calculated by Student’s t test. N.S. indicates not statistically significant. (D) Quantification of the number of cristae per mitochondrion in control and subject 1 (p.Pro82Leu) fibroblasts. Error bars indicate SEM, and p values were calculated by Student’s t test (^∗∗∗^p < 0.001). (E) Quantification of the mitochondrial area in control and subject 1 (p.Pro82Leu) iPSC-derived cardiomyocytes. Quartiles and minimum and maximum values are shown, and p values were calculated by an unpaired two-tailed t test (p = 0.03). (F) Quantification of the number of cristae per mitochondrion in control and subject 1 (p.Pro82Leu) iPSC-derived cardiomyocytes. Quartiles and minimum and maximum values are shown, and p values were calculated by an unpaired t test (p < 0.001).

To assess mitochondrial morphology, we performed transmission electron microscopy (TEM) on cultured skin fibroblasts of subject 1 (Figure 3A). The mitochondria in these fibroblasts were not significantly different in size from those in control fibroblasts (Figure 3C). However, they displayed a dramatic decrease in the number of cristae (Figures 3A and 3D). Induced pluripotent stem cells (iPSCs) derived from fibroblasts of subject 1 were differentiated into iPSC-derived cardiomyocytes (Figure S5A). These cardiomyocytes exhibited both smaller mitochondrial size and markedly fewer cristae than control cardiomyocytes (Figures 3B, 3E, and 3F), as well as impaired maximal respiration in response to palmitate supplementation (Figure S5B).

To determine whether the defects seen in complex V in subject cells were indeed due to the missense variants found in *ATP5F1D*, we studied the variants in *Drosophila*. *ATP synthase δ subunit* (*ATPsynδ*), the *Drosophila* homolog of *ATP5F1D*, is highly conserved (identity 48%, similarity 65%, DIOPT score 10/12),^19^ and the affected residues (Pro82 and Val106) are also conserved (Figure 1B). We generated transgenic flies harboring a wild-type human cDNA (*UAS-ATP5F1D*^*WT*^) as well as both variant cDNAs (*UAS-ATP5F1D*^*P82L*^ and *UAS-ATP5F1D*^*V106G*^). The expression of these cDNAs can be induced by the transcription factor GAL4.^20^ To knock down the protein, we ubiquitously expressed a UAS-*ATPsynδ* RNAi by using various ubiquitous *Gal4* drivers, including *tub-Gal4*, *Actin-Gal4*, or *da-Gal4*.^21^ All drivers caused lethality (Figure S6C), consistent with previous observations.^22^ Pan-neuronal expression of the *ATPsynδ* RNAi with the *elav*^*[C155]-*^*Gal4* driver resulted in lethality early in development (Figure S6D). This lethality was rescued by expression of human *ATP5F1D*^*WT*^, but not by expression of the two human *ATP5F1D* variants (*ATP5F1D*^*P82L*^ and *ATP5F1D*^*V106G*^) (Figure S6D). These data indicate that human *ATP5F1D* is functional in flies and that the two *ATP5F1D* variants (*ATP5F1D*^*P82L*^ and *ATP5F1D*^*V106G*^) are not fully functional.

**Figure 4:**
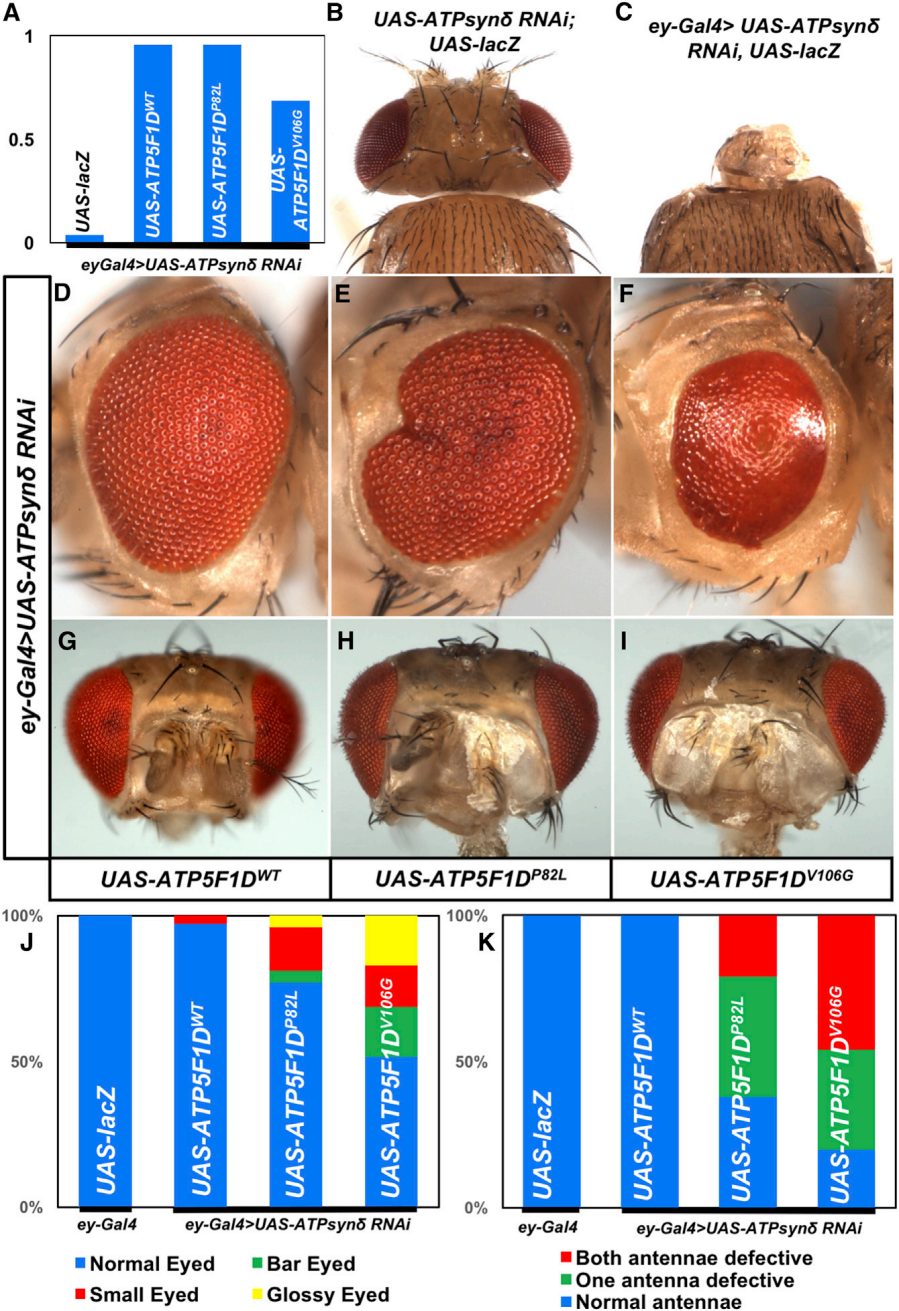
ATP5F1D p.Pro82Leu and p.Val106Gly Are Partial Loss-of-Function Variants (A) The observed/expected ratio of flies shows the rescue of lethality by the human genes including both variants in the *Drosophila* null background. (B and C) Expression of *ATPsyn*δ RNAi by *ey-Gal4* caused pupal lethality and an extremely reduced head size (*ey-Gal4/UAS-ATPsynδ RNAi; UAS-LacZ/+*) (C), whereas control animals without the *ey-Gal4* driver (*UAS-ATPsynδ RNAi/+; UAS-LacZ*) showed normal head development (B). (D–F) Light micrographs of fly eyes expressing *ey-Gal4* and *ATPsynδ* RNAi together with *UAS-ATP5F1D*^*WT*^ (D), *UAS-ATP5F1D*^*P82L*^ (E), or *UAS-ATP5F1D*^*V106G*^ (F). We found that expression of *ATP5F1D*^*WT*^ rescued the tiny-head phenotype caused by knockdown of *ATPsynδ* (D). However, a portion of adult flies expressing *ATPsynδ* RNAi together with *ATP5F1D*^*P82L*^ or *ATP5F1D*^*V106G*^ exhibited abnormal eye morphology, including glassy eyes, small eyes, and bar eyes (E and F). Quantification of the phenotypes shows that expression of *ATP5F1D*^*V106G*^ causes more severe defects than *ATP5F1D*^*P82L*^ (J). (G–I) Light micrographs of fly antenna expressing *ey-Gal4* and *ATPsynδ* RNAi together with *UAS-ATP5F1D*^*WT*^ (G), *UAS-ATP5F1D*^*P82L*^ (H), or *UAS-ATP5F1D*^*V106G*^ (I). (K) Quantification of the antenna morphology phenotypes described in (G)–(I).

To further examine the effect of these variants in adult flies, we used the *eyeless (ey)-Gal4* driver,^23^ whose expression is restricted to the eye, antenna, and part of the brain. Expression of *ATPsynδ* RNAi in the developing eye, brain, and antenna with the *ey-Gal4* driver caused pupal lethality and a near-complete loss of the head (Figures 4A–4C). This lethality and the development of the eye, antenna, and brain were fully rescued by expression of human *ATP5F1D*^*WT*^ (Figure 4A). Expression of the two human *ATP5F1D* variants (*ATP5F1D*^*P82L*^ and *ATP5F1D*^*V106G*^) in flies in which the endogenous ATPsynδ had been knocked down by the *eyGal4* driver rescued lethality (Figure 4A). However, the animals rescued by the *eyGal4* driver retained abnormal eye and antennal phenotypes (Figures 4D–4K). Interestingly, rescue with the *ATP5F1D*^*V106G*^ allele corresponding to subject 2 showed more severe phenotypes than rescue with *ATP5F1D*^*P82L*^—the *ATP5F1D*^*V106G*^ allele only partially rescued lethality, elicited a glossy-eye phenotype less frequently than *ATP5F1D*^*P82*^ expression, and caused more severe defects in electroretinogram recordings than did the *ATP5F1D*^*P82*^ allele (Figure S7). Hence, the mutant ATP5F1D proteins are not fully functional when tested in flies, and the function of *ATP5F1D*^*V106G*^ is more severely affected than *ATP5F1D*^*P82L*^ in this system.

To evaluate the metabolic effects of these mitochondrial defects, we performed exploratory analyses of untargeted plasma metabolite and lipid profiles in samples from subject 1 and in transgenic flies. Plasma metabolomic profiling^24^, ^25^ revealed accumulation of the TCA cycle intermediates malic acid and citric acid, as well as compensatory changes in branched-chain amino acid metabolism (Figure S8 and Table S1). Plasma lipidomic analysis comparing subject 1 samples with those of 136 unrelated control samples revealed increases in long-chain acylcarnitines (C12:1, C14:1, and C16), decreases in dihydroceramides and ceramides, and elevated sphingomyelin, lactosylceramide, and ganglioside (GM3) lipids^26^, ^27^, ^28^, ^29^ (Figure S9A and Table S2). Similar changes in long-chain acylcarnitines were seen in flies with mildly reduced *ATPsynδ* expression driven by attenuated expression of *ATPsynδ* RNAi (C12 and C14:1), wheras alterations in cardiolipin (CL) profile lipids, highly enriched in mitochondrial inner membranes,^30^ (Figure S9B and Table S3) were uniquely observed in fly homogenates. Together, these data suggest that an impairment in mitochondrial fatty acid oxidation might contribute to the hypoglycemia observed in the two subjects.

In summary, we present compelling data that biallelic missense variants in *ATP5F1D* result in a mitochondrial disorder that manifests in childhood with episodic decompensation featuring lactic acidosis and hyperammonemia accompanied by ketoacidosis or hypoglycemia. Chronic manifestations include developmental delay, easy fatiguability, and 3-methylglutaconic aciduria. Interestingly, the two subjects exhibited different ages of onset and differed with respect to the presence of elevated creatine kinase and encephalopathy. Initial clinical studies in both subjects showed normal respiratory chain enzyme profiles (measuring complexes I–IV), and WES was undertaken on account of a clear mitochondrial and/or metabolic phenotype. The pathogenicity of *ATP5F1D* variants (c.245C>T [p.Pro82Leu] and c.317T>G [p.Val106Gly]) identified in these two subjects was confirmed by the segregation of variants with disease in each family (Figure 1), demonstration of severe reduction of complex V activity in subject cultured skin fibroblasts (Figure 2), documentation of fewer mitochondrial cristae in subject cells (Figure 3), and demonstration of incomplete phenotypic rescue by subject *ATP5F1D* variants in *Drosophila* lacking *ATPsynδ* but complete rescue with normal human *ATP5F1D* (Figure 4).

Loss of cristae in mitochondria is consistent with phenotypes associated with other complex V mitochondrial mutants. Indeed, ATP synthase forms dimers and oligomers within the mitochondrial inner membrane, and these oligomers have been shown to be important for cristae formation.^31^, ^32^ Furthermore, individuals with mutations in *MT-ATP6* (MIM: 516060) have disrupted cristae,^33^ and loss of *ATPsynε* (the homolog of human *ATP5F1E*) or *ATPsynγ* (the homolog of human *ATP5F1C*) in flies causes a decreased number of cristae.^22^, ^34^ The glossy-eye phenotype provides an additional link between our observation and OXPHOS genes. Indeed, loss of the NADH dehydrogenase (ubiquinone) PDSW subunit (Pdsw) and cytochrome *c* oxidase subunit of Va (*CoVa*) in the fly eye causes glossy eyes.^35^ These glossy eyes can be considered a “phenolog” or a non-obvious phenotypic link to mitochondrial disease in humans.^36^

Complex V deficiencies have been reported to be due to mutations in the mtDNA-encoded *MT-ATP6*^33^, ^37^ and *MT-ATP8* (MIM: 516070),^38^, ^39^, ^40^ as well as the nuclear-encoded *ATPAF2* (*ATP12* [MIM: 608918])^41^, ^42^ and the F_1_ subunits *ATP5F1E* (*ATP5E* [MIM: 606153])^43^ and *ATP5F1A* (*ATP5A1* [MIM: 164360]).^44^, ^45^ The most common nuclear genetic cause of complex V deficiency, however, is associated with *TMEM70* (MIM: 612418),^46^ which encodes a protein required for the biogenesis and stability of complex V.^47^ The presentation of disorders of complex V has often been described as an early-onset encephalocardiomyopathy that is typically observed in individuals with *TMEM70* mutations.^46^, ^48^, ^49^ However, there can be significant clinical heterogeneity associated with different variants in the same gene: for example, mutations in *MT-ATP6* lead to a variety of clinical syndromes, including neurogenic muscle weakness, ataxia, and retinitis pigmentosa (MIM: 551500), Leigh syndrome (MIM: 256000), mitochondrial infantile bilateral striatal necrosis (MIM: 500003), and Charcot-Marie-Tooth hereditary neuropathy.^50^, ^51^ The findings of hyperammonemia and increased 3-methylglutaconic aciduria in both subjects during acute episodes of metabolic decompensation provides an important phenotypic link to complex V deficiencies because these are also prominent in individuals with *TMEM70*,^46^, ^52^ *ATP5F1E*,^43^ and *ATPAF2*^41^, ^42^ mutations. Proper management of hyperammonemic metabolic crises early in life appears to be vital for improving the prognosis of individuals with *TMEM70* mutations.^46^, ^52^ Persistent 3-methylglutaconic aciduria is also observed in other complex V deficiency syndromes but is additionally seen in a broader range of metabolic disorders.^53^ In summary, the shared and divergent phenotypes observed in our two subjects and the observation that the two variants are both deleterious but to different degrees when tested in *Drosophila* argue for these biallelic mutations in *ATP5F1D* as pathogenic for disease in both subjects.

We anticipate that additional cases of the *ATP5F1D*-related mitochondrial disorder will be identified, providing us with the opportunity to better define the clinical spectrum of the condition. Given the dramatic phenotype associated with the severe loss of *ATP5F1D* function in a model organism (Figure 4),^22^ it is possible that other variants associated with varying phenotypes will also be discovered. At present, the defining features appear to be mild developmental disability, easy fatigability, and episodic biochemical decompensation with acute illness, which can be profound at initial presentation.

## Consortia

The Undiagnosed Diseases Network co-investigators are David R. Adams, Mercedes E. Alejandro, Patrick Allard, Mahshid S. Azamian, Carlos A. Bacino, Ashok Balasubramanyam, Hayk Barseghyan, Gabriel F. Batzli, Alan H. Beggs, Babak Behnam, Anna Bican, David P. Bick, Camille L. Birch, Devon Bonner, Braden E. Boone, Bret L. Bostwick, Lauren C. Briere, Donna M. Brown, Matthew Brush, Elizabeth A. Burke, Lindsay C. Burrage, Shan Chen, Gary D. Clark, Terra R. Coakley, Joy D. Cogan, Cynthia M. Cooper, Heidi Cope, William J. Craigen, Precilla D’Souza, Mariska Davids, Jyoti G. Dayal, Esteban C. Dell’Angelica, Shweta U. Dhar, Ani Dillon, Katrina M. Dipple, Laurel A. Donnell-Fink, Naghmeh Dorrani, Daniel C. Dorset, Emilie D. Douine, David D. Draper, David J. Eckstein, Lisa T. Emrick, Christine M. Eng, Ascia Eskin, Cecilia Esteves, Tyra Estwick, Carlos Ferreira, Brent L. Fogel, Noah D. Friedman, William A. Gahl, Emily Glanton, Rena A. Godfrey, David B. Goldstein, Sarah E. Gould, Jean-Philippe F. Gourdine, Catherine A. Groden, Andrea L. Gropman, Melissa Haendel, Rizwan Hamid, Neil A. Hanchard, Lori H. Handley, Matthew R. Herzog, Ingrid A. Holm, Jason Hom, Ellen M. Howerton, Yong Huang, Howard J. Jacob, Mahim Jain, Yong-hui Jiang, Jean M. Johnston, Angela L. Jones, Isaac S. Kohane, Donna M. Krasnewich, Elizabeth L. Krieg, Joel B. Krier, Seema R. Lalani, C. Christopher Lau, Jozef Lazar, Brendan H. Lee, Hane Lee, Shawn E. Levy, Richard A. Lewis, Sharyn A. Lincoln, Allen Lipson, Sandra K. Loo, Joseph Loscalzo, Richard L. Maas, Ellen F. Macnamara, Calum A. MacRae, Valerie V. Maduro, Marta M. Majcherska, May Christine V. Malicdan, Laura A. Mamounas, Teri A. Manolio, Thomas C. Markello, Ronit Marom, Julian A. Martínez-Agosto, Shruti Marwaha, Thomas May, Allyn McConkie-Rosell, Colleen E. McCormack, Alexa T. McCray, Matthew Might, Paolo M. Moretti, Marie Morimoto, John J. Mulvihill, Jennifer L. Murphy, Donna M. Muzny, Michele E. Nehrebecky, Stan F. Nelson, J. Scott Newberry, John H. Newman, Sarah K. Nicholas, Donna Novacic, Jordan S. Orange, J. Carl Pallais, Christina G.S. Palmer, Jeanette C. Papp, Neil H. Parker, Loren D.M. Pena, John A. Phillips III, Jennifer E. Posey, John H. Postlethwait, Lorraine Potocki, Barbara N. Pusey, Chloe M. Reuter, Amy K. Robertson, Lance H. Rodan, Jill A. Rosenfeld, Jacinda B. Sampson, Susan L. Samson, Kelly Schoch, Molly C. Schroeder, Daryl A. Scott, Prashant Sharma, Vandana Shashi, Edwin K. Silverman, Janet S. Sinsheimer, Kevin S. Smith, Rebecca C. Spillmann, Kimberly Splinter, Joan M. Stoler, Nicholas Stong, Jennifer A. Sullivan, David A. Sweetser, Cynthia J. Tifft, Camilo Toro, Alyssa A. Tran, Tiina K. Urv, Zaheer M. Valivullah, Eric Vilain, Tiphanie P. Vogel, Colleen E. Wahl, Nicole M. Walley, Chris A. Walsh, Patricia A. Ward, Katrina M. Waters, Monte Westerfield, Anastasia L. Wise, Lynne A. Wolfe, Elizabeth A. Worthey, Shinya Yamamoto, Yaping Yang, Guoyun Yu, Diane B. Zastrow, and Allison Zheng.

## Conflicts of Interest

M.S. is a cofounder and member of the scientific advisory board of Personalis, SensOmics, and Qbio. M.S. is a member of the scientific advisory board of Genapsys and Epinomics. J.D.M. is a member of the clinical advisory board for Rainbow Genomics and the scientific advisory board for Genoox. E.A.A. is a founder and member of the scientific advisory board of Personalis and Deepcell. E.A.A. is an advisor to Genome Medical and Sequencebio. M.T.W. has a minor ownership interest in Personalis.

## Accession Numbers

Whole-exome sequencing data from subject 1 has been deposited in dbGaP per the NIH study protocol and subject consent under accession number dbGaP: phs001232.v1.p1. Details of the pathogenic variants in subjects 1 and 2 have been deposited in ClinVar under accession numbers ClinVar: SCV000453296 and SCV000680464. Data and subject-derived biospecimens are available from the corresponding author.

## Web Resources

ClinVar, https://www.ncbi.nlm.nih.gov/clinvar/
dbGaP, https://www.ncbi.nlm.nih.gov/gap
GenBank, https://www.ncbi.nlm.nih.gov/genbank/
genome Aggregation Database (gnomAD) Browser, http://gnomad.broadinstitute.org
OMIM, http://www.omim.org

## Supplemental Data

Document S1. Figures S1–S9 and Tables S1–S3

Document S2. Article plus Supplemental Data
