# Supplementary material for: Biallelic Mutations in ATP5F1D, which Encodes a Subunit of ATP Synthase, Cause a Metabolic Disorder
Source: Am J Hum Genet. 2018 Feb 22;102(3):494–504. doi: 10.1016/j.ajhg.2018.01.020 (PMC6117612; doi:10.1016/j.ajhg.2018.01.020)
Supplement: Document S1. Figures S1–S9 and Tables S1–S3 [file mmc1.pdf]

The American Journal of Human Genetics, Volume *102*

Supplemental Data

Biallelic Mutations in *ATP5F1D*, which Encodes a Subunit of ATP Synthase, Cause a Metabolic Disorder

Monika Oláhová, Wan Hee Yoon, Kyle Thompson, Sharayu Jangam, Liliana Fernandez, Jean M. Davidson, Jennifer E. Kyle, Megan E. Grove, Dianna G. Fisk, Jennefer N. Kohler, Matthew Holmes, Annika M. Dries, Yong Huang, Chunli Zhao, Kévin Contrepois, Zachary Zappala, Laure Frésard, Daryl Waggott, Erika M. Zink, Young-Mo Kim, Heino M. Heyman, Kelly G. Stratton, Bobbie-Jo M. Webb-Robertson, Undiagnosed Diseases Network, Michael Snyder, Jason D. Merker, Stephen B. Montgomery, Paul G. Fisher, René G. Feichtinger, Johannes A. Mayr, Julie Hall, Ines A. Barbosa, Michael A. Simpson, Charu Deshpande, Katrina M. Waters, David M. Koeller, Thomas O. Metz, Andrew A. Morris, Susan Schelley, Tina Cowan, Marisa W. Friederich, Robert McFarland, Johan L.K. Van Hove, Gregory M. Enns, Shinya Yamamoto, Euan A. Ashley, Michael F. Wangler, Robert W. Taylor, Hugo J. Bellen, Jonathan A. Bernstein, and Matthew T. Wheeler

FIGURE S1

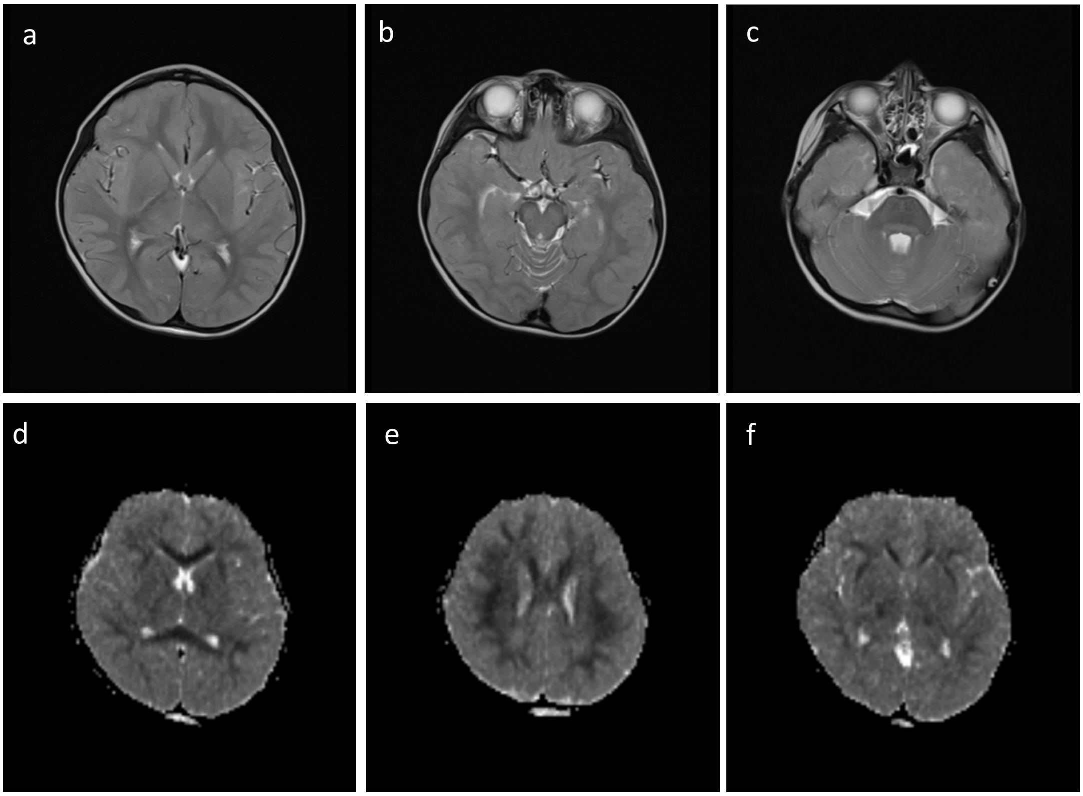

Figure S1. MRI Findings in subject 2

Subject 2 MRI at age 4 years 10 months demonstrated generalised brain swelling (a) with more distinctive subcortical white matter T2 hyperintensity within the temporal lobes bilaterally. There was also distinctive abnormal T2 hyperintensity within the midbrain (b), posterior pons (c) and dentate nuclei. There was a symmetrical pattern of restricted diffusion involving the corpus callosum (d), subcortical white matter of both cerebral hemispheres (e), corticospinal tracts (f), midbrain, pons and cerebellum. All of these changes resolved on follow-up imaging one year later. a = Axial T2SE; b = Axial T2SE; c = Axial T2SE; d = Axial ADC Map; e= Axial ADC Map; f = Axial ADC Map.

FIGURE S2

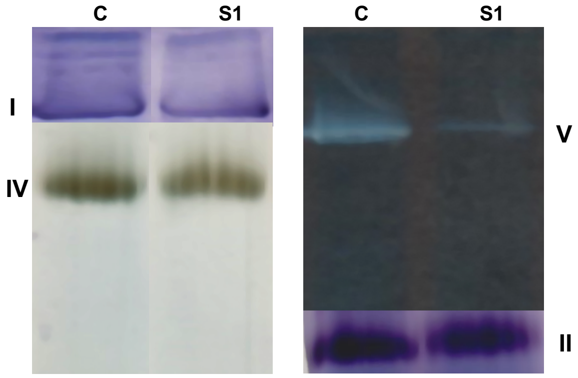

Figure S2. Subject 1 fibroblast studies show reduced in gel activity of complex V. Blue native PAGE with in-gel activity stain performed as described showed reduced activity of complex V in subject fibroblasts[1]. Mitochondrial membrane fractions were isolated from fibroblasts of subject 1 and analyzed by blue native polyacrylamide gel electrophoresis (BN-PAGE) with in-gel activity staining for complexes I, II, IV, and V as indicated for both control (C) and subject 1 (S1). The activity for complex V was reduced in the subject while the activities of complexes I, II, and IV were normal. There were no additional bands of lower molecular weight in complex V, as would be typically seen in disorders affecting the synthesis of the mtDNA-encoded subunits of complex V[1], but similar to that noted with defects in *ATP5F1E*, *ATP5F1A*, and *ATPAF2*, each affecting the assembly of the F_1 subunit.

FIGURE S3

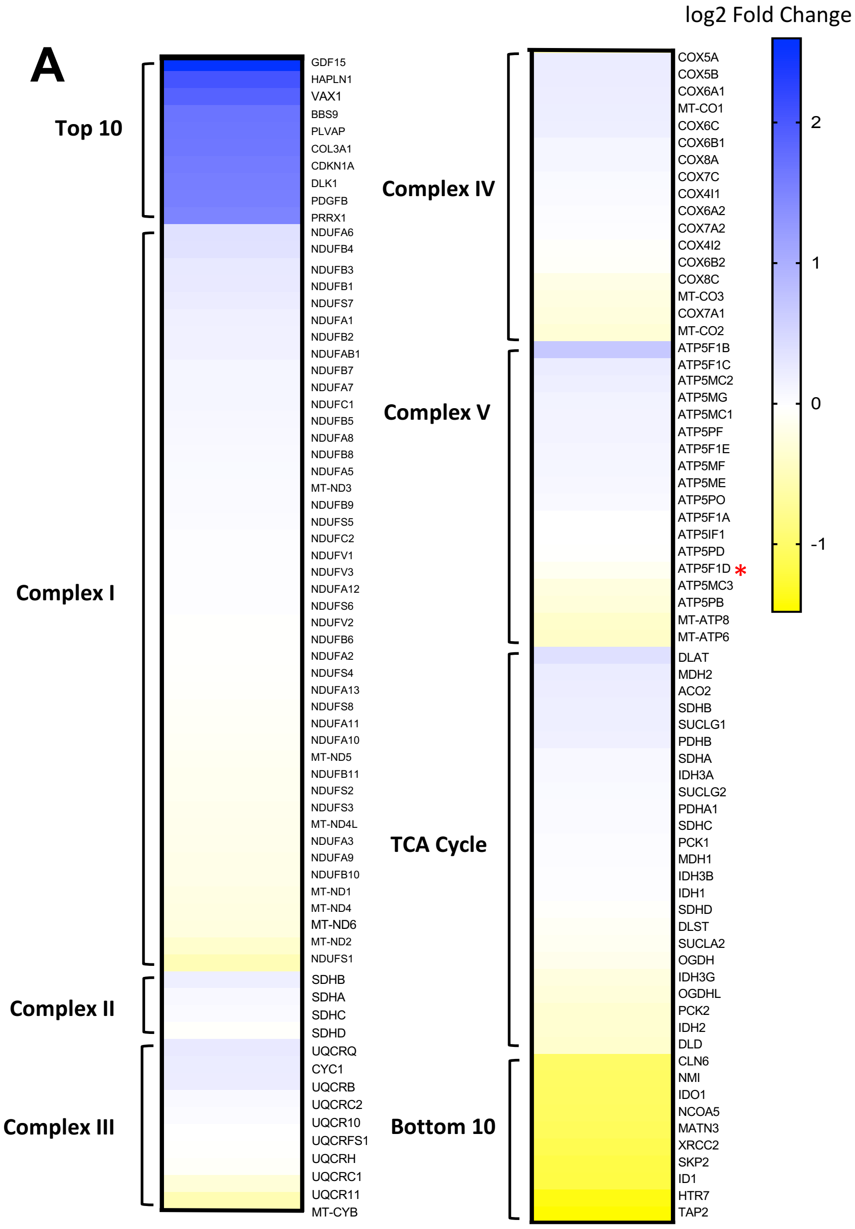

Figure S3. Expression profile of Subject Derived Cells RNA was extracted from cultured iPSC cells utilizing TRIzol. cDNA libraries were constructed using the Illumina TruSeq Stranded mRNA Sample Prep kit. Pooled libraries were run on each a NextSeq 500, using high output flowcells. Sequences were read as paired end 150 cycles (2x150). All base call files (bcl) were converted by in-house script to FASTQ format, compiled and demultiplexed and total read counts were determined for each sample. Initial read quality was determined by utilizing the program FASTQC. All reads were processed by adapter trimming, Kmer removal and the remaining reads mapped to HG19. **(A)** Heat map of RNAseq data from subject 1 (*ATP5F1D*^{P82L}) in comparison to unaffected control samples shows no evidence of dysregulation in *ATP5F1D* expression (red asterisk). Expression levels of other complex I-V components are not significantly altered. Expression is measured via log2 fold change compared to unaffected controls. Top and bottom ten differentially expressed protein coding genes also included. Differential splicing was not seen for *ATP5F1D* transcripts (data not shown). **(B)** Top ten biological processes identified by GO term analysis of top 200 differentially expressed protein coding genes from RNAseq data set. No significant pathways observed on bottom 200 differentially expressed protein coding genes.

B

GO biological process	Actual # Genes in Top 200	Expected in Top 200	Fold Enrichment	P value
positive regulation of endothelial cell migration	7	0.63	11.04	0.04
response to mechanical stimulus	11	1.84	5.97	0.03
extracellular matrix organization	14	2.65	5.29	0.005
regulation of ERK1 and ERK2 cascade	12	2.28	5.26	0.03
angiogenesis	13	2.53	5.14	0.02
response to growth factor	18	4.29	4.19	0.003
positive regulation of apoptotic process	21	5.15	4.08	0.0005
negative regulation of phosphate metabolic process	18	4.95	3.64	0.03
negative regulation of signal transduction	34	9.98	3.41	0.000003
negative regulation of transcription from RNA polymerase II promoter	23	6.98	3.3	0.005

FIGURE S4

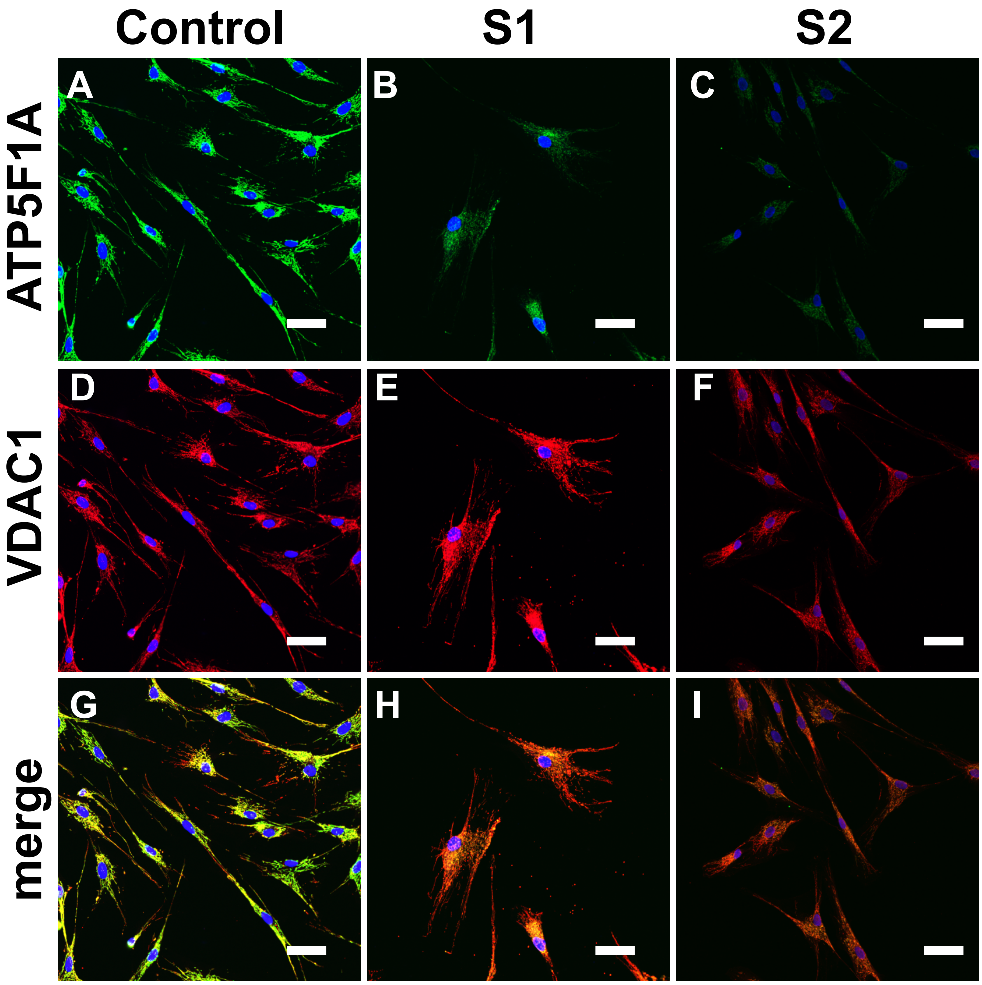

Figure S4: Cultured skin fibroblasts from affected individuals show a complex V defect. Immunofluorescence staining of fibroblasts obtained from affected individuals and control was performed using anti-ATP5F1A antibody (1:1000; ab14748, Abcam, Cambridge, UK) (**A-C**) and anti-VDAC1 (1:400; ab15895, Abcam, Cambridge, UK) (**D-F**), with the overlay (**G-I**) demonstrating strong staining of the complex V protein in the controls and absence in the subject 1 and 2 (S1 and S2) cell lines with preserved mitochondrial voltage dependent anion channel staining in subject cells (Scale bar = 50 µm). Fibroblasts were grown on chamber slides. Cells were allowed to attach for 24 hours. At the next day, the medium was removed, and chamber slides were twice washed with PBS pH 7.4 and fixed in formalin overnight at 4°C. After washing cells three times 3 min with PBS-T (pH 7.5; 0.05% Tween-20), heat-induced epitope retrieval was done in 1 mM EDTA, 0.01% Tween-20, pH 8 at 95°C for 45 min. The solution was allowed to cool down to room temperature and chamber slides were washed with PBS-T. The chamber slides were incubated 1 h at RT with primary antibodies against rabbit-anti-ATP5F1A antibody (1:1000; ab14748, Abcam, Cambridge, UK) and anti-VDAC1 (1:400; ab15895, Abcam, Cambridge, UK). Primary antibodies were diluted in DAKO antibody diluent with background-reducing components. After washing with PBS-T, cells were incubated 1 h at RT in dark with secondary antibodies (Alexa Fluor 594 donkey anti-rabbit antibody, VXA21207, Life Technologies, Carlsbad, US, 1 : 500 and Alexa Fluor 488 donkey anti-mouse IgG (H + L), VXA21202, Carlsbad, US, 1 : 1000). After washing the chamber slides with PBS-T, they were incubated with DAPI diluted 1 : 2000 in PBS-T for 10 min. Chamber slides were mounted in fluorescence mounting media from DAKO.

FIGURE S5

A

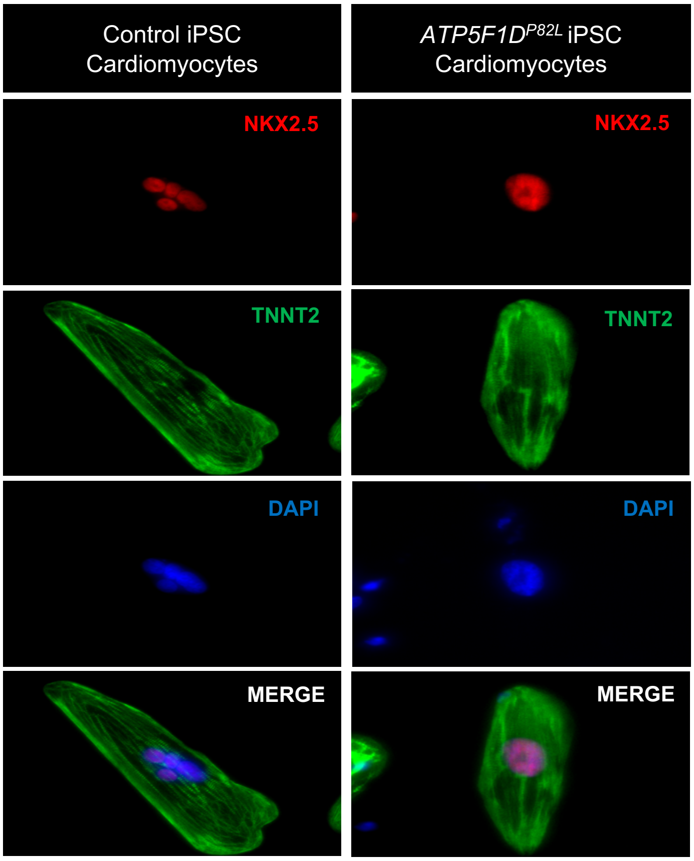

Figure S5. (A) *ATP5F1D^{P82L}* iPSC derived cardiomyocytes iPSCs were reprogrammed with Sendai Virus from subject 1 biopsy skin fibroblast. The iPSCs were then cultured in serum-free/feeder free medium hStemSFM (Stemmera, ST02001) on a matrigel coated plates for 20 passages. 70-80% confluent cells were differentiated to cardiomyocytes we added 2 ml of RPMI medium with B27 supplement minus insulin with 4-6 uM of CHIR-99021 for 2 days. Cells were then treated with RPMI plus B27 minus insulin plus 5uM IWR1 for 2 days, RPMI plus B27 minus insulin for 2 days, then RPMI plus B27 plus insulin for 4 days. To purify the cardiomyocytes, lactate medium has been appliedon day 14. Staining for rabbit anti-NKX2.5 and mouse anti-TTNT2 on day 30 post-differentiation.Control and subject 1 derived (*ATP5F1D^{P82L}*) iPS cells were differentiated into cardiomyocytes, both displaying characteristic staining of TNNT2 and NKX2.5 confirming commitment to cardiomyocyte lineage.

B

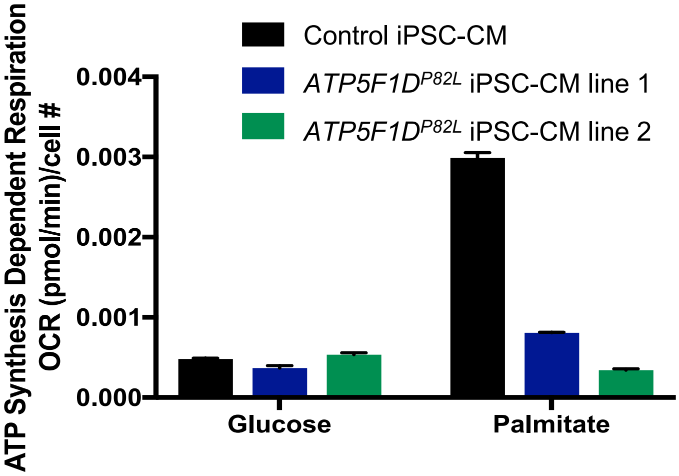

(B) In vitro oxygen consumption assay Seahorse (Agilent Technologies) plate wells were coated with Matrigel overnight. 30,000 Cardiomyocytes per well, differentiated from control and two *ATP5F1D^{P82L}* iPSC lines were plated the following day. Cells were maintained in lactate medium for 3 days. Diluted oligomycin, FCCP and Rotenone/Antimycin a (AA/Rot) were prepared per manufacturer instructions. Cell medium was changed to add glucose or palmitate. Oxygen consumption rate (pmol/min) was determined for each substrate/cell line/drug combination performed in triplicate. Oxygen consumption rate was normalized to viable cell count determined by vital dye staining performed on each well at the completion of the experiment. *ATP5F1D^{P82L}* cardiomyocytes have impaired ATP synthase dependent respiration oxygen consumption rate (OCR) in response to palmitate, when compared to normal cardiomyocytes. Two different iPSC cardiomyocyte lines derived from subject 1 showed decreased ATP synthesis dependent respiration with palmitate as compared to a wildtype control. There was no significant difference in respiration between control and *ATP5F1D^{P82L}* cardiomyocytes when substrate was glucose at nonlimiting concentrations.

FIGURE S6

ey-Gal4> UAS-lacZ

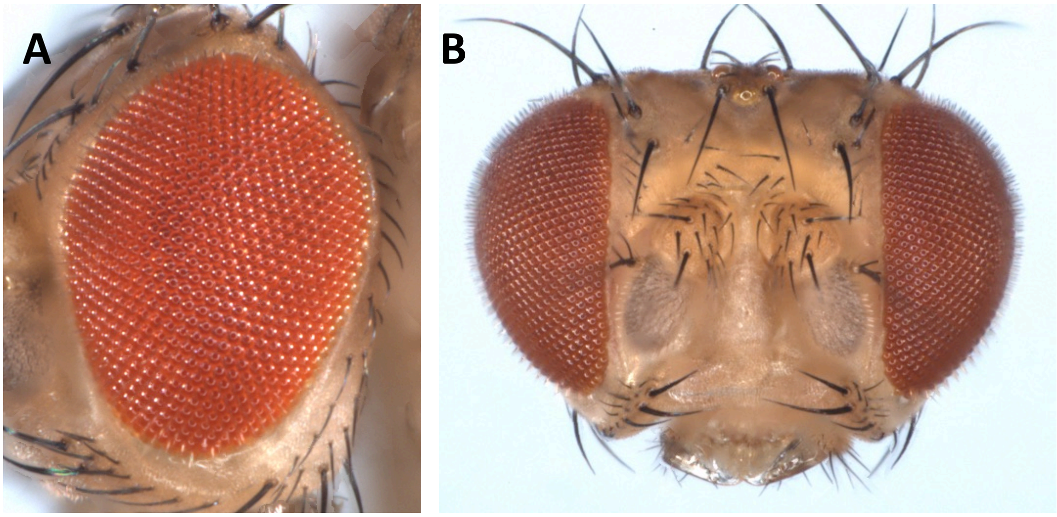

C

Gal4-drivers / Transgene	*Tubulin-Gal4* (ubiquitous)	*Actin-Gal4* (ubiquitous)	*da-Gal4* (ubiquitous)
UAS–empty construct	Viable	Viable	Viable
UAS-ATPsynδ RNAi	Lethal	Lethal	Lethal

D

Transgene	*elav$^{[C155]}$ -Gal4* (neuronal)
UAS-ATPsynδ RNAi	Lethal
UAS-ATPsynδ RNAi; UAS-ATP5F1D^{WT}	Viable
UAS-ATPsynδ RNAi; UAS-ATP5F1D^{P82L}	Lethal
UAS-ATPsynδ RNAi; UAS-ATP5F1D^{V106G}	Lethal

Figure S6: Drosophila overexpression studies.
(**A**-**B**) Light micrographs of an eye (**A**) and antennae (**B**) of flies (*ey-Gal4/+ ; UAS-lacZ/+*) are shown to represent the control eye and antennae morphology. For taking *Drosophila* eye and the antennal images, flies were frozen in -20 °C overnight. Images were obtained using a digital camera (MicroFire; Olympus) mounted on a stereomicroscope (MZ16; Leica) and ImagePro Plus 7.0 acquisition software (Media Cybernetics). The Extended Focus Function of the ImagePro software was used to obtain stacked images. The images were further processed in ImageJ software. **(C)** shows that expression of the *ATPsynδ* RNAi by various ubiquitous *Gal4* drivers including *tub-Gal4*, *Actin-Gal4*, or *da-Gal4* causes lethality, while control transgene (*UAS-empty*) expression does not. **(D)** shows that expression by the neuronal specific driver (*elav$^{[C155]}$-Gal4*) of the *ATPsynδ* RNAi is lethal; this lethality is rescued by human normal *ATP5F1D* and is not rescued by *ATP5F1D* p.P82L or p.V106G variants. See Figure S7 legend for details of ATP5F1D transgenics.

The following stocks were obtained from the Bloomington Stock Center at Indiana University (BDSC).

- y1 w*; tubulin-Gal4/ TM3, Sb1, Ser1
- w*; Actin-Gal4/CyO
- w; da-Gal4 (on III)
- ey-gal4 (on II)
- *w*; Sco/CyO; P{w[+mC]=tubP-GAL80^{ts}}7*
- *w*; P{w[+mC]=tubP-GAL80^{ts}}20; TM2/TM6B, Tb^{1}*

ATPsynδ RNAi lines (v100621) were obtained from the Vienna Drosophila Resource Center.[2] All flies were maintained at room temperature (21°C). All crosses were kept at 25°C except those for the lethality experiment (28°C).

FIGURE S7

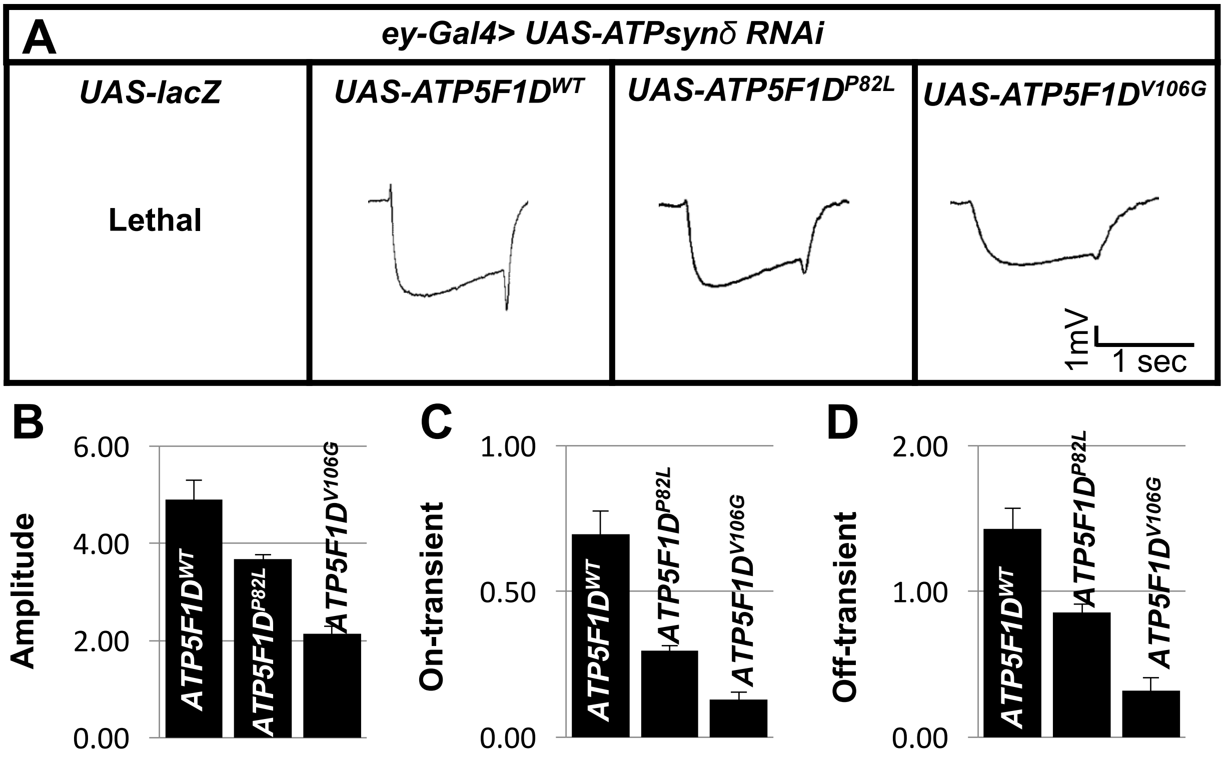

Figure S7: Electroretinogram studies on human ATP5F1D transgene-rescued flies. Plasmids carrying ATP5F1D cDNA with P82L variant and ATP5F1D cDNA with V106G were generated by site-directed mutagenesis PCR from a human ATP5F1D cDNA clone (HsCD00506484, DNASU Plasmid Repository) using primers: ATP5D_p.P82L-F: 5'- cccacgctgcaggtcctgcggcTggggctg-gtcgtggtgcatgca-3', ATP5D_p.P82L-R: 5'- tgcatgcaccacgaccagccccAgccgcaggacctgcagcgtggg-3', ATP5D_p.V106G-F: 5'- gtga-gcagcggttccatcgcagGgaacgccgactcttcggtgcag-3', and ATP5D_p.V106G-R: 5'-ctgcaccgaagagtcggcgttcCctgcgatggaaccgctgctcac-3'. For construction of pUASTattB-human ATP5D-V5, pUASTattB-human ATP5D (p.P82L)-V5, and pUASTattB-human ATP5D (p.V106G)-V5, full-length ATP5F1D cDNAs were amplified by PCR from wild type ATP5F1D cDNA, ATP5F1D (P82L), and ATP5F1D (V106G) clones, and then subcloned into BglII/NotI sites in the pUASTattB vector using primers: ATP5D_F BglII: 5'-AGATCTcaaaATGCTGCCCGCCGCGCTG-3', ATP5D -V5_R NotI: 5'-GCGGCCGCTTAGGTGCTATCCAGTCCGAGCAGTGGATTCGGGA-TCGGCTTGCCGCCGCTTCCCTCCAGGGCCTTCACCAGGG-3'. The pUASTattB constructs were injected into *y,w,FC31; VK33* embryos and transgenic flies were selected.[3] ERG recording was carried out as previously described[4]. Briefly, adult flies were immobilized on a glass slide with glue. A glass-recording electrode, filled with 100 mM NaCl was placed on the surface of the eye, and a glass reference electrode was inserted into the thorax. Recordings were performed after three to four minutes of darkness. A fly eye was exposed to a flash of white light for 1 sec. The responses were digitized and recorded and analyzed with AXON™-pCLAMP8 software. **(A)** Electroretinogram of flies carrying *ey-Gal4 > UAS-ATPsynδ* RNAi, together with *UAS-ATP5F1D^{WT}*, *UAS-ATP5F1D^{P82L}* or *UAS-ATP5F1D^{V106G}*. **(B-D)** Quantification of the electroretinogram shown by amplitude **(B)**, on-transients **(C)**, and off-transients **(D)** of electroretinogram traces in **(A)**. Error bars indicate SEM.

FIGURE S8

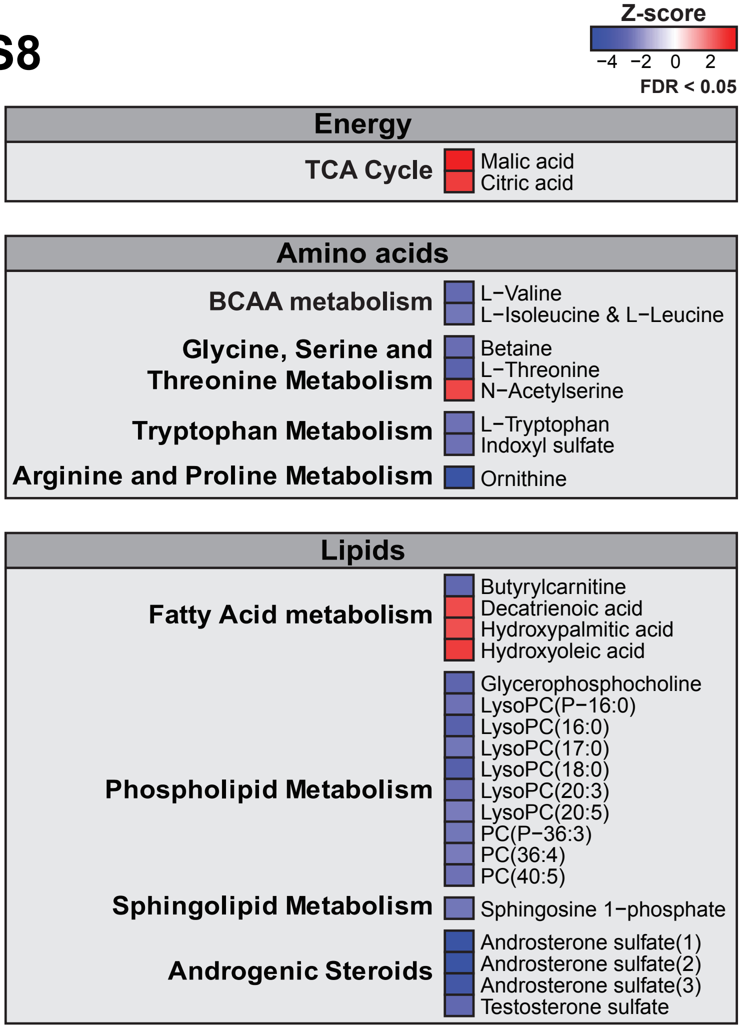

Figure S8. Untargeted plasma metabolomics by complementary HILIC- and RPLC-MS. Outlier analysis in subject 1 in comparison to 21 unrelated controls identified 41 statistically significant metabolites (FDR < 0.05) with MS signal intensity >3E7 (**Table S1**). Metabolites from plasma were extracted and analyzed as previously described.[5,6] Metabolic extracts were analyzed in HILIC ESI (+) MS, HILIC ESI (-) MS, RPLC ESI (+) MS, RPLC ESI (-) MS using a Thermo Ultimate 3000 RSLC system coupled with a Thermo Q Exactive plus mass spectrometer. The Q Exactive plus was equipped with a HESI-II probe and operated in full MS scan mode. MS/MS data were acquired on quality control samples (QCs = equimolar mixture of all the samples comprised in the study). HILIC experiments were performed using a ZIC-HILIC column 2.1 x 100 mm, 3.5 µm, 200Å (Merck Millipore) and mobile phase solvents consisting of 10 mM ammonium acetate in 50/50 acetonitrile/water (A) and 10 mM ammonium acetate in 95/5 acetonitrile/water (B).[5] Metabolites were eluted from the column at 0.5 mL/min using a 1–99% phase A gradient over 15 min. RPLC experiments were performed using a Zorbax SBaq column 2.1 x 50 mm, 1.7 µm, 100Å (Agilent Technologies) and mobile phase solvents consisting of 0.06% acetic acid in water (A) and 0.06% acetic acid in methanol (B). Metabolites were eluted from the column at 0.6 mL/min using a 1–99% phase B gradient over 9 min. Data were analyzed using an in-house data analysis pipeline written in R (version 3.0.1). Metabolite features (characterized by a unique mass/charge ratio and retention time) were extracted, aligned and quantified with the "XCMS" package (version 1.39.4) after conversion of .RAW files to .mzXML using the ProteoWizard MS convert tool. Grouping and annotation were performed with the "CAMERA" package (version 1.16.0). Features from blanks and not present in at least 66% of the samples were discarded. The signal drift with time was corrected by applying LOESS (Local Regression) normalization. After log2 transformation, Z-scores and P-values were calculated for each metabolic feature. P-values were corrected for multiple hypothesis testing using q-value correction. A FDR of 0.05 or less was considered significant. Formal identification of significant metabolites was performed by matching fragmentation spectra to public spectral libraries or by matching retention time and fragmentation spectra to authentic standards when possible.

FIGURE S9

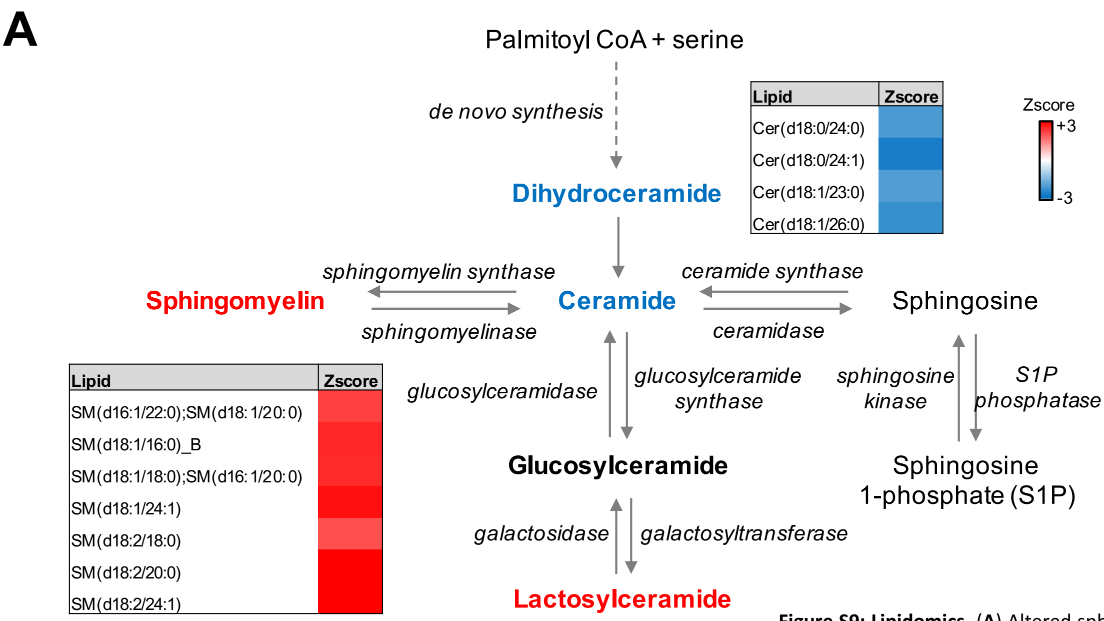

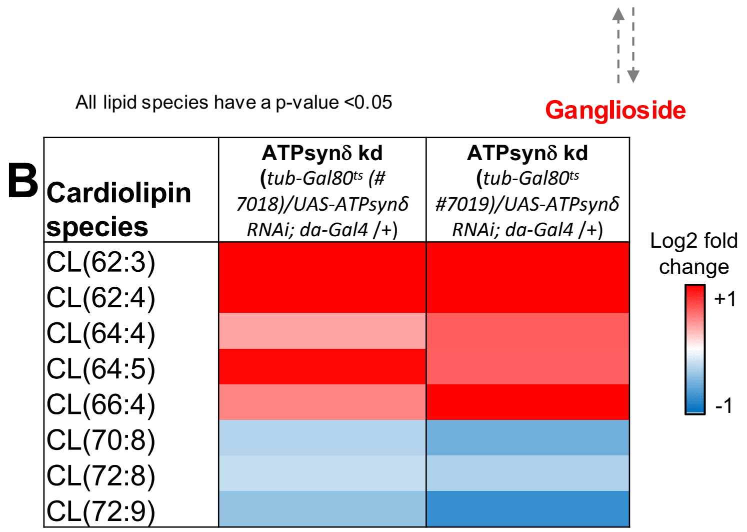

Figure S9: Lipidomics. (**A**) Altered sphingolipid metabolism in subject 1. Dihydroceramide and ceramide lipid species are statistically significantly lower (blue; p-value <0.05) while more complex lipids, in which ceramide is a precursor, are elevated (red). Heat maps represent z-score values of statistically significant lipids (p-value <0.05). Sphingosine and sphingosine-1-phosphate were not detected. (**B**) Cardiolipin species identified in *ATPsynδ* knockdown female flies (*tub-Gal80^{ts} (Bloomington # 7018 and #7019)/UAS-ATPsynδ RNAi; da-Gal4* /+) and control flies (*tub-Gal80^{ts} (Bloomington #7019)/UAS-ATPsynδ RNAi*). As ubiquitous expression of *ATPsynδ* RNAi causes lethality, we used *tub-Gal80^{ts}* to knockdown expression of *ATPsynδ RNAi.* The cardiolipin species are organized by increasing total number of carbons and double bonds in the fatty acids. Heat maps represent log2 fold changes.

Subject 1 sample was compared to a reference database of 136 individuals that were between the ages of 0.6 to 81 years and 50% female with no known metabolic disease. In order to correct for batch effects, we included identical quality control (QC) samples in both the reference dataset and in subsequent subject datasets. Lipids were extracted by using an established chloroform/methanol extraction procedure based on a modified Folch extraction (MPLEx).[7] For both the reference and subject plasma samples, 50 µl of plasma was transferred to 2.0 mL Sorenson low-binding microcentrifuge tubes to which 250 µl of cold (-20°C) chloroform/methanol (2:1, v/v) was added. Samples are vortexed for 10 s and incubated at 4°C for 5 minutes, and then vortexed again for 10 s. Then, samples are centrifuged to facilitate separation of a hydrophilic layer containing polar metabolites and a hydrophobic layer containing lipids. The hydrophobic lipid layer was removed and placed into new microcentrifuge tubes and evaporated to dryness in vacuo. Lipid extracts are stored at -20°C in chloroform/methanol (2:1, v/v) until LC-MS analysis. Prior to MS analysis, total lipid extracts (TLEs) were dried and then reconstituted in 200 µl of methanol. LC-MS/MS parameters and lipid identifications are outlined in Kyle et al. (2017).[8] Reconstituted lipids were analyzed using a Waters Aquity UPLC H class system interfaced with a Velos-ETD Orbitrap mass spectrometer is used for LC-ESI-MS/MS analyses. A Waters CSH column (3.0 mm x 150 mm x 1.7 µm particle size) is used to separate lipid molecular species over a 34 min gradient (mobile phase A: ACN/H_2O (40:60) containing 10 mM ammonium acetate; mobile phase B: ACN/IPA (10:90) containing 10 mM ammonium acetate) at a flow rate of 250 µl/min. Eluting lipids are introduced to the MS via electrospray ionization in both positive and negative modes, and lipids are fragmented using HCD (higher-energy collision dissociation) and CID (collision-induced dissociation) to obtain high coverage of the lipidome. Lipid identifications were made using in-house developed identification software LIQUID where the tandem mass spectra were examined for diagnostic ion fragments along with associated hydrocarbon chain fragment information.[8] In addition, the isotopic profile, extracted ion chromatogram, and mass measurement error of precursor ions were examined for each lipid species. To facilitate quantification of lipids, a reference database for lipids identified from the MS/MS data was created, containing lipid name, observed *m/z*, and retention time. Lipid features from each analysis were then aligned to the reference database based on their *m/z* and retention time using MZmine 2.[9] Aligned features were manually verified and peak apex intensity values were exported for subsequent statistical analysis.

Table S1

Table S1. Significant plasma metabolites in subject 1 (FDR < 0.05).

Ionization mode	Mode	Retention time (min)	Metabolite	PATHWAY	KEGG	HMDB	Species	Z-score	FDR	Median Intensity	Mass error (ppm)
ESI (-) MS	nHILIC	10.3	Malic acid	TCA Cycle	C00149	HMDB00156	[M-H]-	3.9	2.73E-03	2.8E+08	-0.6
ESI (-) MS	nHILIC	11	Citric acid	TCA Cycle	C00158	HMDB00094	[M-H]-	3.0	2.12E-02	2.5E+09	-0.7
ESI (-) MS	nHILIC	7.5	L-Valine	branched chain AA Metabolism	C00183	HMDB00883	[M-H]-	-3.1	1.87E-02	5.5E+08	-0.1
ESI (+) MS	pHILIC	6.5	L-Isoleucine|L-Leucine	branched chain AA Metabolism	C00407 C00123	HMDB00172 HMDB00687	[M+H]+	-2.8	3.35E-02	1.7E+09	-1.8
ESI (+) MS	pHILIC	6.8	Betaine	Glycine, Serine, Threonine Metabolism	C00719	HMDB00043	[M+H]+	-2.9	2.56E-02	4.3E+07	-0.8
ESI (-) MS	nHILIC	8.9	L-Threonine	Glycine, Serine, Threonine Metabolism	C00188	HMDB00167	[M-H]-	-3.5	1.02E-02	5.4E+08	-0.5
ESI (-) MS	nHILIC	7.7	N-Acetylserine	Glycine, Serine, Threonine Metabolism		HMDB02931	[M-H]-	2.8	3.35E-02	7.4E+07	0.6
ESI (+) MS	pHILIC	6.8	L-Tryptophan	Tryptophan Metabolism	C00078	HMDB00929	[M+H]+	-2.9	2.90E-02	7.1E+08	-1.6
ESI (-) MS	nHILIC	3.4	Indoxyl sulfate	Tryptophan Metabolism		HMDB00682	[M-H]-	-2.9	2.83E-02	1.9E+09	-1.3
ESI (+) MS	pHILIC	16	Ornithine	Arginine and Proline Metabolism	C00077	HMDB03374	[M+H]+	-5.4	9.22E-06	4.0E+08	-2.2
ESI (+) MS	pHILIC	8.3	Butyrylcarnitine	Fatty Acid Metabolism	C02862	HMDB02013	[M+H]+	-3.2	1.69E-02	8.3E+07	-3.2
ESI (-) MS	nRPLC	7	Decatrienoic acid	Fatty Acid Metabolism			[M-H]-	2.7	3.63E-02	4.7E+07	0.1
ESI (-) MS	nRPLC	10.3	Hydroxypalmitic acid	Fatty Acid Metabolism			[M-H]-	2.7	4.09E-02	3.3E+07	0.2
ESI (-) MS	nRPLC	10.2	Hydroxyoleic acid	Fatty Acid Metabolism			[M-H]-	3.0	2.37E-02	3.7E+07	0.3
ESI (+) MS	pHILIC	10.4	Glycerophosphocholine	Phospholipid Metabolism	C00670	HMDB00086	[M+H]+	-3.3	1.33E-02	4.4E+08	-2.4
ESI (+) MS	pRPLC	10.2	LysoPC(P-16:0)	Phospholipid Metabolism	C04230	HMDB10407	[M+H]+	-2.9	2.79E-02	4.3E+07	-2.3
ESI (+) MS	pRPLC	9.9	LysoPC(16:0)	Phospholipid Metabolism	C04230	HMDB10382	[M+H]+	-3.5	8.97E-03	4.0E+09	-1.1
ESI (+) MS	pRPLC	10.3	LysoPC(17:0)	Phospholipid Metabolism	C04230	HMDB12108	[M+H]+	-2.8	3.52E-02	7.1E+07	-0.8
ESI (+) MS	pRPLC	10.6	LysoPC(18:0)	Phospholipid Metabolism			[M+H]+	-3.7	5.68E-03	7.7E+08	0.8
ESI (+) MS	pHILIC	5.9	LysoPC(20:3)	Phospholipid Metabolism	C04230	HMDB10393	[M+H]+	-3.0	2.45E-02	4.8E+07	-1.3
ESI (+) MS	pRPLC	9.5	LysoPC(20:5)	Phospholipid Metabolism	C04230	HMDB10397	[M+H]+	-2.6	4.63E-02	4.0E+07	0.9
ESI (+) MS	pHILIC	4.7	PC(P-36:3)	Phospholipid Metabolism			[M+H]+	-2.8	3.54E-02	7.0E+07	-3.6
ESI (+) MS	pHILIC	4.7	PC(36:4)	Phospholipid Metabolism			[M+H]+	-2.7	4.26E-02	8.5E+08	-4.3
ESI (+) MS	pHILIC	4.6	PC(40:5)	Phospholipid Metabolism	C00157		[M+H]+	-2.8	3.12E-02	4.1E+07	-4.9
ESI (+) MS	pRPLC	9.4	Sphingosine 1-phosphate	Sphingolipid Metabolism	C06124	HMDB00277	[M+H]+	-2.7	3.81E-02	5.8E+07	-1.7
ESI (-) MS	nRPLC	9.3	Androsterone sulfate(1)	Androgenic Steroids		HMDB02759	[M-H]-	-5.3	1.42E-05	1.4E+08	0.6
ESI (-) MS	nRPLC	9.6	Androsterone sulfate(2)	Androgenic Steroids		HMDB02759	[M-H]-	-5.4	9.22E-06	3.8E+08	0.6
ESI (-) MS	nRPLC	9.9	Androsterone sulfate(3)	Androgenic Steroids		HMDB02759	[M-H]-	-4.5	3.65E-04	3.4E+07	0.7
ESI (-) MS	nRPLC	9	Testosterone sulfate	Androgenic Steroids		HMDB02833	[M-H]-	-3.2	1.65E-02	1.3E+09	1.9
ESI (+) MS	pRPLC	8.6	Piperine	Food Component/Plant	C03882	HMDB29377	[M+H]+	-2.7	3.95E-02	1.3E+08	-1.6
ESI (-) MS	nHILIC	11.3	C10H14N2O7				[M-H]-	3.0	2.08E-02	6.8E+07	-0.5
ESI (-) MS	nRPLC	9	C26H26O7				[M-H]-	-3.2	1.56E-02	3.4E+07	3.0
ESI (-) MS	nHILIC	10.8	C6H10O8				[M-H]-	-3.2	1.61E-02	5.2E+07	-0.1
ESI (-) MS	nHILIC	10.5	C6H9NO6				[M-H]-	-2.6	4.98E-02	7.8E+08	-1.0
ESI (-) MS	nHILIC	10.5	C7H14N2O4				[M-H]-	2.6	4.88E-02	7.9E+07	-0.7
ESI (+) MS	pHILIC	10.3	C8H16N2O4				[M+H]+	3.1	1.87E-02	4.8E+07	-1.9
ESI (-) MS	nHILIC	3.6	C9H14N2O6				[M-H]-	3.4	1.10E-02	1.6E+08	-0.9
ESI (-) MS	nHILIC	10.5	C9H14N2O7				[M-H]-	2.9	2.56E-02	1.2E+08	-1.7
ESI (-) MS	nHILIC	11.3	C9H16N2O6				[M-H]-	3.4	1.02E-02	7.9E+07	0.0
ESI (-) MS	nHILIC	11.2	Thiosulfic acid			HMDB60293	[M-H]-	2.7	4.33E-02	1.3E+08	-1.0
ESI (+) MS	pHILIC	7.4	C4H9N				[M+H]+	-2.8	3.38E-02	4.1E+08	-0.3

Identifications are confirmed by matching MS/MS spectra to spectral libraries or references when available. Elemental composition was determined using isotopic distribution and accurate mass. Androsterone sulfate elutes in multiple peaks - labeled 1, 2 and 3.

Table S2

Table S2. Statistically significant lipids identified in subject 1 ($p<0.05$)

Lipid Common Name	Fold Change	Zscore	Pvalue
carnitine(12:1)	2.67	2.51	1.21E-02
carnitine(14:1)	3.20	2.73	6.33E-03
carnitine(16:0)	1.46	2.47	1.33E-02
Palmitoyl-EA (endocannabinoid)	1.20	2.30	2.13E-02
CE(18:1)	0.63	2.05	4.00E-02
CE(18:2)	1.01	3.26	1.12E-03
Cer(d18:0/24:0)	-0.91	-2.10	3.56E-02
Cer(d18:0/24:1)	-1.76	-2.71	6.75E-03
Cer(d18:1/23:0)	-1.01	-2.04	4.16E-02
Cer(d18:1/26:0)	-1.29	-2.34	1.94E-02
SM(d16:1/22:0);SM(d18:1/20:0)	0.85	2.24	2.53E-02
SM(d18:1/16:0)	0.82	2.55	1.08E-02
SM(d18:1/18:0);SM(d16:1/20:0)	1.06	2.52	1.19E-02
SM(d18:1/24:1)	1.23	2.83	4.70E-03
SM(d18:2/18:0)	1.01	2.06	3.93E-02
SM(d18:2/20:0)	1.47	3.18	1.46E-03
SM(d18:2/24:1)	1.54	3.48	5.03E-04
PC(20:2/0:0)	1.41	1.98	4.72E-02
PC(0:0/20:4)	1.31	2.08	3.78E-02
PC(16:0/16:0)	1.16	2.91	3.60E-03
PC(16:0/18:1)	0.97	3.34	8.24E-04
PC(16:1/18:1);PC(16:0/18:2)	0.94	3.24	1.20E-03
PC(18:0/18:2)	0.93	3.36	7.83E-04
PC(18:1/18:2)	0.96	2.49	1.28E-02
PC(16:0/20:4)	0.75	2.31	2.08E-02
PC(18:0/20:4);PC(18:1/20:3)	0.97	2.71	6.80E-03
PC(16:0/22:6)	0.98	2.01	4.42E-02
PC(20:0/20:4)	1.10	2.16	3.09E-02
PC(20:2/20:4);PC(18:1/22:5)	1.40	3.12	1.80E-03
PC(O-34:1);PC(P-34:0)	1.28	2.92	3.50E-03
PC(O-18:1/18:2);PC(P-18:0/18:2)	1.47	2.24	2.50E-02
PE(16:0/18:0)	0.89	2.06	3.96E-02
PE(18:1/20:4)_A;PE(16:0/22:5)	1.32	2.32	2.04E-02
PE(O-18:0/18:1);PE(P-20:0/16:0)	1.03	2.53	1.14E-02
PE(O-18:0/20:4)	-1.04	-2.09	3.62E-02
PE(P-16:0/20:3)	-1.42	-2.23	2.56E-02
PI(16:0/18:1);PI(16:1/18:0)	-1.43	-2.25	2.47E-02
PI(16:0/20:3);PI(18:1/18:2)	-1.60	-2.36	1.81E-02
PI(16:0/20:4)	-1.61	-2.45	1.42E-02
PI(18:1/20:4)	-2.10	-2.17	2.99E-02
TG(14:0/16:0/18:1);TG(16:0/16:0/16:1)	-2.00	-2.07	3.87E-02
TG(48:1)	-2.00	-2.07	3.87E-02
TG(16:1/18:1/18:2);TG(16:0/18:2/18:2);TG(16:0/18:1/18:3)	0.99	2.11	3.49E-02
TG(56:4)	1.75	2.85	4.31E-03
TG(18:1/18:2/20:4);TG(16:0/18:2/22:5)	1.30	2.36	1.82E-02
TG(18:2/18:2/20:4);TG(18:1/18:3/20:4);TG(18:1/18:2/20:5)	1.54	2.10	3.59E-02
TG(18:0/18:1/22:0);TG(16:0/18:1/24:0)	-2.07	-1.96	4.99E-02

Lipid common name annotation ZZ(X1:Y1/X2:Y2) where ZZ = lipid class; X1 = number of carbons in chain 1; Y1 = number of double bonds in chain 1; X2 = number of carbons in chain 2; Y1 = number of double bonds in chain 2. Identifications with ZZ(X:Y) denotes the total number of carbons (X) and double bonds (Y) in the chains. CE = cholesterol ester; Cer = ceramide; SM = sphingomyelin; PC = glycerophosphocholine; PCO and PCP = alkyl and alkenyl glycerophosphocholine, respectively; PE = glycerophosphoethanolamine; PEO and PEP = alkyl and alkenyl glycerophosphoethanolamine, respectively; PI = glycerophosphoinositol; TG = triacylglycerol. Zscore coloring scales from +3 (red) to -3 (blue).

Table S3

Table S3. Statistically significant lipids identified in female flies ($p<0.05$)

Lipid Common Name	*ATPsynδ* Kd (#7018)	*ATPsynδ* Kd (#7019)	*ATPsynδ* Kd (#7018)	*ATPsynδ* Kd (#7019)
	log2 fold change		p-value	
carnitine(12:0)	0.885	0.839	0.0017	0.0015
carnitine(14:1)	0.705	1.065	0.0155	0.0009
carnitine(16:0)	-0.639	-0.431	0.0369	0.1179
Cer(d14:0/20:0)	0.453	0.373	0.0202	0.0340
Cer(d14:1/18:0)	0.039	0.407	0.8978	0.0036
Cer(d14:1/20:0)	-0.059	0.426	0.8508	0.0092
Cer(d16:1/22:1)	0.210	0.193	0.0209	0.0213
Cer(d14:2/22:0)	0.244	0.144	0.0210	0.1240
PE_Cer(d14:1/22:0)	0.008	0.123	0.9785	0.0307
PE_Cer(d14:2/24:0)	0.140	0.082	0.0439	0.1959
PE_Cer(d16:1/24:0)	-0.260	-0.255	0.0610	0.0474
CL(62:3)	1.406	1.226	0.0023	0.0033
CL(62:4)	1.466	1.333	0.0110	0.0119
CL(64:4)	0.366	0.642	0.0847	0.0041
CL(64:5)	0.980	0.629	0.0380	0.1407
CL(66:4)	0.486	1.075	0.3417	0.0201
CL(70:8)	-0.295	-0.559	0.3376	0.0391
CL(72:8)	-0.237	-0.316	0.1165	0.0279
CL(72:9)	-0.415	-0.790	0.0167	0.0002
PC(20:0/0:0)	-0.199	-0.418	0.3310	0.0240
PC(12:0/12:0)	1.052	0.523	0.0000	0.0011
PC(12:0/13:0)	0.958	0.493	0.0092	0.1135
PC(12:0/14:0)	0.838	0.687	0.0056	0.0107
PC(12:0/14:1)	0.913	0.624	0.0014	0.0081
PC(14:0/14:0)	0.534	0.274	0.0001	0.0035
PC(12:0/16:1)	0.829	0.567	0.0006	0.0041
PC(14:0/15:0)_B;PC(13:0/16:0)	0.157	-0.590	0.3554	0.0011
PC(14:0/16:1)	0.405	0.295	0.0023	0.0091
PC(14:1/16:0)	0.368	0.420	0.0306	0.0108
PC(14:1/16:1)	0.729	0.363	0.0029	0.0630
PC(30:3)	0.811	0.373	0.0012	0.0484
PC(15:0/16:0);PC(14:0/17:0)	-0.043	-0.588	0.9505	0.0073
PC(15:0/16:1)	0.066	-0.395	0.8160	0.0134
PC(16:0/16:1)	0.159	0.217	0.1225	0.0268
PC(14:0/18:2);PC(16:1/16:1)	0.308	0.080	0.0021	0.3280
PC(14:1/18:2)	1.136	0.537	0.0009	0.0356
PC(16:0/17:0)	-0.092	-0.317	0.6731	0.0347
PC(15:0/18:2)	-0.101	-0.588	0.6001	0.0009
PC(16:1/17:1)	-0.036	0.328	0.8492	0.0023
PC(16:0/18:1)	0.000	0.151	1.0000	0.0043
PC(16:1/18:1)	0.096	0.185	0.1263	0.0048
PC(17:0/18:1)	-0.216	-0.443	0.2168	0.0099
PC(17:0/18:2)	-0.254	-0.578	0.1168	0.0015
PC(18:0/18:1)	0.185	0.297	0.1623	0.0198
PC(18:0/18:2)	0.089	-0.180	0.3820	0.0410
PC(18:1/18:1)	0.041	0.217	0.8002	0.0205
PC(18:2/18:3)	0.030	-0.506	0.9445	0.0016
PC(18:3/18:3)	-0.177	-0.578	0.6176	0.0275
PC(17:1/20:2);PC(18:2/19:1)	-0.410	-0.309	0.0053	0.0156
PC(18:1/20:0)	-0.354	-0.134	0.0490	0.4713
PC(18:2/20:0)	-0.542	-0.475	0.0092	0.0121
PC(18:1/20:2);PC(18:2/20:1)	-0.266	-0.252	0.0156	0.0138
PC(18:2/20:2)	-0.355	-0.480	0.0362	0.0052
PC(18:1/21:0)	-0.791	-0.516	0.0049	0.0294
PC(O-18:1/18:2)	0.588	0.015	0.0193	0.9938

Table S3. Continued

Lipid Common Name	*ATPsynδ* Kd (#7018)	*ATPsynδ* Kd (#7019)	*ATPsynδ* Kd (#7018)	*ATPsynδ* Kd (#7019)
	log2 fold change		p-value	
PE(12:0/0:0)	0.525	0.797	0.1959	0.0351
PE(14:0/0:0)	0.354	0.672	0.2988	0.0296
PE(12:0/12:0)	0.503	0.532	0.0484	0.0265
PE(12:0/14:0)	0.413	0.699	0.1019	0.0067
PE(14:0/14:1);PE(12:0/16:1)	0.915	0.766	0.0052	0.0090
PE(14:1/16:1);PE(12:0/18:2)	0.722	0.405	0.0045	0.0519
PE(15:0/16:0);PE(14:0/17:0)	-0.270	-0.804	0.2740	0.0021
PE(15:0/16:1)	0.010	-0.620	0.9986	0.0275
PE(15:1/16:1)	0.456	0.194	0.0132	0.2307
PE(16:0/16:0)	-0.520	-0.644	0.0919	0.0282
PE(16:0/17:0)	-0.319	-0.539	0.0734	0.0040
PE(15:0/18:2)	-0.455	-0.345	0.0133	0.0341
PE(16:0/18:1)	-0.055	0.230	0.4936	0.0023
PE(17:0/18:1)	-0.372	-0.185	0.0456	0.2926
PE(17:1/18:2)	-0.384	-0.286	0.0236	0.0590
PE(18:3/18:3)	-0.167	-0.698	0.7492	0.0315
PE(18:1/19:0)	-0.167	-0.278	0.2670	0.0419
PE(18:1/19:1);PE(18:2/19:0)	0.261	0.014	0.0266	0.9773
PE(18:2/19:1)	-0.099	-0.445	0.7284	0.0175
PE(18:2/20:0)	-0.360	-0.361	0.0324	0.0220
PE(18:2/20:2)	-0.350	-0.547	0.1313	0.0157
PE(18:1/21:0)	-0.829	-0.385	0.0004	0.0238
PE(18:2/21:0)	-1.077	-0.695	0.0001	0.0013
PE(18:1/22:0)	-0.822	-0.269	0.0005	0.1076
PE(18:2/22:0)	-0.913	-0.759	0.0001	0.0001
PE(24:0/18:1)	-0.701	-0.157	0.0036	0.4725
PE(O-16:0/16:1)	0.091	0.299	0.4984	0.0095
PE(O-18:0/16:1)	0.338	0.233	0.0328	0.1005
PE(O-20:0/18:1)	0.580	0.451	0.0016	0.0045
PE(P-16:0/18:2)	-0.352	-0.100	0.0271	0.5610
PE(P-18:0/16:1)	0.122	0.362	0.3255	0.0036
PE(P-18:0/18:1)	0.256	0.247	0.0021	0.0016
PE(P-20:0/18:1)	0.672	0.605	0.0020	0.0023
PE(P-20:0/18:3)	-0.107	-0.302	0.5250	0.0259
PG(14:0/14:0)	1.176	0.421	0.0032	0.1901
PG(14:0/14:1);PG(12:0/16:1)	0.743	1.108	0.0743	0.0082
PG(14:0/16:0)	0.638	0.293	0.0282	0.2760
PG(14:0/16:1)	0.621	0.443	0.0221	0.0659
PG(16:1/17:1);PG(15:0/18:2)	0.418	0.609	0.0784	0.0100
PG(16:0/18:1)	-0.026	0.370	0.9612	0.0126
PG(16:0/19:1)			0.0040	0.0017
PG(18:1/18:1)	-0.121	0.451	0.7017	0.0320
PI(12:0/14:0)	1.032	0.315	0.0018	0.2227
PI(14:0/14:0);PI(12:0/16:0)	0.605	0.012	0.0338	0.9972
PI(12:0/16:1)	0.885	0.511	0.0020	0.0256
PI(14:0/16:0)	0.639	0.286	0.0273	0.2860
PI(14:0/16:1)	0.736	0.321	0.0002	0.0181
PI(14:1/16:1)	1.084	0.636	0.0073	0.0615
PI(16:1/16:1)	0.479	0.166	0.0130	0.3461
PI(16:1/17:1)	0.369	0.002	0.0476	0.9998
PI(16:0/18:2)	-0.030	-0.172	0.8442	0.0266
PI(16:0/18:3)	-0.179	-0.306	0.1839	0.0183
PI(18:1/18:1)	0.150	0.337	0.2833	0.0115
PI(18:2/18:2)	0.218	-0.407	0.1037	0.0039
PI(18:2/18:3)	0.117	-0.565	0.6676	0.0062
PI(18:3/18:3)	-0.061	-0.626	0.9433	0.0222
PI(18:2/20:2)	-0.852	-0.913	0.0045	0.0018
PS(14:0/16:1);PS(12:0/18:1)	0.465	0.427	0.0072	0.0073
PS(14:0/18:2);PS(16:1/16:1)	0.308	0.249	0.0300	0.0523

Table S3. Continued

Lipid Common Name	*ATPsynδ* Kd (#7018)	*ATPsynδ* Kd (#7019)	*ATPsynδ* Kd (#7018)	*ATPsynδ* Kd (#7019)
	log2 fold change		p-value	
PS(18:3/18:3)	-0.242	-0.440	0.2456	0.0233
DG(12:0/12:0/0:0)	0.604	0.173	0.0280	0.5600
DG(15:0/18:1/0:0);DG(16:1/17:0/0:0)	-0.542	-0.776	0.2021	0.0474
DG(16:0/18:1/0:0)	-0.760	-0.649	0.0180	0.0261
DG(16:0/18:2)	-0.777	-0.725	0.0074	0.0070
DG(16:1/18:1/0:0)	-0.711	-0.422	0.0022	0.0241
DG(16:1/18:2/0:0)	-0.606	-0.823	0.0803	0.0150
DG(17:0/18:1/0:0)	-0.673	-0.692	0.0226	0.0132
DG(18:0/18:1/0:0)	-0.430	-0.306	0.0035	0.0147
DG(18:1/0:0/18:1)	-0.670	-0.332	0.0004	0.0172
DG(18:1/18:2/0:0)	-0.362	-0.546	0.0055	0.0002
DG(18:2/0:0/18:2);DG(18:1/18:3/0:0)	-0.119	-0.568	0.2911	0.0001
TG(34:0)	0.919	1.154	0.0045	0.0007
TG(36:0)	0.789	0.650	0.0005	0.0011
TG(37:0)	0.563	0.527	0.0178	0.0166
TG(38:1)_A	0.574	0.636	0.0047	0.0016
TG(38:1)_B	0.601	0.749	0.0028	0.0004
TG(39:1)	0.188	0.367	0.3051	0.0275
TG(40:1)	0.368	0.426	0.0382	0.0130
TG(40:2)	0.261	0.658	0.2482	0.0043
TG(42:2)	0.206	0.386	0.1345	0.0062
TG(44:3)	0.055	0.465	0.8342	0.0027
TG(49:3)	-0.393	-0.161	0.0008	0.0583

Lipid common name annotation ZZ(X1:Y1/X2:Y2) where ZZ = lipid class; X1 = number of carbons in chain 1; Y1 = number of double bonds in chain 1; X2 = number of carbons in chain 2; Y1 = number of double bonds in chain 2. Identifications with ZZ(X:Y) denotes the total number of carbons (X) and double bonds (Y) in the chains. CE = cholesterol ester; Cer = ceramide; PE-Cer = phosphoethanolamine ceramide; CL = cardiolipin; PC = glycerophosphocholine; PCO and PCP = alkyl and alkenyl glycerophosphocholine, respectively; PE = glycerophosphoethanolamine; PEO and PEP = alkyl and alkenyl glycerophosphoethanolamine, respectively; PG = glycerophosphoglycerol; PI = glycerophosphoinositol; PS = glycerophosphoserine; DG = diacylglycerol; TG = triacylglycerol. Common name with '_A' and '_B' indicates the lipids are structural isomers of each other. Log2 fold change coloring scales from +1 (red) to -1 (blue). Red font p-values indicates p-value less than 0.05.

Control and 2 mutant female fly lines were analyzed. The control is a *tub-Gal80[ts] (*Bloomington # *7019)/UAS-ATPsynδ RNAi* and the mutants are *tub-Gal80[ts] (*Bloomington # *7019)/UAS-ATPsynδ RNAi; da-Gal4* (ubiquitous driver)/+ and *tub-Gal80[ts] (*Bloomington # *7018)/UAS-ATPsynδ RNAi; da-Gal4* (ubiquitous driver)/+. For conditional knock-down of *ATPsynδ RNAi* using *tubP-GAL80^{ts},* flies were reared at room temperature (21°C) during development, and the adult flies were kept for three days at 28°C before metabolomics analysis. Prior to extracting the lipids as in table s2, 0.15 mm zirconia oxide beads and 0.3 mL of methanol were added to tubes containing female and male flies (n=15 and 3 replicates each) and placed in -80 ºC pre-chilled Eppendorf Safe-Lock tube holder. Samples were homogenized using a Bullet Blender (BB50-DX) for 3 mins at speed 10. The homogenized samples were centrifuged at 8000 *x g* for 10 min at 4 ºC and the lysate was transferred into to 2.0 mL Sorenson low-binding microcentrifuge tubes. An addition 100 µl of methanol, 800 µl chloroform, and 300 µl of water was then added to the lysate and processed as the plasma samples outlined above. Lipids were analyzed as outlined above with the plasma samples. The algorithm RMD-PAV and Pearson correlation were used to identify any outlier biological samples, with none removed.[10] Molecules with inadequate data for either qualitative or quantitative statistical tests were also removed from the datasets prior to normalization via global median centering.[11] ANOVA with a Dunnett test correction and a Bonferroni-corrected g-test was used to compare each mutant to the control.

Supplemental References

1. Smet, J., Seneca, S., de Paepe, B., Meulemans, A., Verhelst, H., Leroy, J., De Meirleir, L., Lissens, W., Van Coster, R. (2009). Subcomplexes of mitochondrial complex V reveal mutations in mitochondrial DNA. *Electrophoresis* 30, 3565-3572.

2. Dietzl, G., Chen, D., Schnorrer, F., Su, K.-C., Barinova, Y., Fellner, M., Gasser, B., Kinsey, K., Oppel, S., Scheiblauer, S., et al. (2007). A genome-wide transgenic RNAi library for conditional gene inactivation in Drosophila. *Nature* 448, 151–156.

3. Bischof, J., Maeda, R.K., Hediger, M., Karch, F., and Basler, K. (2007). An optimized transgenesis system for Drosophila using germ-line-specific phiC31 integrases. *Proc. Natl. Acad. Sci. U. S. A*. 104, 3312–3317.

4. Jaiswal, M., Haelterman, N.A., Sandoval, H., Xiong, B., Donti, T., Kalsotra, A., Yamamoto, S., Cooper, T.A., Graham, B.H., and Bellen, H.J. (2015). Impaired mitochondrial energy production causes light-induced photoreceptor degeneration independent of oxidative stress. *PLoS Biol.* 13 (7): e1002197.

5. Chennamsetty, I., Coronado, M., Contrepois, K., Keller, M.P., Carcamo-Orive, I., Sandin, J., Fajardo, G., Whittle, A.J., Fathzadeh, M., Snyder, M., et al. (2016). Nat1 Deficiency Is Associated with Mitochondrial Dysfunction and Exercise Intolerance in Mice. *Cell Rep* 17, 527–540.

6. Contrepois, K., Jiang, L., and Snyder, M. (2015). Optimized Analytical Procedures for the Untargeted Metabolomic Profiling of Human Urine and Plasma by Combining Hydrophilic Interaction (HILIC) and Reverse-Phase Liquid Chromatography (RPLC)–Mass Spectrometry. *Mol. Cell. Proteomics* 14, 1684–1695.

7. Nakayasu, E.S., Nicora, C.D., Sims, A.C., Burnum-Johnson, K.E., Kim, Y.-M., Kyle, J.E., Matzke, M.M., Shukla, A.K., Chu, R.K., Schepmoes, A.A., et al. (2016). MPLEx: a Robust and Universal Protocol for Single-Sample Integrative Proteomic, Metabolomic, and Lipidomic Analyses. *mSystems* 1, e00043-16.

8. Kyle, J.E., Crowell, K.L., Casey, C.P., Fujimoto, G.M., Kim, S., Dautel, S.E., Smith, R.D., Payne, S.H., and Metz, T.O. (2017). LIQUID: An-open source software for identifying lipids in LC-MS/MS-based lipidomics data. *Bioinformatics* 33, 1744–1746.

9. Pluskal, T., Castillo, S., Villar-Briones, A., and Orešič, M. (2010) MZmine 2: Modular framework for processing, visualizing, and analyzing mass spectrometry- based molecular profile data. *BMC Bioinformatics* 11:395.

10. Matzke, M.M., Waters, K.M., Metz, T.O., Jacobs, J.M., Sims, A.C., Baric, R.S., Pounds, J.G., and Webb-Robertson, B.-J.M. (2011). Improved quality control processing of peptide-centric LC-MS proteomics data. *Bioinformatics* 27 (20) 2866–2872.

11. Webb-Robertson, B.-J.M., Mccue, L.A., Waters, K.M., Matzke, M.M., Jacobs, J.M., Metz, T.O., Varnum, S.M., and Pounds, J.G. (2010) Combined Statistical Analyses of Peptide Intensities and Peptide Occurrences Improves Identification of Significant Peptides from MS-Based Proteomics Data. *J Proteome Res.* 9(11):5748-56.
