# Supplementary material for: Biallelic Mutations in ATP5F1D, which Encodes a Subunit of ATP Synthase, Cause a Metabolic Disorder
Source: Am J Hum Genet. 2018 Feb 22;102(3):494–504. doi: 10.1016/j.ajhg.2018.01.020 (PMC6117612; doi:10.1016/j.ajhg.2018.01.020)
Supplement: Document S2. Article plus Supplemental Data [file mmc2.pdf]

REPORT

Biallelic Mutations in *ATP5F1D*, which Encodes a Subunit of ATP Synthase, Cause a Metabolic Disorder

Monika Oláhová,[1,24] Wan Hee Yoon,[2,3,23,24] Kyle Thompson,[1,24] Sharayu Jangam,[3] Liliana Fernandez,[4] Jean M. Davidson,[4] Jennifer E. Kyle,[5] Megan E. Grove,[6] Dianna G. Fisk,[6] Jennefer N. Kohler,[4] Matthew Holmes,[1] Annika M. Dries,[4] Yong Huang,[4] Chunli Zhao,[4] Kévin Contrepois,[7] Zachary Zappala,[7] Laure Frésard,[8] Daryl Waggott,[4] Erika M. Zink,[5] Young-Mo Kim,[5] Heino M. Heyman,[5] Kelly G. Stratton,[9] Bobbie-Jo M. Webb-Robertson,[9] Undiagnosed Diseases Network, Michael Snyder,[7] Jason D. Merker,[6,8] Stephen B. Montgomery,[7,8] Paul G. Fisher,[4] René G. Feichtinger,[10] Johannes A. Mayr,[10] Julie Hall,[11] Ines A. Barbosa,[12] Michael A. Simpson,[12] Charu Deshpande,[13] Katrina M. Waters,[5] David M. Koeller,[14] Thomas O. Metz,[5] Andrew A. Morris,[15,16] Susan Schelley,[17] Tina Cowan,[8] Marisa W. Friederich,[18] Robert McFarland,[1] Johan L.K. Van Hove,[18] Gregory M. Enns,[17] Shinya Yamamoto,[3,19,20,21] Euan A. Ashley,[4,6,7,22] Michael F. Wangler,[3,20] Robert W. Taylor,[1] Hugo J. Bellen,[2,3,19,20,21] Jonathan A. Bernstein,[4,17] and Matthew T. Wheeler[4,22,25,*]

ATP synthase, H^+ transporting, mitochondrial F1 complex, δ subunit (ATP5F1D; formerly ATP5D) is a subunit of mitochondrial ATP synthase and plays an important role in coupling proton translocation and ATP production. Here, we describe two individuals, each with homozygous missense variants in *ATP5F1D*, who presented with episodic lethargy, metabolic acidosis, 3-methylglutaconic aciduria, and hyperammonemia. Subject 1, homozygous for c.245C>T (p.Pro82Leu), presented with recurrent metabolic decompensation starting in the neonatal period, and subject 2, homozygous for c.317T>G (p.Val106Gly), presented with acute encephalopathy in childhood. Cultured skin fibroblasts from these individuals exhibited impaired assembly of F_1F_O ATP synthase and subsequent reduced complex V activity. Cells from subject 1 also exhibited a significant decrease in mitochondrial cristae. Knockdown of *Drosophila ATPsynδ*, the *ATP5F1D* homolog, in developing eyes and brains caused a near complete loss of the fly head, a phenotype that was fully rescued by wild-type human *ATP5F1D.* In contrast, expression of the *ATP5F1D* c.245C>T and c.317T>G variants rescued the head-size phenotype but recapitulated the eye and antennae defects seen in other genetic models of mitochondrial oxidative phosphorylation deficiency. Our data establish c.245C>T (p.Pro82Leu) and c.317T>G (p.Val106Gly) in *ATP5F1D* as pathogenic variants leading to a Mendelian mitochondrial disease featuring episodic metabolic decompensation.

Mitochondrial diseases are clinically and genetically heterogeneous. Findings such as hyperammonemia, lactic acidosis, and rhabdomyolysis suggest mitochondrial dysfunction and can occur as a result of defects in fatty acid oxidation as well as disorders of the respiratory chain. Defects in the electron transport chain (ETC), which underlies oxidative phosphorylation (OXPHOS), can be caused by mutations in the nuclear or mitochondrial genome.[1,2] Accordingly, inheritance can be autosomal, sex linked, or maternal. Presentations vary widely and range from lethal neonatal metabolic decompensation to chronic progressive disorders of adulthood.

Complex V is the final multi-subunit complex of the OXPHOS system. It harnesses energy from the proton electrochemical gradient to synthesize ATP from ADP[3] and inorganic phosphate, which is the main source of energy for intracellular metabolic pathways.[4] Mitochondrial ATP synthase consists of two main functional domains, the soluble F_1 catalytic portion in the mitochondrial matrix and the inner-membrane-embedded F_O, which allows protons

[1]Wellcome Centre for Mitochondrial Research, Institute of Neuroscience, Newcastle University, Newcastle upon Tyne NE2 4HH, UK; [2]Howard Hughes Medical Institute, Baylor College of Medicine, Houston, TX 77030, USA; [3]Department of Molecular and Human Genetics, Baylor College of Medicine, Houston, TX 77030, USA; [4]Center for Undiagnosed Diseases, Stanford University, Stanford, CA 94305, USA; [5]Biological Sciences Division, Earth and Biological Sciences Directorate, Pacific Northwest National Laboratory, Richland, WA 99352, USA; [6]Clinical Genomics Program, Stanford Health Care, Stanford, CA 94305, USA; [7]Department of Genetics, Stanford University School of Medicine, Stanford, CA 94305, USA; [8]Department of Pathology, Stanford University, Stanford, CA 94305, USA; [9]Computing & Analytics Division, National Security Directorate, Pacific Northwest National Laboratory, Richland, WA 99352, USA; [10]Department of Pediatrics, Paracelsus Medical University, 5020 Salzburg, Austria; [11]Department of Neuroradiology, Royal Victoria Infirmary, Newcastle upon Tyne NE1 4LP, UK; [12]Department of Medical and Molecular Genetics, King's College London School of Basic and Medical Biosciences, London SE1 9RT, UK; [13]Clinical Genetics Unit, Guys and St. Thomas' NHS Foundation Trust, London SE1 9RT, UK; [14]Department of Molecular & Medical Genetics, Oregon Health & Science University, Portland, OR 97239, USA; [15]Institute of Human Development, University of Manchester, Manchester M13 9PL, UK; [16]Willink Metabolic Unit, Genomic Medicine, Saint Mary's Hospital, Manchester University NHS Foundation Trust, Manchester M13 9WL, UK; [17]Department of Pediatrics, Stanford University School of Medicine, Stanford, CA 94305, USA; [18]Clinical Genetics and Metabolism, Department of Pediatrics, University of Colorado at Denver, Aurora, CO 80045, USA; [19]Program in Developmental Biology, Baylor College of Medicine, Houston, TX 77030, USA; [20]Jan and Duncan Neurological Research Institute, Texas Children's Hospital, Houston, TX 77030, USA; [21]Department of Neuroscience, Baylor College of Medicine, Houston, TX 77030, USA; [22]Department of Medicine, Stanford University School of Medicine, Stanford, CA 94305, USA
[23]Present address: Functional & Chemical Genomics Research Program, Oklahoma Medical Research Foundation, Oklahoma City, OK 73104, USA
[24]These authors contributed equally to this work
[25]Twitter: @MWheelerMD
*Correspondence: wheelerm@stanford.edu
https://doi.org/10.1016/j.ajhg.2018.01.020.
© 2018 The Authors. This is an open access article under the CC BY license (http://creativecommons.org/licenses/by/4.0/).

to pass from the intermembrane space to the matrix (reviewed by Jonckheere et al.[5]). Two subunits of the F_O (a and A6L) are encoded by mtDNA (*MT-ATP6* and *MT-ATP8*), whereas the other subunits and accessory factors are encoded by the nuclear genome. Although mitochondrial disorders due to defects in mitochondrial complex V have been reported, they are very rare in comparison with those due to mutations in the genes encoding the proteins of the other complexes (I–IV).[6,7]

We report the clinical and genetic findings of two children with suspected mitochondrial disease from unrelated families. Subject 1 is the only child of first-cousin Mexican-American parents. On the second day of life, she presented with lethargy and severe anion-gap acidosis. Initial laboratory investigations showed hypoglycemia (28 mg/dL [normal 45–100]), lactic acidosis (34 mmol/L [normal < 2.1]), and hyperammonemia (359 μmol/L [normal < 30]). Initial management included intravenous fluids with dextrose and intravenous lipid administration. Within 24 hr, lactic acid and ammonia had decreased to 4.8 mmol/L and 70 μmol/L, respectively. Ammonia-scavenging medications were not administered. Qualitative organic acid studies showed moderate to marked elevation of lactic, fumaric, malic, p-hydroxyphenyllactic, and 3-methylglutaconic acids. An acylcarnitine profile showed nonspecific elevations of numerous short-, medium-, and long-chain acylcarnitine species. Creatine kinase was not assessed during her initial presentation. Brain MRI with magnetic resonance spectroscopy was normal. Her most recent evaluation was at 9 years of age. 3-methylglutaconic aciduria has been a persistent finding in urine organic acid analysis. She has mild developmental delays and short stature. Between the ages of 1 and 4 years, she was noted to have dilated cardiomyopathy and subsequent normalization of resting systolic function. Ophthalmologic examination at 8 years of age showed a prominent macular reflex. No other findings were noted. Neurologic examination at 9 years of age showed mild proximal weakness (4/5) greater than distal weakness (5/5) in her extremities. She additionally had gait imbalance and ankle contractures with reduced reflexes (1+). Cranial nerve examination showed slightly decreased strength with eye closure. Cerebellar examination and sensation were normal. She has had at least nine episodes of metabolic decompensation manifesting with lactic acidosis and muscle breakdown, which required hospital admission. During decompensation, serum creatine kinase has been repeatedly elevated to greater than 500 U/L and as high as 1,109 U/L. These episodes have been responsive to intravenous fluids with dextrose. Severe hyperammonemia has not recurred since the newborn period. She has been treated with oral supplements including alpha-lipoic acid, ubiquinone, riboflavin, thiamine, biotin, pantothenic acid, and ascorbic acid and has experienced subjective improvement in her physical stamina.

Subject 2 is the first child born to healthy first-cousin UK Asian parents, and he has a healthy younger brother. He was born at term by vacuum-assisted delivery after an uneventful pregnancy. There were no perinatal problems. His speech was delayed, and he received speech therapy, but he otherwise met typical developmental milestones. At age 4 years and 10 months, he presented with an encephalopathic illness after 24 hr of coryza and fever. He was witnessed to have a progressive deterioration in the level of consciousness over several hours and had a brief tonic-clonic seizure, which was managed with phenobarbital. Ultimately, he required intubation and mechanical ventilation, which was maintained for 2 days. He had ketoacidosis and hyperammonemia (maximum 262 μmol/L [normal < 30]). Plasma lactate was 5.3 mmol/L (normal < 2.1) at presentation but decreased to 2.1 mmol/L within 5 hr and subsequently to 1.1 mmol/L, at which stage the cerebrospinal fluid lactate was 1.8 mmol/L (normal < 2.5). Initial treatment included intravenous fluids with dextrose, intravenous carnitine (100 mg/kg/day), and sodium benzoate (250 mg/kg/day). The ammonia level normalized within 24 hr. Neuroimaging showed diffuse swelling of the cerebral cortex bilaterally, especially in the temporal lobes, as well as lesser changes in the cerebellar hemispheres (Figure S1). There was swelling and signal change in the subcortical and deep white matter, although the periventricular white matter was spared. There were also signal changes in the thalami, midbrain, pons, corpus callosum, and basal ganglia. MRI 1 year later showed resolution of these abnormal findings. The transient nature of the MRI findings was interpreted as evidence that they might have reflected the presence of edema that resolved over time. After this episode, he made a full recovery to his prior baseline. He attends a regular school, and at 6 years of age he had a full-scale IQ of 81 (Wechsler Preschool and Primary Scale of Intelligence[8]) and poor attention (as assessed by a score of 51 [first percentile] on the Attention & Concentration Index of the Children's Memory Scale[9]). He now has mildly impaired exercise tolerance, tires easily, and uses a wheelchair for long distances. Neurologic examination after his initial presentation noted mild hypotonia, but this has since resolved. He has pes planus, pes adductus, and dyspraxia of gait but no other abnormalities on detailed neurologic examination. The cranial nerve, motor, sensory, and cerebellar examinations have otherwise been normal. On recent routine evaluation, 12-lead electrocardiography and echocardiography were normal. Organic acid analysis has persistently shown a mild increase in 3-methylglutaconic and 3-methylglutaric acid excretion. He has been a fussy eater since infancy and receives much of his nutrition as liquid formula. He periodically develops lethargy and emesis typically in association with febrile illness. Symptoms are improved by oral dextrose containing fluids. He experiences emesis approximately twice a week and has frequent stomach aches. He has a history of intermittent squint and has developed amblyopia of the left eye, despite patching of the right eye. There are no other ophthalmological abnormalities. His linear growth has been typical for his age, and physical examination shows no significant findings. The

parents and younger sibling (currently 4 years of age) are in good health.

Informed consent for diagnostic and research studies was obtained for both subjects in accordance with the Declaration of Helsinki protocols and approved by the central institutional review board (IRB) at the NIH National Human Genome Research Institute for the Undiagnosed Diseases Network (subject 1) and by the local IRB in Newcastle upon Tyne, UK (subject 2).

Initial diagnostic analyses of cultured skin fibroblasts for pyruvate carboxylase, pyruvate dehydrogenase, and enzyme activities of respiratory chain complexes I–IV in subject 1 were normal. Complex V was not assessed during these studies. Subsequent blue-native PAGE (BN-PAGE) with in-gel activity staining showed qualitatively decreased activity of complex V (Figure S2). For subject 2, complexes I–IV of the mitochondrial respiratory chain were all within normal ranges in muscle, as were routine histology and histochemistry. Pyruvate dehydrogenase activity was normal in cultured skin fibroblasts. Subsequent analysis of the activity of respiratory chain complexes in fibroblasts from each affected individual showed a marked decrease in complex V enzymatic activity (Table 1).

Analysis of mtDNA from blood in both affected individuals showed no mtDNA rearrangements or point mutations, and the mtDNA copy number was normal. Whole-exome sequencing (WES) was performed according to previously described methodologies and filtering pipelines.[10–13] In subject 1, exome sequencing was performed with VCRome 2.1 in-solution exome probes, as well as additional probes for over 2,600 Mendelian-disease-related genes. Library DNA was sequenced on an Illumina HiSeq for 100 bp paired-end reads. Data analysis was performed with Mercury 1.0 and was followed by reanalysis using phenotype- and inheritance-model-based filters with Ingenuity Variant Analysis (QIAGEN) and a curated list of mitochondrial expressed genes. Variants were confirmed by Sanger sequencing of DNA samples from the affected subject and parents. In subject 2, exome sequencing was performed in the family trio with Agilent SureSelectXT All Exon V5 on a HiSeq 2500 with 100 bp paired-end reads. Variant calls were generated with an in-house pipeline as previously described with minor alterations.[10] Variant files were annotated with respect to genes and variant functional consequences with the ANNOVAR tool. Further annotation included information on variant novelty and estimated population frequencies from cross-referencing identified variants with publicly available data and >1,000 control exomes processed with a Novoalign-based pipeline.

In both subjects, WES identified biallelic variants in *ATP5F1D* (formerly *ATP5D* [MIM: 603150; GenBank: NM_001687.4]), which encodes the F_1 δ subunit of complex V.[14] *ATP5F1D* is located at 19p13.3 (1,241,750–1,244,825 [GRCh38.p7]). The predominant transcript consists of four exons encoding a 146 amino acid mature protein with a 22 amino acid presequence.[14] Research reanalysis of proband-only clinical WES data from subject 1 identified a homozygous c.245C>T (p.Pro82Leu) variant in *ATP5F1D*. Sanger sequencing confirmed bi-parental inheritance of the c.245C>T variant (Figure 1A). There was no detectable abnormality in the abundance or splicing of the *ATP5F1D* transcript (Figure S3). In parallel, WES was undertaken in the family trio of subject 2, revealing a homozygous c.317T>G (p.Val106Gly) variant in exon 3 of *ATP5F1D*. Analysis of WES and Sanger confirmation in the parents demonstrated bi-parental inheritance of the c.317T>G (p.Val106Gly) variant (Figure 1A). The identified variants (p.Pro82Leu and p.Val106Gly) affect highly conserved amino acids (Figure 1B). The c.245C>T variant has been observed in 1 of 142,292 total alleles (1 of 23,192 alleles of Latino ethnicity) in the gnomAD dataset and has not been seen in other publicly searchable datasets, whereas c.317T>G had not been observed in any dataset.[16] *In silico* structural modeling indicated that each amino acid variant induces a change in the predicted protein structure (Figure 1C).[15]

Although the two subjects both had features of mitochondrial disease and metabolic decompensation, they differed in that subject 1 presented a few days after birth, had elevated creatine kinase, and had normal brain MRI. Subject 2 was not evaluated for mitochondrial phenotypes until after 4 years of age. Because both had homozygous missense variants in *ATP5F1D* and because no disease annotation for *ATP5F1D* is known, we undertook additional studies in subject cells and in *Drosophila melanogaster* to determine whether these missense changes were pathogenic.

To investigate the functional effects of the identified *ATP5F1D* variants, we performed OXPHOS protein analysis from cultured skin fibroblasts of each affected individual. Immunoblotting of protein extracts from subject fibroblasts showed that steady-state amounts of ATP5F1D were not affected (Figure 2A). However, other complex V subunits (ATP5F1A, ATP5F1B, and ATP5PO) were clearly decreased in abundance (Figure 2A). Double immunofluorescence staining of fibroblasts from subjects 1 and 2 (Figure S4) revealed lower signal of the complex V subunit ATP5F1A than of that in age-matched control cells, confirming abnormality of complex V. The abundance of other OXPHOS complex subunits was not decreased, whereas complex V subunits showed a marked reduction (Figure 2B). This was confirmed by BN-PAGE analysis, which showed a loss of complex V assembly, whereas other complexes were relatively unaffected (Figure 2C). We confirmed these findings in skeletal muscle extracts from subject 2, given that steady-state amounts of CI–CIV subunits and complexes were not affected, whereas the amounts of complex V subunit ATP5F1A (Figure 2D) and fully assembled complex V (Figure 2E) were markedly decreased. These data show that cells from the subjects exhibited reduced amounts of complex V. We posit that the missense changes present in both subjects do not alter the amount of ATP5F1D but instead lead to an inability

Table 1. Genetic, Biochemical, and Clinical Findings in Individuals with Biallelic *ATP5F1D* Variants

		***ATP5F1D* Variants**		**OXPHOS Activities in Cultured Skin Fibroblasts**					**Clinical Presentation**	
**ID**	**Sex**	**cDNA (GenBank: NM_001687.4)**	**Protein (GenBank: NP_001687.1)**	**Respiratory Chain Complex**	**Mean Enzyme Activity (%)**	**Absolute Values**	**Normal Range of Activities**	**Muscle Biopsy**	**Age at Presentation**	**Salient Clinical Features**
S1	female	c.[245C>T];[245C>T]	p.[Pro82Leu];[Pro82Leu]	I	83%	24	18–53	normal histology and respiratory chain enzymes	2 days	hyperammonemia, cardiomyopathy, lactic acidosis, rhabdomyolysis fatigability, short stature
				I + III	267%	310	61–220			
				II	92%	71	54–124			
				II + III	130%	180	79–219			
				IV	44%	162	270–659			
				V	5% (↓↓↓)	7	78–287			
				CS	63%	197	225–459			
S2	male	c.[317T>G];[317T>G]	p.[Val106Gly];[Val106Gly]	I	93%	27	18–53	normal histology and respiratory chain enzymes	4 years and 10 months	hyperammonemia, ketoacidosis, delayed speech
				I + III	151%	174	61–220			
				II	98%	76	54–124			
				II + III	139%	193	79–219			
				IV	142%	519	270–659			
				V	16% (↓↓)	23	78–287			
				CS	101%	314	225–459			

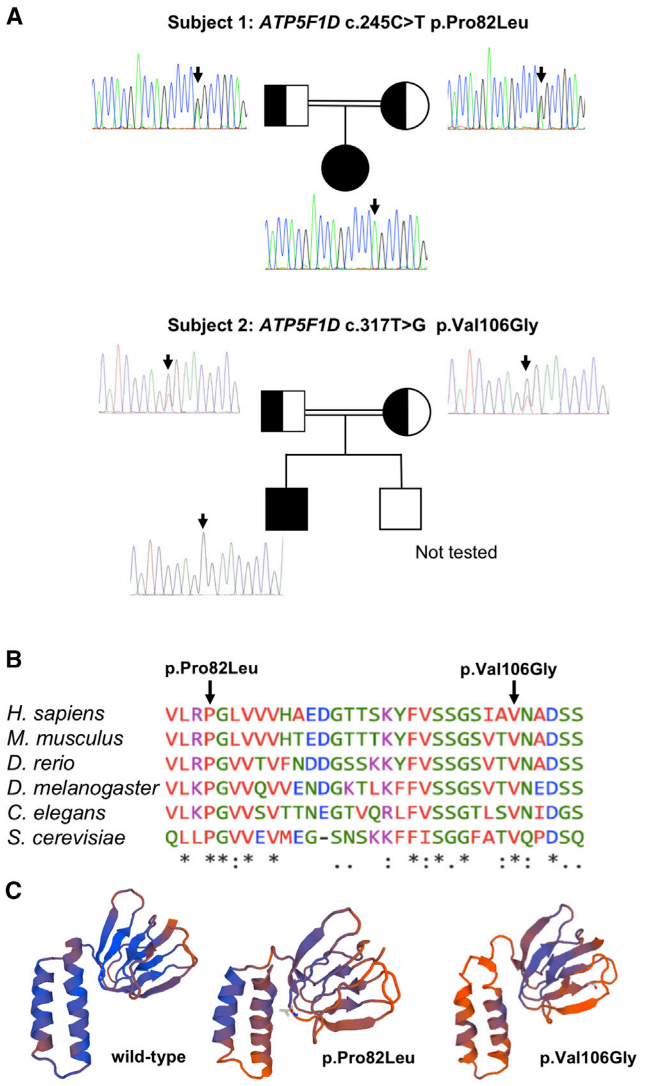

Figure 1. Molecular Genetic Studies of *ATP5F1D* Variants
(A) Pedigrees and sequencing chromatograms of the two affected families show segregation of the homozygous *ATP5F1D* variant c.245C>T (p.Pro82Leu) in subject 1 and c.317T>G (p.Val106Gly) in subject 2.
(B) Multiple-sequence alignment confirms evolutionary conservation of p.Pro82Leu and p.Val106Gly in both human and flies.
(C) SWISS-MODEL-predicted structure of wild-type, p.Pro82Leu, and p.Val106Gly ATP5F1D.[15]

of ATP5F1D to bind other F_1 subunits correctly and thus result in reduced assembly of complex V.

To assess mitochondrial morphology, we performed transmission electron microscopy (TEM) on cultured skin fibroblasts of subject 1 (Figure 3A). The mitochondria in these fibroblasts were not significantly different in size from those in control fibroblasts (Figure 3C). However, they displayed a dramatic decrease in the number of cristae (Figures 3A and 3D). Induced pluripotent stem cells (iPSCs) derived from fibroblasts of subject 1 were differentiated into iPSC-derived cardiomyocytes (Figure S5A). These cardiomyocytes exhibited both smaller mitochondrial size and markedly fewer cristae than control cardiomyocytes (Figures 3B, 3E, and 3F), as well as impaired maximal respiration in response to palmitate supplementation (Figure S5B).

To determine whether the defects seen in complex V in subject cells were indeed due to the missense variants found in *ATP5F1D*, we studied the variants in *Drosophila*. *ATP synthase δ subunit* (*ATPsynδ*), the *Drosophila* homolog of *ATP5F1D*, is highly conserved (identity 48%, similarity 65%, DIOPT score 10/12),[19] and the affected residues (Pro82 and Val106) are also conserved (Figure 1B). We generated transgenic flies harboring a wild-type human cDNA (*UAS-ATP5F1D*^{WT}) as well as both variant cDNAs (*UAS-ATP5F1D*^{P82L} and *UAS-ATP5F1D*^{V106G}). The expression of these cDNAs can be induced by the transcription factor GAL4.[20] To knock down the protein, we ubiquitously expressed a UAS-*ATPsynδ* RNAi by using various ubiquitous *Gal4* drivers, including *tub-Gal4*, *Actin-Gal4*, or *da-Gal4*.[21] All drivers caused lethality (Figure S6C), consistent with previous observations.[22] Pan-neuronal expression of the *ATPsynδ* RNAi with the *elav*$^{[C155]}$*-Gal4* driver resulted in lethality early in development (Figure S6D). This lethality was rescued by expression of human *ATP5F1D*^{WT}, but not by expression of the two human *ATP5F1D* variants (*ATP5F1D*^{P82L} and *ATP5F1D*^{V106G}) (Figure S6D). These data indicate that human *ATP5F1D* is functional in flies and that the two *ATP5F1D* variants (*ATP5F1D*^{P82L} and *ATP5F1D*^{V106G}) are not fully functional.

To further examine the effect of these variants in adult flies, we used the *eyeless (ey)-Gal4* driver,[23] whose expression is restricted to the eye, antenna, and part of the brain. Expression of *ATPsynδ* RNAi in the developing eye, brain, and antenna with the *ey-Gal4* driver caused pupal lethality and a near-complete loss of the head (Figures 4A–4C). This lethality and the development of the eye, antenna, and brain were fully rescued by expression of human *ATP5F1D*^{WT} (Figure 4A). Expression of the two human *ATP5F1D* variants (*ATP5F1D*^{P82L} and *ATP5F1D*^{V106G}) in flies in which the endogenous ATPsynδ had been knocked down by the *eyGal4* driver rescued lethality (Figure 4A). However, the animals rescued by the *eyGal4* driver retained abnormal eye and antennal phenotypes (Figures 4D–4K). Interestingly, rescue with the *ATP5F1D*^{V106G} allele corresponding to subject 2 showed more severe phenotypes than rescue with *ATP5F1D*^{P82L}—the *ATP5F1D*^{V106G} allele only partially rescued lethality, elicited a glossy-eye phenotype less frequently than *ATP5F1D*^{P82} expression, and caused more severe defects in electroretinogram recordings than did the *ATP5F1D*^{P82} allele (Figure S7). Hence, the mutant ATP5F1D proteins are not fully functional when tested in flies, and the function of *ATP5F1D*^{V106G} is more severely affected than *ATP5F1D*^{P82L} in this system.

To evaluate the metabolic effects of these mitochondrial defects, we performed exploratory analyses of untargeted plasma metabolite and lipid profiles in samples from subject 1 and in transgenic flies. Plasma metabolomic

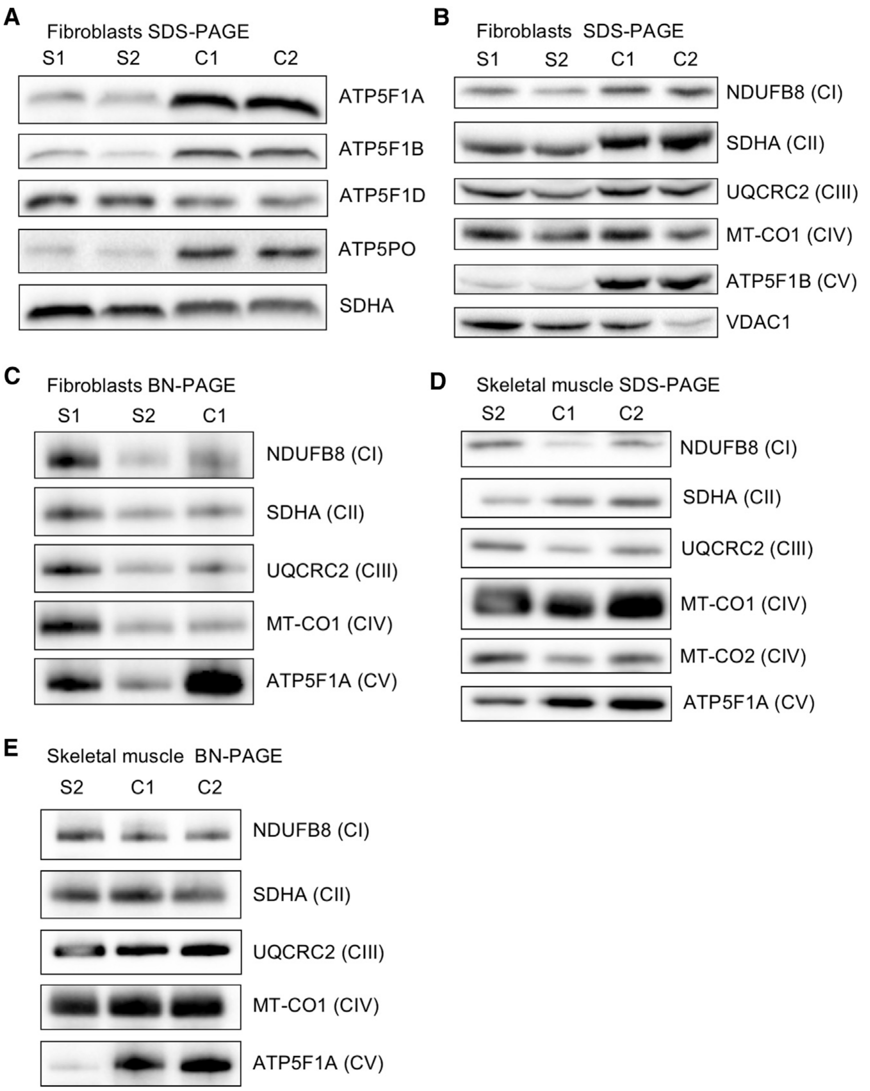

Figure 2. Biallelic Variants in *ATP5F1D* Impair the Steady-State Amounts of the F_1F_O ATP Synthase Complex and Subunits
Immunoblot and BN-PAGE analysis were carried out on subject cultured skin fibroblasts and skeletal muscle samples as previously described.[11,17,18] SDS-PAGE and immunoblot analysis of whole-cell lysates (40 μg) isolated from cultured skin fibroblasts of affected subjects 1 (S1) and 2 (S2) and age-matched control individuals show (A) the steady-state amounts of complex V subunits (ATP5F1A, ATP5F1B, ATP5F1D, and ATP5PO) and (B) the amounts of individual OXPHOS complex subunits. One-dimensional BN-PAGE analysis was performed for assembled OXPHOS complexes in n-dodecyl-β-D-maltoside (DDM; 850520P, Sigma)-solubilized mitochondrial extracts isolated from control, S1, and S2 fibroblasts (C). Steady-state amounts (D) and assembly (E) of OXPHOS complexes and subunits in DDM-solubilized mitochondrial extracts from control and subject 2 skeletal muscle demonstrate a decrease in complex V. In (C) and (E), mitochondrial lysates (100 μg) were loaded on a 4%–16% native gel (Life Technologies), and then protein complexes were immobilized onto polyvinylidene difluoride membranes and subjected to immunoblotting with the indicated OXPHOS-subunit-specific antibodies. In (A)–(E), nuclear-encoded SDHA (ab14715, Abcam) or porin (VDAC1, ab14734, Abcam) was used as a loading control. Abbreviations are as follows: BN, blue native; CI, complex I; CII, complex II; CIII, complex III; CIV, complex IV; and CV, complex V.

profiling[24,25] revealed accumulation of the TCA cycle intermediates malic acid and citric acid, as well as compensatory changes in branched-chain amino acid metabolism (Figure S8 and Table S1). Plasma lipidomic analysis comparing subject 1 samples with those of 136 unrelated control samples revealed increases in long-chain acylcarnitines (C12:1, C14:1, and C16), decreases in dihydroceramides and ceramides, and elevated sphingomyelin, lactosylceramide, and ganglioside (GM3) lipids[26–29] (Figure S9A and Table S2). Similar changes in long-chain acylcarnitines were seen in flies with mildly reduced *ATPsynδ* expression driven by attenuated expression of *ATPsynδ* RNAi (C12 and C14:1), wheras alterations in cardiolipin (CL) profile lipids, highly enriched in mitochondrial inner membranes,[30] (Figure S9B and Table S3) were uniquely observed in fly homogenates. Together, these data suggest that an impairment in mitochondrial fatty acid oxidation might contribute to the hypoglycemia observed in the two subjects.

In summary, we present compelling data that biallelic missense variants in *ATP5F1D* result in a mitochondrial disorder that manifests in childhood with episodic decompensation featuring lactic acidosis and hyperammonemia accompanied by ketoacidosis or hypoglycemia. Chronic manifestations include developmental delay, easy fatiguability, and 3-methylglutaconic aciduria. Interestingly, the two subjects exhibited different ages of onset and differed with respect to the presence of elevated creatine kinase and encephalopathy. Initial clinical studies in both subjects showed normal respiratory chain enzyme profiles (measuring complexes I–IV), and WES was undertaken on account of a clear mitochondrial and/or metabolic phenotype. The pathogenicity of *ATP5F1D* variants (c.245C>T [p.Pro82Leu] and c.317T>G [p.Val106Gly]) identified in these two subjects was confirmed by the segregation of variants with disease in each family (Figure 1), demonstration of severe reduction of complex V activity in subject cultured skin fibroblasts (Figure 2), documentation of fewer mitochondrial cristae in subject cells (Figure 3), and demonstration of incomplete phenotypic rescue by subject *ATP5F1D* variants in *Drosophila* lacking *ATPsynδ* but complete rescue with normal human *ATP5F1D* (Figure 4).

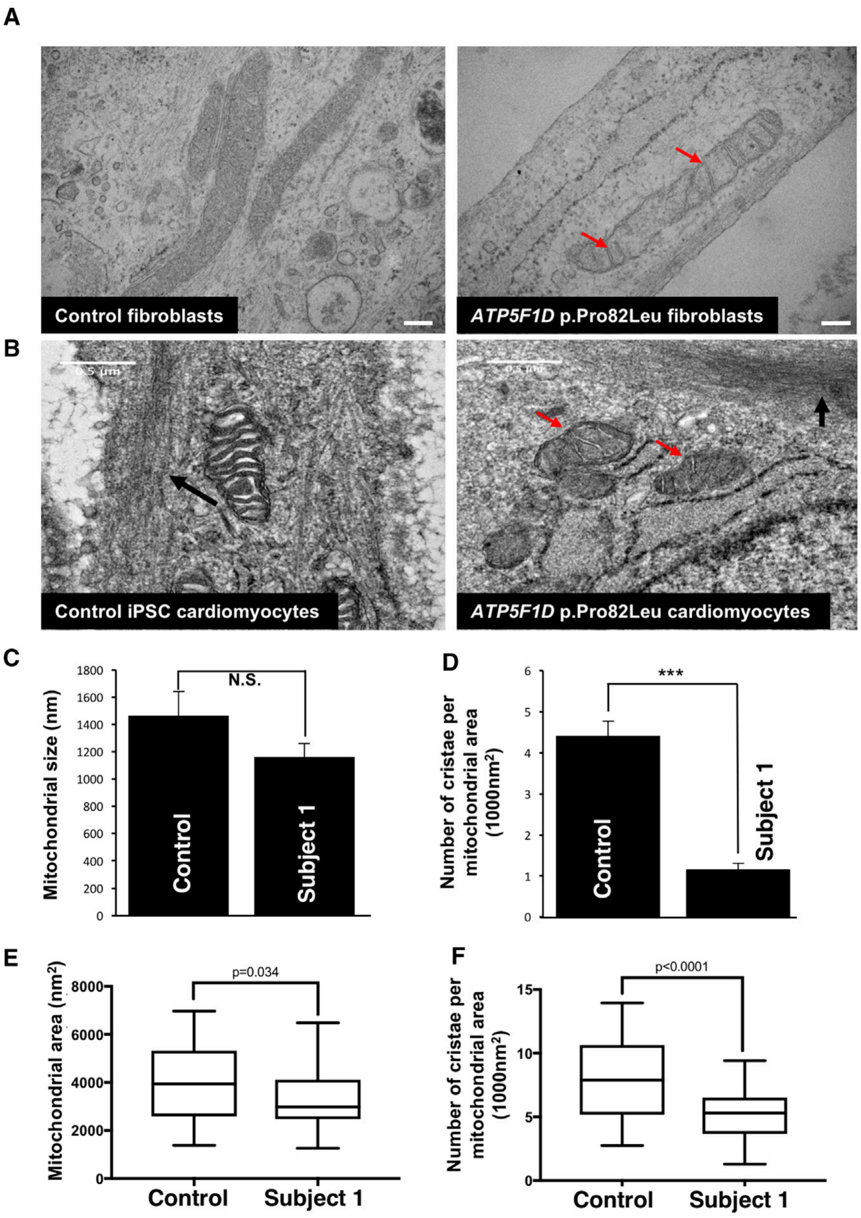

Figure 3. Subject-Derived Cells Carrying a c.245C>T (p.Pro82Leu) *ATP5F1D* Variant Exhibit a Decreased Number of Cristae

(A) TEM of cultured skin fibroblasts from an unaffected control individual and subject 1 (S1) (p.Pro82Leu).

(B) TEM of iPSC-derived cardiomyocytes. Red arrows show mitochondria devoid of cristae in cells from affected individual S1 (p.Pro82Leu). Black arrows indicate nascent sarcomeres. Scale bar: 500 nm.

(C) Quantification of mitochondrial size in control and subject 1 (p.Pro82Leu) fibroblasts. Error bars indicate SEM, and p values were calculated by Student's t test. N.S. indicates not statistically significant.

(D) Quantification of the number of cristae per mitochondrion in control and subject 1 (p.Pro82Leu) fibroblasts. Error bars indicate SEM, and p values were calculated by Student's t test (***$p < 0.001$).

(E) Quantification of the mitochondrial area in control and subject 1 (p.Pro82Leu) iPSC-derived cardiomyocytes. Quartiles and minimum and maximum values are shown, and p values were calculated by an unpaired two-tailed t test ($p = 0.03$).

(F) Quantification of the number of cristae per mitochondrion in control and subject 1 (p.Pro82Leu) iPSC-derived cardiomyocytes. Quartiles and minimum and maximum values are shown, and p values were calculated by an unpaired t test ($p < 0.001$).

Loss of cristae in mitochondria is consistent with phenotypes associated with other complex V mitochondrial mutants. Indeed, ATP synthase forms dimers and oligomers within the mitochondrial inner membrane, and these oligomers have been shown to be important for cristae formation.[31,32] Furthermore, individuals with mutations in *MT-ATP6* (MIM: 516060) have disrupted cristae,[33] and loss of *ATPsynε* (the homolog of human *ATP5F1E*) or *ATPsynγ* (the homolog of human *ATP5F1C*) in flies causes a decreased number of cristae.[22,34] The glossy-eye phenotype provides an additional link between our observation and OXPHOS genes. Indeed, loss of the NADH dehydrogenase (ubiquinone) PDSW subunit (Pdsw) and cytochrome *c* oxidase subunit of Va (*CoVa*) in the fly eye causes glossy eyes.[35] These glossy eyes can be considered a "phenolog" or a non-obvious phenotypic link to mitochondrial disease in humans.[36]

Complex V deficiencies have been reported to be due to mutations in the mtDNA-encoded *MT-ATP6*[33,37] and *MT-ATP8* (MIM: 516070),[38–40] as well as the nuclear-encoded *ATPAF2* (*ATP12* [MIM: 608918])[41,42] and the F_1 subunits *ATP5F1E* (*ATP5E* [MIM: 606153])[43] and *ATP5F1A* (*ATP5A1* [MIM: 164360]).[44,45] The most common nuclear genetic cause of complex V deficiency, however, is associated with *TMEM70* (MIM: 612418),[46] which encodes a protein required for the biogenesis and stability of complex V.[47] The presentation of disorders of complex V has often been described as an early-onset encephalocardiomyopathy that is typically observed in individuals with *TMEM70* mutations.[46,48,49] However, there can be significant clinical heterogeneity associated with different variants in the same gene: for example, mutations in *MT-ATP6* lead to a variety of clinical syndromes, including neurogenic muscle weakness, ataxia, and retinitis pigmentosa (MIM: 551500), Leigh syndrome (MIM: 256000), mitochondrial infantile bilateral striatal necrosis (MIM: 500003), and Charcot-Marie-Tooth hereditary neuropathy.[50,51] The findings of hyperammonemia and increased 3-methylglutaconic aciduria in both subjects during acute episodes of metabolic decompensation

provides an important phenotypic link to complex V deficiencies because these are also prominent in individuals with *TMEM70*,[46,52] *ATP5F1E*,[43] and *ATPAF2*[41,42] mutations. Proper management of hyperammonemic metabolic crises early in life appears to be vital for improving the prognosis of individuals with *TMEM70* mutations.[46,52] Persistent 3-methylglutaconic aciduria is also observed in other complex V deficiency syndromes but is additionally seen in a broader range of metabolic disorders.[53] In summary, the shared and divergent phenotypes observed in our two subjects and the observation that the two variants are both deleterious but to different degrees when tested in *Drosophila* argue for these biallelic mutations in *ATP5F1D* as pathogenic for disease in both subjects.

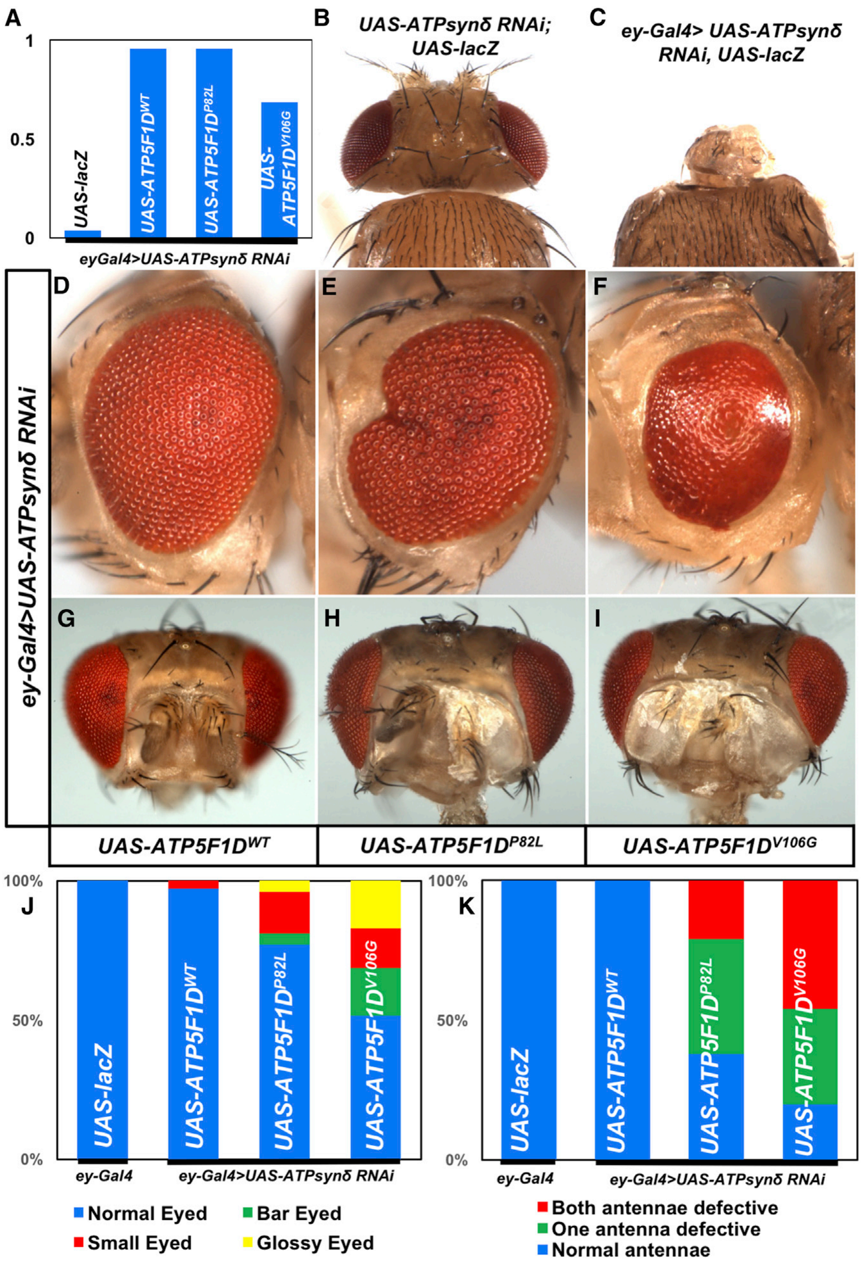

Figure 4. ATP5F1D p.Pro82Leu and p.Val106Gly Are Partial Loss-of-Function Variants

(A) The observed/expected ratio of flies shows the rescue of lethality by the human genes including both variants in the *Drosophila* null background.

(B and C) Expression of *ATPsynδ* RNAi by *ey-Gal4* caused pupal lethality and an extremely reduced head size (*ey-Gal4/UAS-ATPsynδ RNAi; UAS-LacZ/+*) (C), whereas control animals without the *ey-Gal4* driver (*UAS-ATPsynδ RNAi/+; UAS-LacZ*) showed normal head development (B).

(D–F) Light micrographs of fly eyes expressing *ey-Gal4* and *ATPsynδ* RNAi together with *UAS-ATP5F1D^{WT}* (D), *UAS-ATP5F1D^{P82L}* (E), or *UAS-ATP5F1D^{V106G}* (F). We found that expression of *ATP5F1D^{WT}* rescued the tiny-head phenotype caused by knockdown of *ATPsynδ* (D). However, a portion of adult flies expressing *ATPsynδ* RNAi together with *ATP5F1D^{P82L}* or *ATP5F1D^{V106G}* exhibited abnormal eye morphology, including glassy eyes, small eyes, and bar eyes (E and F). Quantification of the phenotypes shows that expression of *ATP5F1D^{V106G}* causes more severe defects than *ATP5F1D^{P82L}* (J).

(G–I) Light micrographs of fly antenna expressing *ey-Gal4* and *ATPsynδ* RNAi together with *UAS-ATP5F1D^{WT}* (G), *UAS-ATP5F1D^{P82L}* (H), or *UAS-ATP5F1D^{V106G}* (I).

(K) Quantification of the antenna morphology phenotypes described in (G)–(I).

We anticipate that additional cases of the *ATP5F1D*-related mitochondrial disorder will be identified, providing us with the opportunity to better define the clinical spectrum of the condition. Given the dramatic phenotype associated with the severe loss of *ATP5F1D* function in a model organism (Figure 4),[22] it is possible that other variants associated with varying phenotypes will also be discovered. At present, the defining features appear to be mild developmental disability, easy fatigability, and episodic biochemical decompensation with acute illness, which can be profound at initial presentation.

Accession Numbers

Whole-exome sequencing data from subject 1 has been deposited in dbGaP per the NIH study protocol and subject consent under accession number dbGaP: phs001232.v1.p1. Details of the pathogenic variants in subjects 1 and 2 have been deposited in ClinVar under accession numbers ClinVar: SCV000453296 and SCV000680464. Data and subject-derived biospecimens are available from the corresponding author.

Supplemental Data

Supplemental Data include nine figures and three tables and can be found with this article online at https://doi.org/10.1016/j.ajhg.2018.01.020.

Consortia

The Undiagnosed Diseases Network co-investigators are David R. Adams, Mercedes E. Alejandro, Patrick Allard, Mahshid S. Azamian, Carlos A. Bacino, Ashok Balasubramanyam, Hayk Barseghyan, Gabriel F. Batzli, Alan H. Beggs, Babak Behnam, Anna Bican, David P. Bick, Camille L. Birch, Devon Bonner, Braden E. Boone, Bret L. Bostwick, Lauren C. Briere, Donna M. Brown, Matthew Brush, Elizabeth A. Burke, Lindsay C. Burrage, Shan Chen, Gary D. Clark, Terra R. Coakley, Joy D. Cogan, Cynthia M. Cooper, Heidi Cope, William J. Craigen, Precilla D'Souza, Mariska Davids, Jyoti G. Dayal, Esteban C. Dell'Angelica, Shweta U. Dhar, Ani Dillon, Katrina M. Dipple, Laurel A. Donnell-Fink, Naghmeh Dorrani, Daniel C. Dorset, Emilie D. Douine, David D. Draper, David J. Eckstein, Lisa T. Emrick, Christine M. Eng, Ascia Eskin, Cecilia Esteves, Tyra Estwick, Carlos Ferreira, Brent L. Fogel, Noah D. Friedman, William A. Gahl, Emily Glanton, Rena A. Godfrey, David B. Goldstein, Sarah E. Gould, Jean-Philippe F. Gourdine, Catherine A. Groden, Andrea L. Gropman, Melissa Haendel, Rizwan Hamid, Neil A. Hanchard, Lori H. Handley, Matthew R. Herzog, Ingrid A. Holm, Jason Hom, Ellen M. Howerton, Yong Huang, Howard J. Jacob, Mahim Jain, Yong-hui Jiang, Jean M. Johnston, Angela L. Jones, Isaac S. Kohane, Donna M. Krasnewich, Elizabeth L. Krieg, Joel B. Krier, Seema R. Lalani, C. Christopher Lau, Jozef Lazar, Brendan H. Lee, Hane Lee, Shawn E. Levy, Richard A. Lewis, Sharyn A. Lincoln, Allen Lipson, Sandra K. Loo, Joseph Loscalzo, Richard L. Maas, Ellen F. Macnamara, Calum A. MacRae, Valerie V. Maduro, Marta M. Majcherska, May Christine V. Malicdan, Laura A. Mamounas, Teri A. Manolio, Thomas C. Markello, Ronit Marom, Julian A. Martínez-Agosto, Shruti Marwaha, Thomas May, Allyn McConkie-Rosell, Colleen E. McCormack, Alexa T. McCray, Matthew Might, Paolo M. Moretti, Marie Morimoto, John J. Mulvihill, Jennifer L. Murphy, Donna M. Muzny, Michele E. Nehrebecky, Stan F. Nelson, J. Scott Newberry, John H. Newman, Sarah K. Nicholas, Donna Novacic, Jordan S. Orange, J. Carl Pallais, Christina G.S. Palmer, Jeanette C. Papp, Neil H. Parker, Loren D.M. Pena, John A. Phillips III, Jennifer E. Posey, John H. Postlethwait, Lorraine Potocki, Barbara N. Pusey, Chloe M. Reuter, Amy K. Robertson, Lance H. Rodan, Jill A. Rosenfeld, Jacinda B. Sampson, Susan L. Samson, Kelly Schoch, Molly C. Schroeder, Daryl A. Scott, Prashant Sharma, Vandana Shashi, Edwin K. Silverman, Janet S. Sinsheimer, Kevin S. Smith, Rebecca C. Spillmann, Kimberly Splinter, Joan M. Stoler, Nicholas Stong, Jennifer A. Sullivan, David A. Sweetser, Cynthia J. Tifft, Camilo Toro, Alyssa A. Tran, Tiina K. Urv, Zaheer M. Valivullah, Eric Vilain, Tiphanie P. Vogel, Colleen E. Wahl, Nicole M. Walley, Chris A. Walsh, Patricia A. Ward, Katrina M. Waters, Monte Westerfield, Anastasia L. Wise, Lynne A. Wolfe, Elizabeth A. Worthey, Shinya Yamamoto, Yaping Yang, Guoyun Yu, Diane B. Zastrow, and Allison Zheng.

Conflicts of Interest

M.S. is a cofounder and member of the scientific advisory board of Personalis, SensOmics, and Qbio. M.S. is a member of the scientific advisory board of Genapsys and Epinomics. J.D.M. is a member of the clinical advisory board for Rainbow Genomics and the scientific advisory board for Genoox. E.A.A. is a founder and member of the scientific advisory board of Personalis and Deepcell. E.A.A. is an advisor to Genome Medical and Sequencebio. M.T.W. has a minor ownership interest in Personalis.

Acknowledgments

We thank L. Duraine, Z. Zuo, K. Schulze, A. Chang, and Y. He for technical support. Research reported in this manuscript was supported by the NIH Common Fund through the Office of Strategic Coordination and Office of the NIH Director (award numbers U01HG007708, U01HG007942, U01TR001395, and U54NS093793), the NIH National Center for Advancing Translational Sciences (UL1TR001085), the NIH National Center for Research Resources (S10 RR026780), the NIH National Human Genome Research Institute (P50HG007735), the Wellcome Centre for Mitochondrial Research (203105/Z/16/Z), the Medical Research Council Centre for Translational Research in Neuromuscular Disease, the UK Mitochondrial Disease Patient Cohort (G0800674), the Lily Foundation, the UK National Health Service Highly Specialised Service for Rare Mitochondrial Disorders of Adults and Children, E-Rare project GENOMIT by the Austrian Science Fonds (I 2741-B26), and the Department of Health via the National Institute for Health Research comprehensive Biomedical Research Centre award to Guy's and St. Thomas' NHS Foundation Trust in partnership with King's College London. Portions of the metabolomics and lipidomics analyses were performed in the Environmental Molecular Sciences Laboratory, a national scientific user facility sponsored by the Department of Energy (DOE) Office of Biological and Environmental Research and located at Pacific Northwest National Laboratory (PNNL). PNNL is operated by Battelle Memorial Institute for the DOE under contract DE-AC05-76RLO1830. M.H. was supported by a Wolfson intercalation bursary award from the UK Royal College of Physicians. The content is solely the responsibility of the authors and does not necessarily represent the official views of the NIH.

Received: November 27, 2017
Accepted: January 26, 2018
Published: Febraury 22, 2018

Web Resources

ClinVar, https://www.ncbi.nlm.nih.gov/clinvar/
dbGaP, https://www.ncbi.nlm.nih.gov/gap
GenBank, https://www.ncbi.nlm.nih.gov/genbank/
genome Aggregation Database (gnomAD) Browser, http://gnomad.broadinstitute.org
OMIM, http://www.omim.org

References

1. Scaglia, F. (2012). Nuclear gene defects in mitochondrial disorders. Methods Mol. Biol. *837*, 17–34.
2. Taylor, R.W., Pyle, A., Griffin, H., Blakely, E.L., Duff, J., He, L., Smertenko, T., Alston, C.L., Neeve, V.C., Best, A., et al. (2014). Use of whole-exome sequencing to determine the genetic basis of multiple mitochondrial respiratory chain complex deficiencies. JAMA *312*, 68–77.

3. Capaldi, R.A. (1994). F1-ATPase in a spin. Nat. Struct. Biol. *1*, 660–663.
4. Schapira, A.H.V. (2006). Mitochondrial disease. Lancet *368*, 70–82.
5. Jonckheere, A.I., Smeitink, J.A.M., and Rodenburg, R.J.T. (2012). Mitochondrial ATP synthase: architecture, function and pathology. J. Inherit. Metab. Dis. *35*, 211–225.
6. Houstek, J., Pícková, A., Vojtísková, A., Mrácek, T., Pecina, P., and Jesina, P. (2006). Mitochondrial diseases and genetic defects of ATP synthase. Biochim. Biophys. Acta *1757*, 1400–1405.
7. Rodenburg, R.J.T. (2011). Biochemical diagnosis of mitochondrial disorders. J. Inherit. Metab. Dis. *34*, 283–292.
8. Wechsler, D. (2002). Wechsler Preschool and Primary Scale of Intelligence, Third Edition (The Psychological Corporation).
9. Cohen, M.J. (1997). Children's Memory Scale (The Psychological Corporation).
10. Simpson, M.A., Irving, M.D., Asilmaz, E., Gray, M.J., Dafou, D., Elmslie, F.V., Mansour, S., Holder, S.E., Brain, C.E., Burton, B.K., et al. (2011). Mutations in NOTCH2 cause Hajdu-Cheney syndrome, a disorder of severe and progressive bone loss. Nat. Genet. *43*, 303–305.
11. Thompson, K., Majd, H., Dallabona, C., Reinson, K., King, M.S., Alston, C.L., He, L., Lodi, T., Jones, S.A., Fattal-Valevski, A., et al. (2016). Recurrent De Novo Dominant Mutations in SLC25A4 Cause Severe Early-Onset Mitochondrial Disease and Loss of Mitochondrial DNA Copy Number. Am. J. Hum. Genet. *99*, 860–876.
12. Yang, Y., Muzny, D.M., Reid, J.G., Bainbridge, M.N., Willis, A., Ward, P.A., Braxton, A., Beuten, J., Xia, F., Niu, Z., et al. (2013). Clinical whole-exome sequencing for the diagnosis of mendelian disorders. N. Engl. J. Med. *369*, 1502–1511.
13. Yang, Y., Muzny, D.M., Xia, F., Niu, Z., Person, R., Ding, Y., Ward, P., Braxton, A., Wang, M., Buhay, C., et al. (2014). Molecular findings among patients referred for clinical whole-exome sequencing. JAMA *312*, 1870–1879.
14. Jordan, E.M., and Breen, G.A. (1992). Molecular cloning of an import precursor of the delta-subunit of the human mitochondrial ATP synthase complex. Biochim. Biophys. Acta *1130*, 123–126.
15. Biasini, M., Bienert, S., Waterhouse, A., Arnold, K., Studer, G., Schmidt, T., Kiefer, F., Gallo Cassarino, T., Bertoni, M., Bordoli, L., and Schwede, T. (2014). SWISS-MODEL: modelling protein tertiary and quaternary structure using evolutionary information. Nucleic Acids Res. *42*, W252–W258.
16. Dewey, F.E., Murray, M.F., Overton, J.D., Habegger, L., Leader, J.B., Fetterolf, S.N., O'Dushlaine, C., Van Hout, C.V., Staples, J., Gonzaga-Jauregui, C., et al. (2016). Distribution and clinical impact of functional variants in 50,726 whole-exome sequences from the DiscovEHR study. Science *354*, aaf6814.
17. Oláhová, M., Haack, T.B., Alston, C.L., Houghton, J.A., He, L., Morris, A.A., Brown, G.K., McFarland, R., Chrzanowska-Lightowlers, Z.M., Lightowlers, R.N., et al. (2015). A truncating PET100 variant causing fatal infantile lactic acidosis and isolated cytochrome c oxidase deficiency. Eur. J. Hum. Genet. *23*, 935–939.
18. Oláhová, M., Hardy, S.A., Hall, J., Yarham, J.W., Haack, T.B., Wilson, W.C., Alston, C.L., He, L., Aznauryan, E., Brown, R.M., et al. (2015). LRPPRC mutations cause early-onset multisystem mitochondrial disease outside of the French-Canadian population. Brain *138*, 3503–3519.
19. Wang, J., Al-Ouran, R., Hu, Y., Kim, S.-Y., Wan, Y.-W., Wangler, M.F., Yamamoto, S., Chao, H.-T., Comjean, A., Mohr, S.E., et al.; UDN (2017). MARRVEL: Integration of Human and Model Organism Genetic Resources to Facilitate Functional Annotation of the Human Genome. Am. J. Hum. Genet. *100*, 843–853.
20. Brand, A.H., and Perrimon, N. (1993). Targeted gene expression as a means of altering cell fates and generating dominant phenotypes. Development *118*, 401–415.
21. Lee, T., and Luo, L. (1999). Mosaic analysis with a repressible cell marker for studies of gene function in neuronal morphogenesis. Neuron *22*, 451–461.
22. Teixeira, F.K., Sanchez, C.G., Hurd, T.R., Seifert, J.R.K., Czech, B., Preall, J.B., Hannon, G.J., and Lehmann, R. (2015). ATP synthase promotes germ cell differentiation independent of oxidative phosphorylation. Nat. Cell Biol. *17*, 689–696.
23. Nagarkar-Jaiswal, S., Manivannan, S.N., Zuo, Z., and Bellen, H.J. (2017). A cell cycle-independent, conditional gene inactivation strategy for differentially tagging wild-type and mutant cells. eLife *6*, e26420.
24. Contrepois, K., Jiang, L., and Snyder, M. (2015). Optimized Analytical Procedures for the Untargeted Metabolomic Profiling of Human Urine and Plasma by Combining Hydrophilic Interaction (HILIC) and Reverse-Phase Liquid Chromatography (RPLC)-Mass Spectrometry. Mol. Cell. Proteomics *14*, 1684–1695.
25. Chennamsetty, I., Coronado, M., Contrepois, K., Keller, M.P., Carcamo-Orive, I., Sandin, J., Fajardo, G., Whittle, A.J., Fathzadeh, M., Snyder, M., et al. (2016). Nat1 Deficiency Is Associated with Mitochondrial Dysfunction and Exercise Intolerance in Mice. Cell Rep. *17*, 527–540.
26. Spincemaille, P., Matmati, N., Hannun, Y.A., Cammue, B.P.A., and Thevissen, K. (2014). Sphingolipids and mitochondrial function in budding yeast. Biochim. Biophys. Acta *1840*, 3131–3137.
27. Hernández-Corbacho, M.J., Salama, M.F., Canals, D., Senkal, C.E., and Obeid, L.M. (2017). Sphingolipids in mitochondria. Biochim. Biophys. Acta *1862*, 56–68.
28. Knupp, J., Martinez-Montañés, F., Van Den Bergh, F., Cottier, S., Schneiter, R., Beard, D., and Chang, A. (2017). Sphingolipid accumulation causes mitochondrial dysregulation and cell death. Cell Death Differ. *24*, 2044–2053.
29. Kogot-Levin, A., and Saada, A. (2014). Ceramide and the mitochondrial respiratory chain. Biochimie *100*, 88–94.
30. Acehan, D., Malhotra, A., Xu, Y., Ren, M., Stokes, D.L., and Schlame, M. (2011). Cardiolipin affects the supramolecular organization of ATP synthase in mitochondria. Biophys. J. *100*, 2184–2192.
31. Strauss, M., Hofhaus, G., Schröder, R.R., and Kühlbrandt, W. (2008). Dimer ribbons of ATP synthase shape the inner mitochondrial membrane. EMBO J. *27*, 1154–1160.
32. Paumard, P., Arselin, G., Vaillier, J., Chaignepain, S., Bathany, K., Schmitter, J.M., Brèthes, D., and Velours, J. (2002). Two ATP synthases can be linked through subunits i in the inner mitochondrial membrane of Saccharomyces cerevisiae. Biochemistry *41*, 10390–10396.
33. Jackson, C.B., Hahn, D., Schröter, B., Richter, U., Battersby, B.J., Schmitt-Mechelke, T., Marttinen, P., Nuoffer, J.-M., and Schaller, A. (2017). A novel mitochondrial ATP6 frameshift mutation causing isolated complex V deficiency, ataxia and encephalomyopathy. Eur. J. Med. Genet. *60*, 345–351.

34. Paumard, P., Vaillier, J., Coulary, B., Schaeffer, J., Soubannier, V., Mueller, D.M., Brèthes, D., di Rago, J.-P., and Velours, J. (2002). The ATP synthase is involved in generating mitochondrial cristae morphology. EMBO J. *21*, 221–230.
35. Owusu-Ansah, E., Yavari, A., Mandal, S., and Banerjee, U. (2008). Distinct mitochondrial retrograde signals control the G1-S cell cycle checkpoint. Nat. Genet. *40*, 356–361.
36. McGary, K.L., Park, T.J., Woods, J.O., Cha, H.J., Wallingford, J.B., and Marcotte, E.M. (2010). Systematic discovery of nonobvious human disease models through orthologous phenotypes. Proc. Natl. Acad. Sci. USA *107*, 6544–6549.
37. Holt, I.J., Harding, A.E., Petty, R.K., and Morgan-Hughes, J.A. (1990). A new mitochondrial disease associated with mitochondrial DNA heteroplasmy. Am. J. Hum. Genet. *46*, 428–433.
38. Jonckheere, A.I., Hogeveen, M., Nijtmans, L.G.J., van den Brand, M.A.M., Janssen, A.J.M., Diepstra, J.H.S., van den Brandt, F.C.A., van den Heuvel, L.P., Hol, F.A., Hofste, T.G.J., et al. (2008). A novel mitochondrial ATP8 gene mutation in a patient with apical hypertrophic cardiomyopathy and neuropathy. J. Med. Genet. *45*, 129–133.
39. Ware, S.M., El-Hassan, N., Kahler, S.G., Zhang, Q., Ma, Y.-W., Miller, E., Wong, B., Spicer, R.L., Craigen, W.J., Kozel, B.A., et al. (2009). Infantile cardiomyopathy caused by a mutation in the overlapping region of mitochondrial ATPase 6 and 8 genes. J. Med. Genet. *46*, 308–314.
40. Mkaouar-Rebai, E., Kammoun, F., Chamkha, I., Kammoun, N., Hsairi, I., Triki, C., and Fakhfakh, F. (2010). A de novo mutation in the adenosine triphosphatase (ATPase) 8 gene in a patient with mitochondrial disorder. J. Child Neurol. *25*, 770–775.
41. De Meirleir, L., Seneca, S., Lissens, W., De Clercq, I., Eyskens, F., Gerlo, E., Smet, J., and Van Coster, R. (2004). Respiratory chain complex V deficiency due to a mutation in the assembly gene ATP12. J. Med. Genet. *41*, 120–124.
42. Meulemans, A., Seneca, S., Pribyl, T., Smet, J., Alderweirldt, V., Waeytens, A., Lissens, W., Van Coster, R., De Meirleir, L., di Rago, J.-P., et al. (2010). Defining the pathogenesis of the human Atp12p W94R mutation using a Saccharomyces cerevisiae yeast model. J. Biol. Chem. *285*, 4099–4109.
43. Mayr, J.A., Havlícková, V., Zimmermann, F., Magler, I., Kaplanová, V., Jesina, P., Pecinová, A., Nusková, H., Koch, J., Sperl, W., and Houstek, J. (2010). Mitochondrial ATP synthase deficiency due to a mutation in the ATP5E gene for the F1 epsilon subunit. Hum. Mol. Genet. *19*, 3430–3439.
44. Jonckheere, A.I., Renkema, G.H., Bras, M., van den Heuvel, L.P., Hoischen, A., Gilissen, C., Nabuurs, S.B., Huynen, M.A., de Vries, M.C., Smeitink, J.A.M., and Rodenburg, R.J. (2013). A complex V ATP5A1 defect causes fatal neonatal mitochondrial encephalopathy. Brain *136*, 1544–1554.
45. Lieber, D.S., Calvo, S.E., Shanahan, K., Slate, N.G., Liu, S., Hershman, S.G., Gold, N.B., Chapman, B.A., Thorburn, D.R., Berry, G.T., et al. (2013). Targeted exome sequencing of suspected mitochondrial disorders. Neurology *80*, 1762–1770.
46. Magner, M., Dvorakova, V., Tesarova, M., Mazurova, S., Hansikova, H., Zahorec, M., Brennerova, K., Bzduch, V., Spiegel, R., Horovitz, Y., et al. (2015). TMEM70 deficiency: long-term outcome of 48 patients. J. Inherit. Metab. Dis. *38*, 417–426.
47. Hejzlarová, K., Tesařová, M., Vrbacká-Čížková, A., Vrbacký, M., Hartmannová, H., Kaplanová, V., Nosková, L., Kratochvílová, H., Buzková, J., Havlíčková, V., et al. (2011). Expression and processing of the TMEM70 protein. Biochim. Biophys. Acta *1807*, 144–149.
48. Cízková, A., Stránecký, V., Mayr, J.A., Tesarová, M., Havlícková, V., Paul, J., Ivánek, R., Kuss, A.W., Hansíková, H., Kaplanová, V., et al. (2008). TMEM70 mutations cause isolated ATP synthase deficiency and neonatal mitochondrial encephalocardiomyopathy. Nat. Genet. *40*, 1288–1290.
49. Honzík, T., Tesarová, M., Mayr, J.A., Hansíková, H., Jesina, P., Bodamer, O., Koch, J., Magner, M., Freisinger, P., Huemer, M., et al. (2010). Mitochondrial encephalocardio-myopathy with early neonatal onset due to TMEM70 mutation. Arch. Dis. Child. *95*, 296–301.
50. Houstek, J., Mrácek, T., Vojtísková, A., and Zeman, J. (2004). Mitochondrial diseases and ATPase defects of nuclear origin. Biochim. Biophys. Acta *1658*, 115–121.
51. Hejzlarová, K., Mráček, T., Vrbacký, M., Kaplanová, V., Karbanová, V., Nůsková, H., Pecina, P., and Houštěk, J. (2014). Nuclear genetic defects of mitochondrial ATP synthase. Physiol. Res. *63* (*Suppl 1*), S57–S71.
52. Sarajlija, A., Magner, M., Djordjevic, M., Kecman, B., Grujic, B., Tesarova, M., and Minic, P. (2017). Late-presenting congenital diaphragmatic hernia in a child with TMEM70 deficiency. Congenit. Anom. (Kyoto) *57*, 64–65.
53. Wortmann, S.B., Kluijtmans, L.A.J., Rodenburg, R.J., Sass, J.O., Nouws, J., van Kaauwen, E.P., Kleefstra, T., Tranebjaerg, L., de Vries, M.C., Isohanni, P., et al. (2013). 3-Methylglutaconic aciduria–lessons from 50 genes and 977 patients. J. Inherit. Metab. Dis. *36*, 913–921.

The American Journal of Human Genetics, Volume *102*

Supplemental Data

Biallelic Mutations in *ATP5F1D*, which Encodes a Subunit of ATP Synthase, Cause a Metabolic Disorder

Monika Oláhová, Wan Hee Yoon, Kyle Thompson, Sharayu Jangam, Liliana Fernandez, Jean M. Davidson, Jennifer E. Kyle, Megan E. Grove, Dianna G. Fisk, Jennefer N. Kohler, Matthew Holmes, Annika M. Dries, Yong Huang, Chunli Zhao, Kévin Contrepois, Zachary Zappala, Laure Frésard, Daryl Waggott, Erika M. Zink, Young-Mo Kim, Heino M. Heyman, Kelly G. Stratton, Bobbie-Jo M. Webb-Robertson, Undiagnosed Diseases Network, Michael Snyder, Jason D. Merker, Stephen B. Montgomery, Paul G. Fisher, René G. Feichtinger, Johannes A. Mayr, Julie Hall, Ines A. Barbosa, Michael A. Simpson, Charu Deshpande, Katrina M. Waters, David M. Koeller, Thomas O. Metz, Andrew A. Morris, Susan Schelley, Tina Cowan, Marisa W. Friederich, Robert McFarland, Johan L.K. Van Hove, Gregory M. Enns, Shinya Yamamoto, Euan A. Ashley, Michael F. Wangler, Robert W. Taylor, Hugo J. Bellen, Jonathan A. Bernstein, and Matthew T. Wheeler

FIGURE S1

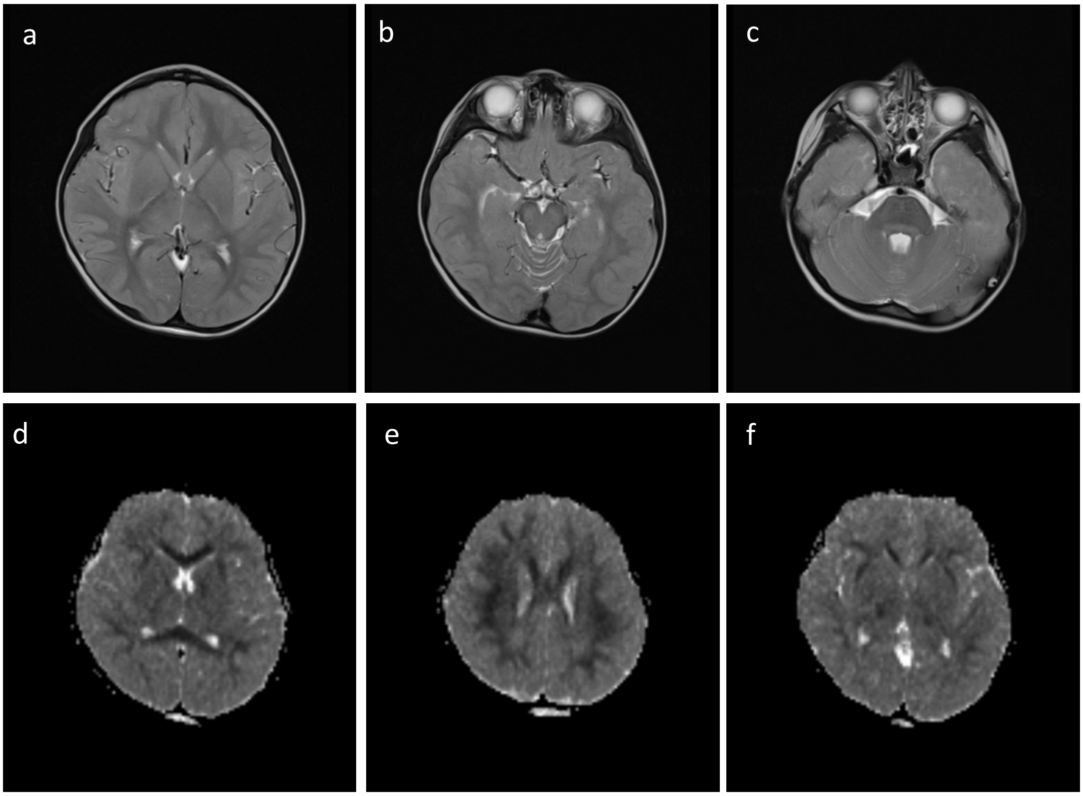

Figure S1. MRI Findings in subject 2
Subject 2 MRI at age 4 years 10 months demonstrated generalised brain swelling (a) with more distinctive subcortical white matter T2 hyperintensity within the temporal lobes bilaterally. There was also distinctive abnormal T2 hyperintensity within the midbrain (b), posterior pons (c) and dentate nuclei. There was a symmetrical pattern of restricted diffusion involving the corpus callosum (d), subcortical white matter of both cerebral hemispheres (e), corticospinal tracts (f), midbrain, pons and cerebellum. All of these changes resolved on follow-up imaging one year later. a = Axial T2SE; b = Axial T2SE; c = Axial T2SE; d = Axial ADC Map; e= Axial ADC Map; f = Axial ADC Map.

FIGURE S2

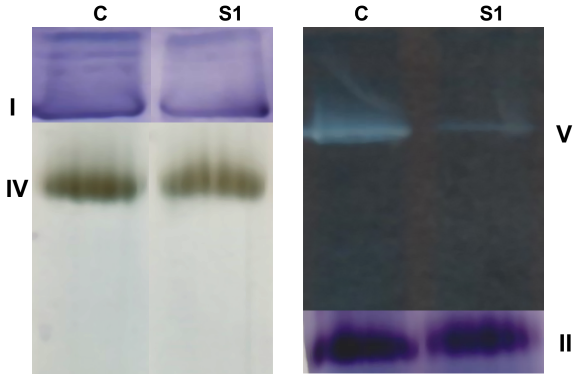

Figure S2. Subject 1 fibroblast studies show reduced in gel activity of complex V. Blue native PAGE with in-gel activity stain performed as described showed reduced activity of complex V in subject fibroblasts[1]. Mitochondrial membrane fractions were isolated from fibroblasts of subject 1 and analyzed by blue native polyacrylamide gel electrophoresis (BN-PAGE) with in-gel activity staining for complexes I, II, IV, and V as indicated for both control (C) and subject 1 (S1). The activity for complex V was reduced in the subject while the activities of complexes I, II, and IV were normal. There were no additional bands of lower molecular weight in complex V, as would be typically seen in disorders affecting the synthesis of the mtDNA-encoded subunits of complex V[1], but similar to that noted with defects in *ATP5F1E*, *ATP5F1A*, and *ATPAF2*, each affecting the assembly of the F_1 subunit.

FIGURE S3

A

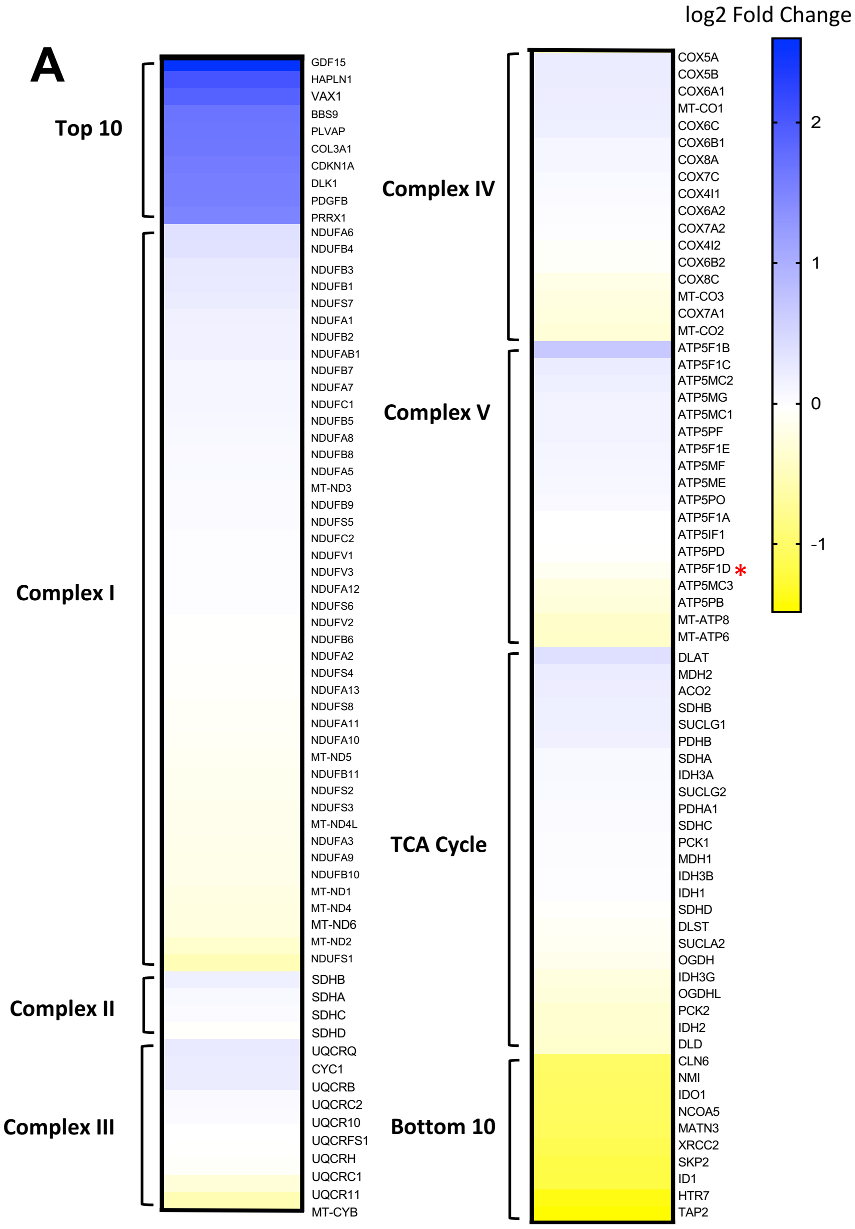

Figure S3. Expression profile of Subject Derived Cells RNA was extracted from cultured iPSC cells utilizing TRIzol. cDNA libraries were constructed using the Illumina TruSeq Stranded mRNA Sample Prep kit. Pooled libraries were run on each a NextSeq 500, using high output flowcells. Sequences were read as paired end 150 cycles (2x150). All base call files (bcl) were converted by in-house script to FASTQ format, compiled and demultiplexed and total read counts were determined for each sample. Initial read quality was determined by utilizing the program FASTQC. All reads were processed by adapter trimming, Kmer removal and the remaining reads mapped to HG19. **(A)** Heat map of RNAseq data from subject 1 ($\mathit{ATP5F1D}^{P82L}$) in comparison to unaffected control samples shows no evidence of dysregulation in *ATP5F1D* expression (red asterisk). Expression levels of other complex I-V components are not significantly altered. Expression is measured via log2 fold change compared to unaffected controls. Top and bottom ten differentially expressed protein coding genes also included. Differential splicing was not seen for *ATP5F1D* transcripts (data not shown). **(B)** Top ten biological processes identified by GO term analysis of top 200 differentially expressed protein coding genes from RNAseq data set. No significant pathways observed on bottom 200 differentially expressed protein coding genes.

B

GO biological process	Actual # Genes in Top 200	Expected in Top 200	Fold Enrichment	P value
positive regulation of endothelial cell migration	7	0.63	11.04	0.04
response to mechanical stimulus	11	1.84	5.97	0.03
extracellular matrix organization	14	2.65	5.29	0.005
regulation of ERK1 and ERK2 cascade	12	2.28	5.26	0.03
angiogenesis	13	2.53	5.14	0.02
response to growth factor	18	4.29	4.19	0.003
positive regulation of apoptotic process	21	5.15	4.08	0.0005
negative regulation of phosphate metabolic process	18	4.95	3.64	0.03
negative regulation of signal transduction	34	9.98	3.41	0.000003
negative regulation of transcription from RNA polymerase II promoter	23	6.98	3.3	0.005

FIGURE S4

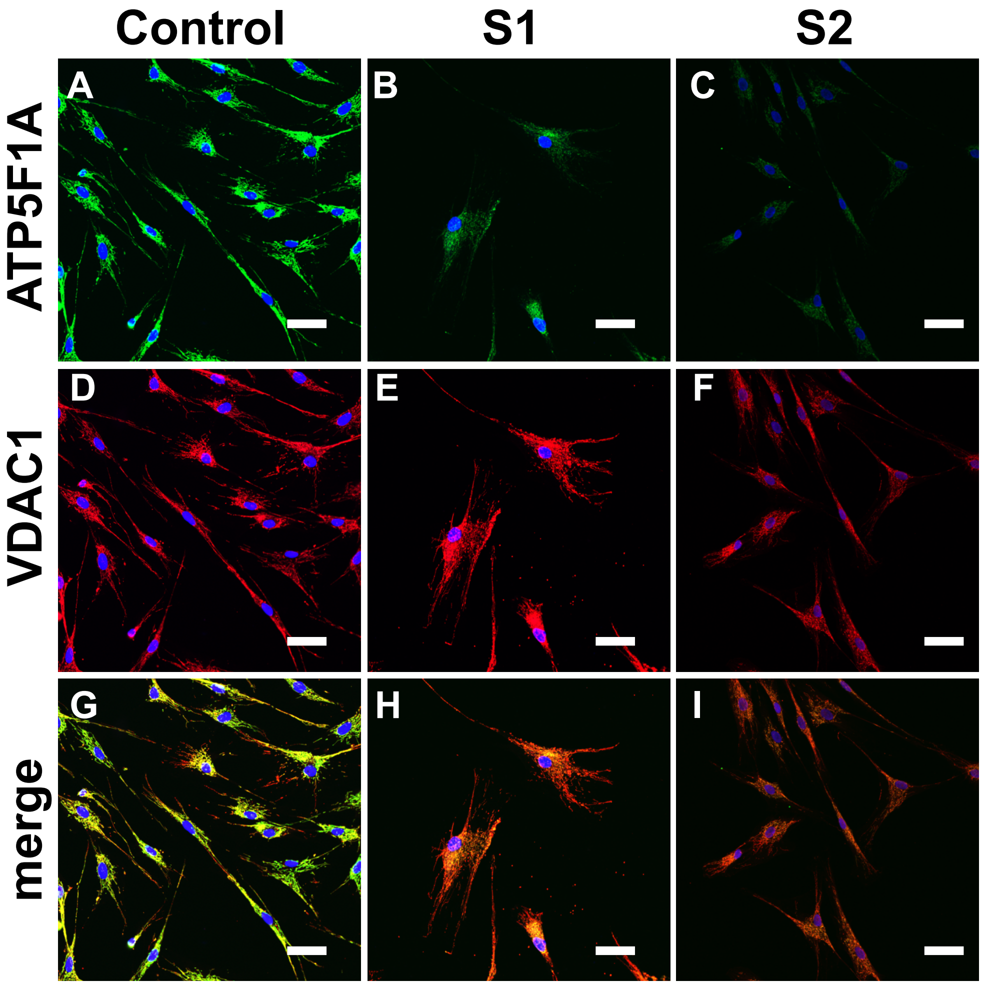

Figure S4: Cultured skin fibroblasts from affected individuals show a complex V defect. Immunofluorescence staining of fibroblasts obtained from affected individuals and control was performed using anti-ATP5F1A antibody (1:1000; ab14748, Abcam, Cambridge, UK) (**A-C**) and anti-VDAC1 (1:400; ab15895, Abcam, Cambridge, UK) (**D-F**), with the overlay (**G-I**) demonstrating strong staining of the complex V protein in the controls and absence in the subject 1 and 2 (S1 and S2) cell lines with preserved mitochondrial voltage dependent anion channel staining in subject cells (Scale bar = 50 µm). Fibroblasts were grown on chamber slides. Cells were allowed to attach for 24 hours. At the next day, the medium was removed, and chamber slides were twice washed with PBS pH 7.4 and fixed in formalin overnight at 4°C. After washing cells three times 3 min with PBS-T (pH 7.5; 0.05% Tween-20), heat-induced epitope retrieval was done in 1 mM EDTA, 0.01% Tween-20, pH 8 at 95°C for 45 min. The solution was allowed to cool down to room temperature and chamber slides were washed with PBS-T. The chamber slides were incubated 1 h at RT with primary antibodies against rabbit-anti-ATP5F1A antibody (1:1000; ab14748, Abcam, Cambridge, UK) and anti-VDAC1 (1:400; ab15895, Abcam, Cambridge, UK). Primary antibodies were diluted in DAKO antibody diluent with background-reducing components. After washing with PBS-T, cells were incubated 1 h at RT in dark with secondary antibodies (Alexa Fluor 594 donkey anti-rabbit antibody, VXA21207, Life Technologies, Carlsbad, US, 1 : 500 and Alexa Fluor 488 donkey anti-mouse IgG (H + L), VXA21202, Carlsbad, US, 1 : 1000). After washing the chamber slides with PBS-T, they were incubated with DAPI diluted 1 : 2000 in PBS-T for 10 min. Chamber slides were mounted in fluorescence mounting media from DAKO.

FIGURE S5

A

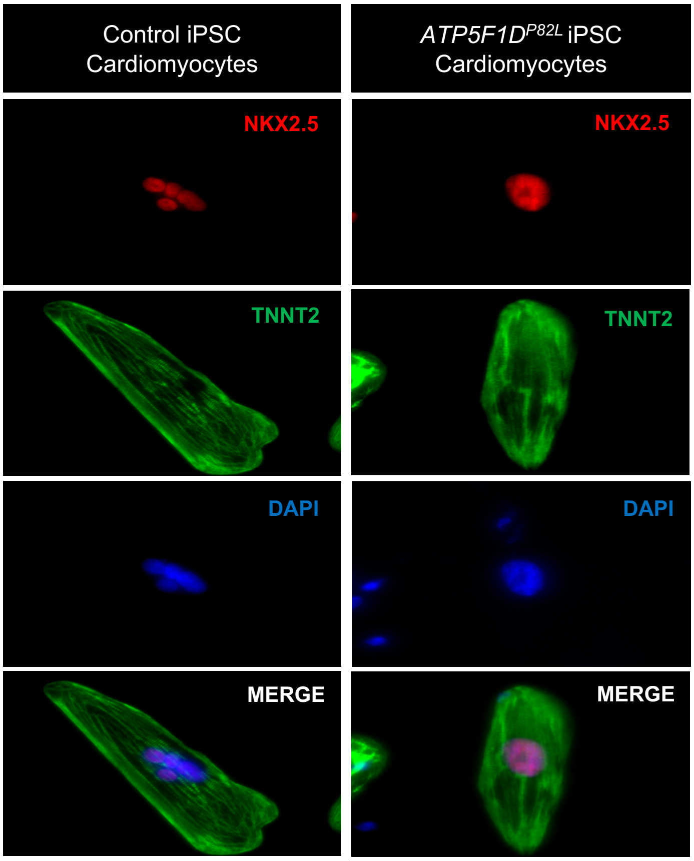

Figure S5. (A) *ATP5F1D^{P82L}* iPSC derived cardiomyocytes iPSCs were reprogrammed with Sendai Virus from subject 1 biopsy skin fibroblast. The iPSCs were then cultured in serum-free/feeder free medium hStemSFM (Stemmera, ST02001) on a matrigel coated plates for 20 passages. 70-80% confluent cells were differentiated to cardiomyocytes we added 2 ml of RPMI medium with B27 supplement minus insulin with 4-6 uM of CHIR-99021 for 2 days. Cells were then treated with RPMI plus B27 minus insulin plus 5uM IWR1 for 2 days, RPMI plus B27 minus insulin for 2 days, then RPMI plus B27 plus insulin for 4 days. To purify the cardiomyocytes, lactate medium has been appliedon day 14. Staining for rabbit anti-NKX2.5 and mouse anti-TTNT2 on day 30 post-differentiation.Control and subject 1 derived (*ATP5F1D^{P82L}*) iPS cells were differentiated into cardiomyocytes, both displaying characteristic staining of TNNT2 and NKX2.5 confirming commitment to cardiomyocyte lineage.

B

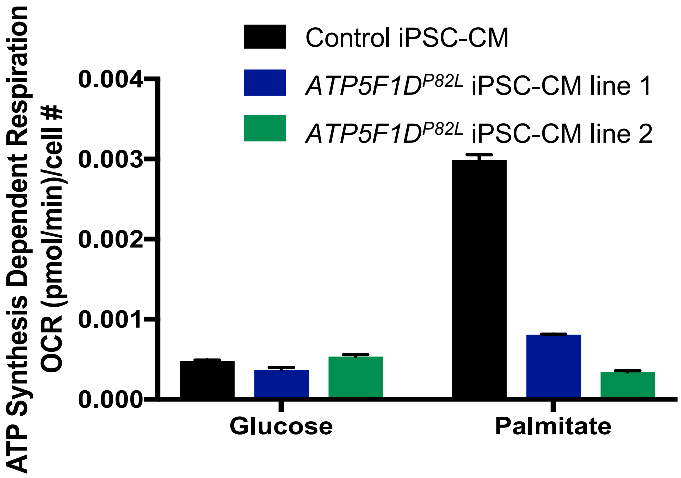

(B) In vitro oxygen consumption assay Seahorse (Agilent Technologies) plate wells were coated with Matrigel overnight. 30,000 Cardiomyocytes per well, differentiated from control and two *ATP5F1D^{P82L}* iPSC lines were plated the following day. Cells were maintained in lactate medium for 3 days. Diluted oligomycin, FCCP and Rotenone/Antimycin a (AA/Rot) were prepared per manufacturer instructions. Cell medium was changed to add glucose or palmitate. Oxygen consumption rate (pmol/min) was determined for each substrate/cell line/drug combination performed in triplicate. Oxygen consumption rate was normalized to viable cell count determined by vital dye staining performed on each well at the completion of the experiment. *ATP5F1D^{P82L}* cardiomyocytes have impaired ATP synthase dependent respiration oxygen consumption rate (OCR) in response to palmitate, when compared to normal cardiomyocytes. Two different iPSC cardiomyocyte lines derived from subject 1 showed decreased ATP synthesis dependent respiration with palmitate as compared to a wildtype control. There was no significant difference in respiration between control and *ATP5F1D^{P82L}* cardiomyocytes when substrate was glucose at nonlimiting concentrations.

FIGURE S6

ey-Gal4> UAS-lacZ

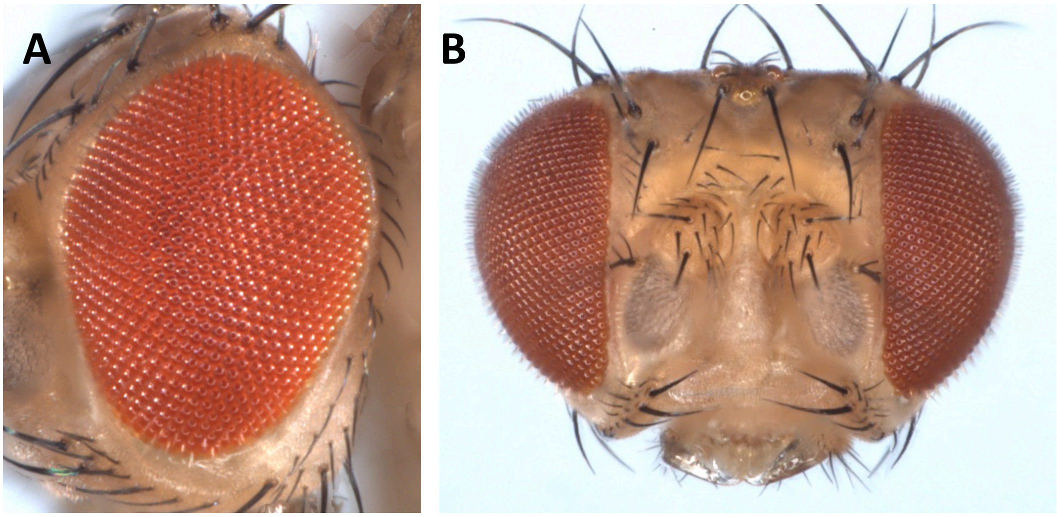

C

Gal4-drivers / Transgene	*Tubulin-Gal4* (ubiquitous)	*Actin-Gal4* (ubiquitous)	*da-Gal4* (ubiquitous)
UAS–empty construct	Viable	Viable	Viable
UAS-ATPsynδ RNAi	Lethal	Lethal	Lethal

D

Transgene	$elav^{[C155]}$*-Gal4* (neuronal)
UAS-ATPsynδ RNAi	Lethal
UAS-ATPsynδ RNAi; UAS-$ATP5F1D^{WT}$	Viable
UAS-ATPsynδ RNAi; UAS-$ATP5F1D^{P82L}$	Lethal
UAS-ATPsynδ RNAi; UAS-$ATP5F1D^{V106G}$	Lethal

Figure S6: Drosophila overexpression studies.
(**A**-**B**) Light micrographs of an eye (**A**) and antennae (**B**) of flies (*ey-Gal4/+ ; UAS-lacZ/+*) are shown to represent the control eye and antennae morphology. For taking *Drosophila* eye and the antennal images, flies were frozen in -20 °C overnight. Images were obtained using a digital camera (MicroFire; Olympus) mounted on a stereomicroscope (MZ16; Leica) and ImagePro Plus 7.0 acquisition software (Media Cybernetics). The Extended Focus Function of the ImagePro software was used to obtain stacked images. The images were further processed in ImageJ software. **(C)** shows that expression of the *ATPsynδ* RNAi by various ubiquitous *Gal4* drivers including *tub-Gal4*, *Actin-Gal4*, or *da-Gal4* causes lethality, while control transgene (*UAS-empty*) expression does not. **(D)** shows that expression by the neuronal specific driver ($elav^{[C155]}$*-Gal4*) of the *ATPsynδ* RNAi is lethal; this lethality is rescued by human normal *ATP5F1D* and is not rescued by *ATP5F1D* p.P82L or p.V106G variants. See Figure S7 legend for details of ATP5F1D transgenics.

The following stocks were obtained from the Bloomington Stock Center at Indiana University (BDSC).

- y1 w*; tubulin-Gal4/ TM3, Sb1, Ser1
- w*; Actin-Gal4/CyO
- w; da-Gal4 (on III)
- ey-gal4 (on II)
- *w*; Sco/CyO; P{w[+mC]=tubP-GAL80*^{ts}*}7*
- *w*; P{w[+mC]=tubP-GAL80*^{ts}*}20; TM2/TM6B, Tb*^{1}

ATPsynδ RNAi lines (v100621) were obtained from the Vienna Drosophila Resource Center.[2] All flies were maintained at room temperature (21°C). All crosses were kept at 25°C except those for the lethality experiment (28°C).

FIGURE S7

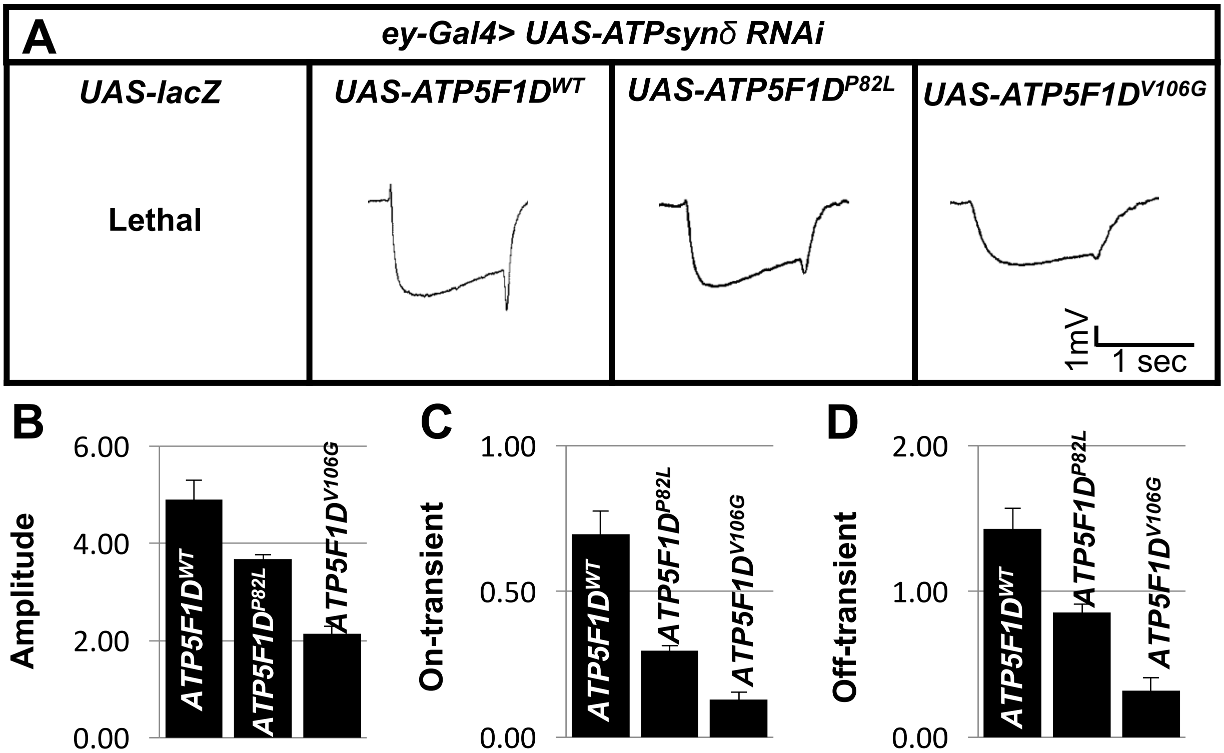

Figure S7: Electroretinogram studies on human ATP5F1D transgene-rescued flies. Plasmids carrying ATP5F1D cDNA with P82L variant and ATP5F1D cDNA with V106G were generated by site-directed mutagenesis PCR from a human ATP5F1D cDNA clone (HsCD00506484, DNASU Plasmid Repository) using primers: ATP5D_p.P82L-F: 5'- cccacgctgcaggtcctgcggcTggggctg-gtcgtggtgcatgca-3', ATP5D_p.P82L-R: 5'- tgcatgcaccacgaccagccccAgccgcaggacctgcagcgtggg-3', ATP5D_p.V106G-F: 5'- gtga-gcagcggttccatcgcagGgaacgccgactcttcggtgcag-3', and ATP5D_p.V106G-R: 5'-ctgcaccgaagagtcggcgttcCctgcgatggaaccgctgctcac-3'. For construction of pUASTattB-human ATP5D-V5, pUASTattB-human ATP5D (p.P82L)-V5, and pUASTattB-human ATP5D (p.V106G)-V5, full-length ATP5F1D cDNAs were amplified by PCR from wild type ATP5F1D cDNA, ATP5F1D (P82L), and ATP5F1D (V106G) clones, and then subcloned into BglII/NotI sites in the pUASTattB vector using primers: ATP5D_F BglII: 5'-AGATCTcaaaATGCTGCCCGCCGCGCTG-3', ATP5D -V5_R NotI: 5'-GCGGCCGCTTAGGTGCTATCCAGTCCGAGCAGTGGATTCGGGA-TCGGCTTGCCGCCGCTTCCCTCCAGGGCCTTCACCAGGG-3'. The pUASTattB constructs were injected into *y,w,FC31; VK33* embryos and transgenic flies were selected.[3] ERG recording was carried out as previously described[4]. Briefly, adult flies were immobilized on a glass slide with glue. A glass-recording electrode, filled with 100 mM NaCl was placed on the surface of the eye, and a glass reference electrode was inserted into the thorax. Recordings were performed after three to four minutes of darkness. A fly eye was exposed to a flash of white light for 1 sec. The responses were digitized and recorded and analyzed with AXON™-pCLAMP8 software. **(A)** Electroretinogram of flies carrying *ey-Gal4* > *UAS-ATPsynδ* RNAi, together with *UAS-ATP5F1D^{WT}*, *UAS-ATP5F1D^{P82L}* or *UAS-ATP5F1D^{V106G}*. **(B-D)** Quantification of the electroretinogram shown by amplitude **(B)**, on-transients **(C)**, and off-transients **(D)** of electroretinogram traces in **(A)**. Error bars indicate SEM.

FIGURE S8

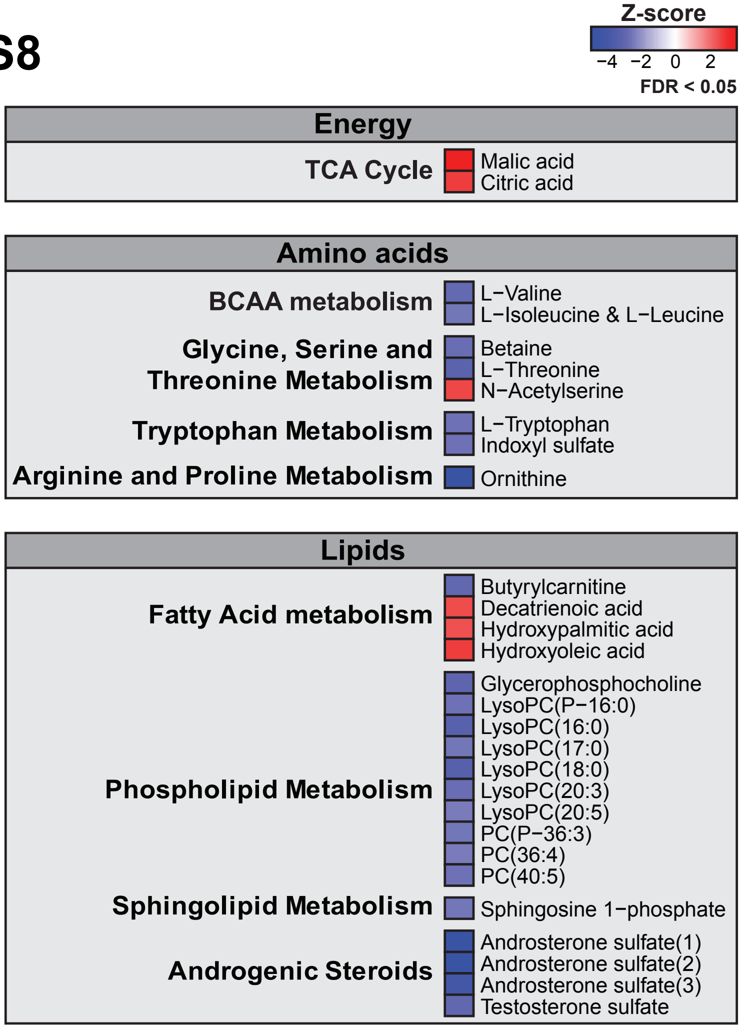

Figure S8. Untargeted plasma metabolomics by complementary HILIC- and RPLC-MS. Outlier analysis in subject 1 in comparison to 21 unrelated controls identified 41 statistically significant metabolites (FDR < 0.05) with MS signal intensity >3E7 (**Table S1**). Metabolites from plasma were extracted and analyzed as previously described.[5,6] Metabolic extracts were analyzed in HILIC ESI (+) MS, HILIC ESI (-) MS, RPLC ESI (+) MS, RPLC ESI (-) MS using a Thermo Ultimate 3000 RSLC system coupled with a Thermo Q Exactive plus mass spectrometer. The Q Exactive plus was equipped with a HESI-II probe and operated in full MS scan mode. MS/MS data were acquired on quality control samples (QCs = equimolar mixture of all the samples comprised in the study). HILIC experiments were performed using a ZIC-HILIC column 2.1 x 100 mm, 3.5 µm, 200Å (Merck Millipore) and mobile phase solvents consisting of 10 mM ammonium acetate in 50/50 acetonitrile/water (A) and 10 mM ammonium acetate in 95/5 acetonitrile/water (B).[5] Metabolites were eluted from the column at 0.5 mL/min using a 1–99% phase A gradient over 15 min. RPLC experiments were performed using a Zorbax SBaq column 2.1 x 50 mm, 1.7 µm, 100Å (Agilent Technologies) and mobile phase solvents consisting of 0.06% acetic acid in water (A) and 0.06% acetic acid in methanol (B). Metabolites were eluted from the column at 0.6 mL/min using a 1–99% phase B gradient over 9 min. Data were analyzed using an in-house data analysis pipeline written in R (version 3.0.1). Metabolite features (characterized by a unique mass/charge ratio and retention time) were extracted, aligned and quantified with the “XCMS” package (version 1.39.4) after conversion of .RAW files to .mzXML using the ProteoWizard MS convert tool. Grouping and annotation were performed with the “CAMERA” package (version 1.16.0). Features from blanks and not present in at least 66% of the samples were discarded. The signal drift with time was corrected by applying LOESS (Local Regression) normalization. After log2 transformation, Z-scores and P-values were calculated for each metabolic feature. P-values were corrected for multiple hypothesis testing using q-value correction. A FDR of 0.05 or less was considered significant. Formal identification of significant metabolites was performed by matching fragmentation spectra to public spectral libraries or by matching retention time and fragmentation spectra to authentic standards when possible.

FIGURE S9

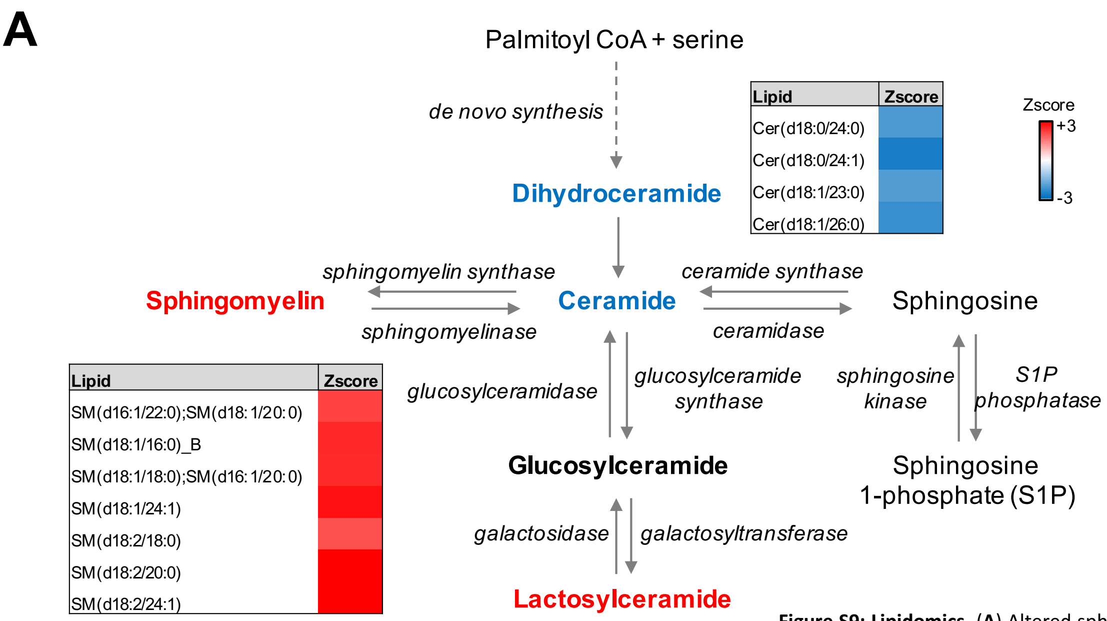

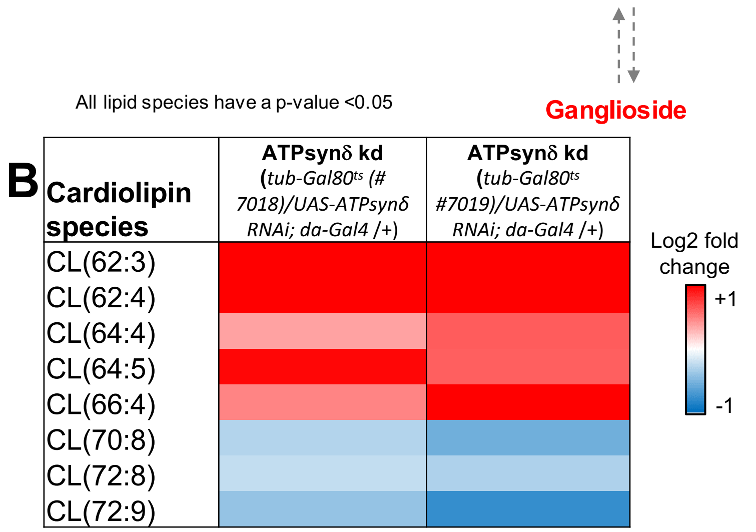

Figure S9: Lipidomics. (**A**) Altered sphingolipid metabolism in subject 1. Dihydroceramide and ceramide lipid species are statistically significantly lower (blue; p-value <0.05) while more complex lipids, in which ceramide is a precursor, are elevated (red). Heat maps represent z-score values of statistically significant lipids (p-value <0.05). Sphingosine and sphingosine-1-phosphate were not detected. (**B**) Cardiolipin species identified in *ATPsynδ* knockdown female flies (*tub-Gal80^{ts} (Bloomington # 7018 and #7019)/UAS-ATPsynδ RNAi; da-Gal4 /+*) and control flies (*tub-Gal80^{ts} (Bloomington #7019)/UAS-ATPsynδ RNAi*). As ubiquitous expression of *ATPsynδ* RNAi causes lethality, we used *tub-Gal80^{ts}* to knockdown expression of *ATPsynδ RNAi.* The cardiolipin species are organized by increasing total number of carbons and double bonds in the fatty acids. Heat maps represent log2 fold changes.

Subject 1 sample was compared to a reference database of 136 individuals that were between the ages of 0.6 to 81 years and 50% female with no known metabolic disease. In order to correct for batch effects, we included identical quality control (QC) samples in both the reference dataset and in subsequent subject datasets. Lipids were extracted by using an established chloroform/methanol extraction procedure based on a modified Folch extraction (MPLEx).[7] For both the reference and subject plasma samples, 50 µl of plasma was transferred to 2.0 mL Sorenson low-binding microcentrifuge tubes to which 250 µl of cold (-20°C) chloroform/methanol (2:1, v/v) was added. Samples are vortexed for 10 s and incubated at 4°C for 5 minutes, and then vortexed again for 10 s. Then, samples are centrifuged to facilitate separation of a hydrophilic layer containing polar metabolites and a hydrophobic layer containing lipids. The hydrophobic lipid layer was removed and placed into new microcentrifuge tubes and evaporated to dryness in vacuo. Lipid extracts are stored at -20°C in chloroform/methanol (2:1, v/v) until LC-MS analysis. Prior to MS analysis, total lipid extracts (TLEs) were dried and then reconstituted in 200 µl of methanol. LC-MS/MS parameters and lipid identifications are outlined in Kyle et al. (2017).[8] Reconstituted lipids were analyzed using a Waters Aquity UPLC H class system interfaced with a Velos-ETD Orbitrap mass spectrometer is used for LC-ESI-MS/MS analyses. A Waters CSH column (3.0 mm x 150 mm x 1.7 µm particle size) is used to separate lipid molecular species over a 34 min gradient (mobile phase A: ACN/H_2O (40:60) containing 10 mM ammonium acetate; mobile phase B: ACN/IPA (10:90) containing 10 mM ammonium acetate) at a flow rate of 250 µl/min. Eluting lipids are introduced to the MS via electrospray ionization in both positive and negative modes, and lipids are fragmented using HCD (higher-energy collision dissociation) and CID (collision-induced dissociation) to obtain high coverage of the lipidome. Lipid identifications were made using in-house developed identification software LIQUID where the tandem mass spectra were examined for diagnostic ion fragments along with associated hydrocarbon chain fragment information.[8] In addition, the isotopic profile, extracted ion chromatogram, and mass measurement error of precursor ions were examined for each lipid species. To facilitate quantification of lipids, a reference database for lipids identified from the MS/MS data was created, containing lipid name, observed *m/z*, and retention time. Lipid features from each analysis were then aligned to the reference database based on their *m/z* and retention time using MZmine 2.[9] Aligned features were manually verified and peak apex intensity values were exported for subsequent statistical analysis.

Table S1

Table S1. Significant plasma metabolites in subject 1 (FDR < 0.05).

Ionization mode	Mode	Retention time (min)	Metabolite	PATHWAY	KEGG	HMDB	Species	Z-score	FDR	Median Intensity	Mass error (ppm)
ESI (-) MS	nHILIC	10.3	Malic acid	TCA Cycle	C00149	HMDB00156	[M-H]-	3.9	2.73E-03	2.8E+08	-0.6
ESI (-) MS	nHILIC	11	Citric acid	TCA Cycle	C00158	HMDB00094	[M-H]-	3.0	2.12E-02	2.5E+09	-0.7
ESI (-) MS	nHILIC	7.5	L-Valine	branched chain AA Metabolism	C00183	HMDB00883	[M-H]-	-3.1	1.87E-02	5.5E+08	-0.1
ESI (+) MS	pHILIC	6.5	L-Isoleucine|L-Leucine	branched chain AA Metabolism	C00407<br>C00123	HMDB00172<br>HMDB00687	[M+H]+	-2.8	3.35E-02	1.7E+09	-1.8
ESI (+) MS	pHILIC	6.8	Betaine	Glycine, Serine, Threonine Metabolism	C00719	HMDB00043	[M+H]+	-2.9	2.56E-02	4.3E+07	-0.8
ESI (-) MS	nHILIC	8.9	L-Threonine	Glycine, Serine, Threonine Metabolism	C00188	HMDB00167	[M-H]-	-3.5	1.02E-02	5.4E+08	-0.5
ESI (-) MS	nHILIC	7.7	N-Acetylserine	Glycine, Serine, Threonine Metabolism		HMDB02931	[M-H]-	2.8	3.35E-02	7.4E+07	0.6
ESI (+) MS	pHILIC	6.8	L-Tryptophan	Tryptophan Metabolism	C00078	HMDB00929	[M+H]+	-2.9	2.90E-02	7.1E+08	-1.6
ESI (-) MS	nHILIC	3.4	Indoxyl sulfate	Tryptophan Metabolism		HMDB00682	[M-H]-	-2.9	2.83E-02	1.9E+09	-1.3
ESI (+) MS	pHILIC	16	Ornithine	Arginine and Proline Metabolism	C00077	HMDB03374	[M+H]+	-5.4	9.22E-06	4.0E+08	-2.2
ESI (+) MS	pHILIC	8.3	Butyrylcarnitine	Fatty Acid Metabolism	C02862	HMDB02013	[M+H]+	-3.2	1.69E-02	8.3E+07	-3.2
ESI (-) MS	nRPLC	7	Decatrienoic acid	Fatty Acid Metabolism			[M-H]-	2.7	3.63E-02	4.7E+07	0.1
ESI (-) MS	nRPLC	10.3	Hydroxypalmitic acid	Fatty Acid Metabolism			[M-H]-	2.7	4.09E-02	3.3E+07	0.2
ESI (-) MS	nRPLC	10.2	Hydroxyoleic acid	Fatty Acid Metabolism			[M-H]-	3.0	2.37E-02	3.7E+07	0.3
ESI (+) MS	pHILIC	10.4	Glycerophosphocholine	Phospholipid Metabolism	C00670	HMDB00086	[M+H]+	-3.3	1.33E-02	4.4E+08	-2.4
ESI (+) MS	pRPLC	10.2	LysoPC(P-16:0)	Phospholipid Metabolism	C04230	HMDB10407	[M+H]+	-2.9	2.79E-02	4.3E+07	-2.3
ESI (+) MS	pRPLC	9.9	LysoPC(16:0)	Phospholipid Metabolism	C04230	HMDB10382	[M+H]+	-3.5	8.97E-03	4.0E+09	-1.1
ESI (+) MS	pRPLC	10.3	LysoPC(17:0)	Phospholipid Metabolism	C04230	HMDB12108	[M+H]+	-2.8	3.52E-02	7.1E+07	-0.8
ESI (+) MS	pRPLC	10.6	LysoPC(18:0)	Phospholipid Metabolism			[M+H]+	-3.7	5.68E-03	7.7E+08	0.8
ESI (+) MS	pHILIC	5.9	LysoPC(20:3)	Phospholipid Metabolism	C04230	HMDB10393	[M+H]+	-3.0	2.45E-02	4.8E+07	-1.3
ESI (+) MS	pRPLC	9.5	LysoPC(20:5)	Phospholipid Metabolism	C04230	HMDB10397	[M+H]+	-2.6	4.63E-02	4.0E+07	0.9
ESI (+) MS	pHILIC	4.7	PC(P-36:3)	Phospholipid Metabolism			[M+H]+	-2.8	3.54E-02	7.0E+07	-3.6
ESI (+) MS	pHILIC	4.7	PC(36:4)	Phospholipid Metabolism			[M+H]+	-2.7	4.26E-02	8.5E+08	-4.3
ESI (+) MS	pHILIC	4.6	PC(40:5)	Phospholipid Metabolism	C00157		[M+H]+	-2.8	3.12E-02	4.1E+07	-4.9
ESI (+) MS	pRPLC	9.4	Sphingosine 1-phosphate	Sphingolipid Metabolism	C06124	HMDB00277	[M+H]+	-2.7	3.81E-02	5.8E+07	-1.7
ESI (-) MS	nRPLC	9.3	Androsterone sulfate(1)	Androgenic Steroids		HMDB02759	[M-H]-	-5.3	1.42E-05	1.4E+08	0.6
ESI (-) MS	nRPLC	9.6	Androsterone sulfate(2)	Androgenic Steroids		HMDB02759	[M-H]-	-5.4	9.22E-06	3.8E+08	0.6
ESI (-) MS	nRPLC	9.9	Androsterone sulfate(3)	Androgenic Steroids		HMDB02759	[M-H]-	-4.5	3.65E-04	3.4E+07	0.7
ESI (-) MS	nRPLC	9	Testosterone sulfate	Androgenic Steroids		HMDB02833	[M-H]-	-3.2	1.65E-02	1.3E+09	1.9
ESI (+) MS	pRPLC	8.6	Piperine	Food Component/Plant	C03882	HMDB29377	[M+H]+	-2.7	3.95E-02	1.3E+08	-1.6
ESI (-) MS	nHILIC	11.3	C10H14N2O7				[M-H]-	3.0	2.08E-02	6.8E+07	-0.5
ESI (-) MS	nRPLC	9	C26H26O7				[M-H]-	-3.2	1.56E-02	3.4E+07	3.0
ESI (-) MS	nHILIC	10.8	C6H10O8				[M-H]-	-3.2	1.61E-02	5.2E+07	-0.1
ESI (-) MS	nHILIC	10.5	C6H9NO6				[M-H]-	-2.6	4.98E-02	7.8E+08	-1.0
ESI (-) MS	nHILIC	10.5	C7H14N2O4				[M-H]-	2.6	4.88E-02	7.9E+07	-0.7
ESI (+) MS	pHILIC	10.3	C8H16N2O4				[M+H]+	3.1	1.87E-02	4.8E+07	-1.9
ESI (-) MS	nHILIC	3.6	C9H14N2O6				[M-H]-	3.4	1.10E-02	1.6E+08	-0.9
ESI (-) MS	nHILIC	10.5	C9H14N2O7				[M-H]-	2.9	2.56E-02	1.2E+08	-1.7
ESI (-) MS	nHILIC	11.3	C9H16N2O6				[M-H]-	3.4	1.02E-02	7.9E+07	0.0
ESI (-) MS	nHILIC	11.2	Thiosulfic acid			HMDB60293	[M-H]-	2.7	4.33E-02	1.3E+08	-1.0
ESI (+) MS	pHILIC	7.4	C4H9N				[M+H]+	-2.8	3.38E-02	4.1E+08	-0.3

Identifications are confirmed by matching MS/MS spectra to spectral libraries or references when available. Elemental composition was determined using isotopic distribution and accurate mass. Androsterone sulfate elutes in multiple peaks - labeled 1, 2 and 3.

Table S2

Table S2. Statistically significant lipids identified in subject 1 ($p<0.05$)

Lipid Common Name	Fold Change	Zscore	Pvalue
carnitine(12:1)	2.67	2.51	1.21E-02
carnitine(14:1)	3.20	2.73	6.33E-03
carnitine(16:0)	1.46	2.47	1.33E-02
Palmitoyl-EA (endocannabinoid)	1.20	2.30	2.13E-02
CE(18:1)	0.63	2.05	4.00E-02
CE(18:2)	1.01	3.26	1.12E-03
Cer(d18:0/24:0)	-0.91	-2.10	3.56E-02
Cer(d18:0/24:1)	-1.76	-2.71	6.75E-03
Cer(d18:1/23:0)	-1.01	-2.04	4.16E-02
Cer(d18:1/26:0)	-1.29	-2.34	1.94E-02
SM(d16:1/22:0);SM(d18:1/20:0)	0.85	2.24	2.53E-02
SM(d18:1/16:0)	0.82	2.55	1.08E-02
SM(d18:1/18:0);SM(d16:1/20:0)	1.06	2.52	1.19E-02
SM(d18:1/24:1)	1.23	2.83	4.70E-03
SM(d18:2/18:0)	1.01	2.06	3.93E-02
SM(d18:2/20:0)	1.47	3.18	1.46E-03
SM(d18:2/24:1)	1.54	3.48	5.03E-04
PC(20:2/0:0)	1.41	1.98	4.72E-02
PC(0:0/20:4)	1.31	2.08	3.78E-02
PC(16:0/16:0)	1.16	2.91	3.60E-03
PC(16:0/18:1)	0.97	3.34	8.24E-04
PC(16:1/18:1);PC(16:0/18:2)	0.94	3.24	1.20E-03
PC(18:0/18:2)	0.93	3.36	7.83E-04
PC(18:1/18:2)	0.96	2.49	1.28E-02
PC(16:0/20:4)	0.75	2.31	2.08E-02
PC(18:0/20:4);PC(18:1/20:3)	0.97	2.71	6.80E-03
PC(16:0/22:6)	0.98	2.01	4.42E-02
PC(20:0/20:4)	1.10	2.16	3.09E-02
PC(20:2/20:4);PC(18:1/22:5)	1.40	3.12	1.80E-03
PC(O-34:1);PC(P-34:0)	1.28	2.92	3.50E-03
PC(O-18:1/18:2);PC(P-18:0/18:2)	1.47	2.24	2.50E-02
PE(16:0/18:0)	0.89	2.06	3.96E-02
PE(18:1/20:4)_A;PE(16:0/22:5)	1.32	2.32	2.04E-02
PE(O-18:0/18:1);PE(P-20:0/16:0)	1.03	2.53	1.14E-02
PE(O-18:0/20:4)	-1.04	-2.09	3.62E-02
PE(P-16:0/20:3)	-1.42	-2.23	2.56E-02
PI(16:0/18:1);PI(16:1/18:0)	-1.43	-2.25	2.47E-02
PI(16:0/20:3);PI(18:1/18:2)	-1.60	-2.36	1.81E-02
PI(16:0/20:4)	-1.61	-2.45	1.42E-02
PI(18:1/20:4)	-2.10	-2.17	2.99E-02
TG(14:0/16:0/18:1);TG(16:0/16:0/16:1)	-2.00	-2.07	3.87E-02
TG(48:1)	-2.00	-2.07	3.87E-02
TG(16:1/18:1/18:2);TG(16:0/18:2/18:2);TG(16:0/18:1/18:3)	0.99	2.11	3.49E-02
TG(56:4)	1.75	2.85	4.31E-03
TG(18:1/18:2/20:4);TG(16:0/18:2/22:5)	1.30	2.36	1.82E-02
TG(18:2/18:2/20:4);TG(18:1/18:3/20:4);TG(18:1/18:2/20:5)	1.54	2.10	3.59E-02
TG(18:0/18:1/22:0);TG(16:0/18:1/24:0)	-2.07	-1.96	4.99E-02

Lipid common name annotation ZZ(X1:Y1/X2:Y2) where ZZ = lipid class; X1 = number of carbons in chain 1; Y1 = number of double bonds in chain 1; X2 = number of carbons in chain 2; Y1 = number of double bonds in chain 2. Identifications with ZZ(X:Y) denotes the total number of carbons (X) and double bonds (Y) in the chains. CE = cholesterol ester; Cer = ceramide; SM = sphingomyelin; PC = glycerophosphocholine; PCO and PCP = alkyl and alkenyl glycerophosphocholine, respectively; PE = glycerophosphoethanolamine; PEO and PEP = alkyl and alkenyl glycerophosphoethanolamine, respectively; PI = glycerophosphoinositol; TG = triacylglycerol. Zscore coloring scales from +3 (red) to -3 (blue).

Table S3

Table S3. Statistically significant lipids identified in female flies (p<0.05)

Lipid Common Name	*ATPsynδ* Kd (#7018)	*ATPsynδ* Kd (#7019)	*ATPsynδ* Kd (#7018)	*ATPsynδ* Kd (#7019)
	log2 fold change		p-value	
carnitine(12:0)	0.885	0.839	0.0017	0.0015
carnitine(14:1)	0.705	1.065	0.0155	0.0009
carnitine(16:0)	-0.639	-0.431	0.0369	0.1179
Cer(d14:0/20:0)	0.453	0.373	0.0202	0.0340
Cer(d14:1/18:0)	0.039	0.407	0.8978	0.0036
Cer(d14:1/20:0)	-0.059	0.426	0.8508	0.0092
Cer(d16:1/22:1)	0.210	0.193	0.0209	0.0213
Cer(d14:2/22:0)	0.244	0.144	0.0210	0.1240
PE_Cer(d14:1/22:0)	0.008	0.123	0.9785	0.0307
PE_Cer(d14:2/24:0)	0.140	0.082	0.0439	0.1959
PE_Cer(d16:1/24:0)	-0.260	-0.255	0.0610	0.0474
CL(62:3)	1.406	1.226	0.0023	0.0033
CL(62:4)	1.466	1.333	0.0110	0.0119
CL(64:4)	0.366	0.642	0.0847	0.0041
CL(64:5)	0.980	0.629	0.0380	0.1407
CL(66:4)	0.486	1.075	0.3417	0.0201
CL(70:8)	-0.295	-0.559	0.3376	0.0391
CL(72:8)	-0.237	-0.316	0.1165	0.0279
CL(72:9)	-0.415	-0.790	0.0167	0.0002
PC(20:0/0:0)	-0.199	-0.418	0.3310	0.0240
PC(12:0/12:0)	1.052	0.523	0.0000	0.0011
PC(12:0/13:0)	0.958	0.493	0.0092	0.1135
PC(12:0/14:0)	0.838	0.687	0.0056	0.0107
PC(12:0/14:1)	0.913	0.624	0.0014	0.0081
PC(14:0/14:0)	0.534	0.274	0.0001	0.0035
PC(12:0/16:1)	0.829	0.567	0.0006	0.0041
PC(14:0/15:0)_B;PC(13:0/16:0)	0.157	-0.590	0.3554	0.0011
PC(14:0/16:1)	0.405	0.295	0.0023	0.0091
PC(14:1/16:0)	0.368	0.420	0.0306	0.0108
PC(14:1/16:1)	0.729	0.363	0.0029	0.0630
PC(30:3)	0.811	0.373	0.0012	0.0484
PC(15:0/16:0);PC(14:0/17:0)	-0.043	-0.588	0.9505	0.0073
PC(15:0/16:1)	0.066	-0.395	0.8160	0.0134
PC(16:0/16:1)	0.159	0.217	0.1225	0.0268
PC(14:0/18:2);PC(16:1/16:1)	0.308	0.080	0.0021	0.3280
PC(14:1/18:2)	1.136	0.537	0.0009	0.0356
PC(16:0/17:0)	-0.092	-0.317	0.6731	0.0347
PC(15:0/18:2)	-0.101	-0.588	0.6001	0.0009
PC(16:1/17:1)	-0.036	0.328	0.8492	0.0023
PC(16:0/18:1)	0.000	0.151	1.0000	0.0043
PC(16:1/18:1)	0.096	0.185	0.1263	0.0048
PC(17:0/18:1)	-0.216	-0.443	0.2168	0.0099
PC(17:0/18:2)	-0.254	-0.578	0.1168	0.0015
PC(18:0/18:1)	0.185	0.297	0.1623	0.0198
PC(18:0/18:2)	0.089	-0.180	0.3820	0.0410
PC(18:1/18:1)	0.041	0.217	0.8002	0.0205
PC(18:2/18:3)	0.030	-0.506	0.9445	0.0016
PC(18:3/18:3)	-0.177	-0.578	0.6176	0.0275
PC(17:1/20:2);PC(18:2/19:1)	-0.410	-0.309	0.0053	0.0156
PC(18:1/20:0)	-0.354	-0.134	0.0490	0.4713
PC(18:2/20:0)	-0.542	-0.475	0.0092	0.0121
PC(18:1/20:2);PC(18:2/20:1)	-0.266	-0.252	0.0156	0.0138
PC(18:2/20:2)	-0.355	-0.480	0.0362	0.0052
PC(18:1/21:0)	-0.791	-0.516	0.0049	0.0294
PC(O-18:1/18:2)	0.588	0.015	0.0193	0.9938

Table S3. Continued

Lipid Common Name	*ATPsynδ* Kd (#7018)	*ATPsynδ* Kd (#7019)	*ATPsynδ* Kd (#7018)	*ATPsynδ* Kd (#7019)
	log2 fold change		p-value	
PE(12:0/0:0)	0.525	0.797	0.1959	0.0351
PE(14:0/0:0)	0.354	0.672	0.2988	0.0296
PE(12:0/12:0)	0.503	0.532	0.0484	0.0265
PE(12:0/14:0)	0.413	0.699	0.1019	0.0067
PE(14:0/14:1);PE(12:0/16:1)	0.915	0.766	0.0052	0.0090
PE(14:1/16:1);PE(12:0/18:2)	0.722	0.405	0.0045	0.0519
PE(15:0/16:0);PE(14:0/17:0)	-0.270	-0.804	0.2740	0.0021
PE(15:0/16:1)	0.010	-0.620	0.9986	0.0275
PE(15:1/16:1)	0.456	0.194	0.0132	0.2307
PE(16:0/16:0)	-0.520	-0.644	0.0919	0.0282
PE(16:0/17:0)	-0.319	-0.539	0.0734	0.0040
PE(15:0/18:2)	-0.455	-0.345	0.0133	0.0341
PE(16:0/18:1)	-0.055	0.230	0.4936	0.0023
PE(17:0/18:1)	-0.372	-0.185	0.0456	0.2926
PE(17:1/18:2)	-0.384	-0.286	0.0236	0.0590
PE(18:3/18:3)	-0.167	-0.698	0.7492	0.0315
PE(18:1/19:0)	-0.167	-0.278	0.2670	0.0419
PE(18:1/19:1);PE(18:2/19:0)	0.261	0.014	0.0266	0.9773
PE(18:2/19:1)	-0.099	-0.445	0.7284	0.0175
PE(18:2/20:0)	-0.360	-0.361	0.0324	0.0220
PE(18:2/20:2)	-0.350	-0.547	0.1313	0.0157
PE(18:1/21:0)	-0.829	-0.385	0.0004	0.0238
PE(18:2/21:0)	-1.077	-0.695	0.0001	0.0013
PE(18:1/22:0)	-0.822	-0.269	0.0005	0.1076
PE(18:2/22:0)	-0.913	-0.759	0.0001	0.0001
PE(24:0/18:1)	-0.701	-0.157	0.0036	0.4725
PE(O-16:0/16:1)	0.091	0.299	0.4984	0.0095
PE(O-18:0/16:1)	0.338	0.233	0.0328	0.1005
PE(O-20:0/18:1)	0.580	0.451	0.0016	0.0045
PE(P-16:0/18:2)	-0.352	-0.100	0.0271	0.5610
PE(P-18:0/16:1)	0.122	0.362	0.3255	0.0036
PE(P-18:0/18:1)	0.256	0.247	0.0021	0.0016
PE(P-20:0/18:1)	0.672	0.605	0.0020	0.0023
PE(P-20:0/18:3)	-0.107	-0.302	0.5250	0.0259
PG(14:0/14:0)	1.176	0.421	0.0032	0.1901
PG(14:0/14:1);PG(12:0/16:1)	0.743	1.108	0.0743	0.0082
PG(14:0/16:0)	0.638	0.293	0.0282	0.2760
PG(14:0/16:1)	0.621	0.443	0.0221	0.0659
PG(16:1/17:1);PG(15:0/18:2)	0.418	0.609	0.0784	0.0100
PG(16:0/18:1)	-0.026	0.370	0.9612	0.0126
PG(16:0/19:1)			0.0040	0.0017
PG(18:1/18:1)	-0.121	0.451	0.7017	0.0320
PI(12:0/14:0)	1.032	0.315	0.0018	0.2227
PI(14:0/14:0);PI(12:0/16:0)	0.605	0.012	0.0338	0.9972
PI(12:0/16:1)	0.885	0.511	0.0020	0.0256
PI(14:0/16:0)	0.639	0.286	0.0273	0.2860
PI(14:0/16:1)	0.736	0.321	0.0002	0.0181
PI(14:1/16:1)	1.084	0.636	0.0073	0.0615
PI(16:1/16:1)	0.479	0.166	0.0130	0.3461
PI(16:1/17:1)	0.369	0.002	0.0476	0.9998
PI(16:0/18:2)	-0.030	-0.172	0.8442	0.0266
PI(16:0/18:3)	-0.179	-0.306	0.1839	0.0183
PI(18:1/18:1)	0.150	0.337	0.2833	0.0115
PI(18:2/18:2)	0.218	-0.407	0.1037	0.0039
PI(18:2/18:3)	0.117	-0.565	0.6676	0.0062
PI(18:3/18:3)	-0.061	-0.626	0.9433	0.0222
PI(18:2/20:2)	-0.852	-0.913	0.0045	0.0018
PS(14:0/16:1);PS(12:0/18:1)	0.465	0.427	0.0072	0.0073
PS(14:0/18:2);PS(16:1/16:1)	0.308	0.249	0.0300	0.0523

Table S3. Continued

Lipid Common Name	*ATPsynδ* Kd (#7018)	*ATPsynδ* Kd (#7019)	*ATPsynδ* Kd (#7018)	*ATPsynδ* Kd (#7019)
	log2 fold change		p-value	
PS(18:3/18:3)	-0.242	-0.440	0.2456	0.0233
DG(12:0/12:0/0:0)	0.604	0.173	0.0280	0.5600
DG(15:0/18:1/0:0);DG(16:1/17:0/0:0)	-0.542	-0.776	0.2021	0.0474
DG(16:0/18:1/0:0)	-0.760	-0.649	0.0180	0.0261
DG(16:0/18:2)	-0.777	-0.725	0.0074	0.0070
DG(16:1/18:1/0:0)	-0.711	-0.422	0.0022	0.0241
DG(16:1/18:2/0:0)	-0.606	-0.823	0.0803	0.0150
DG(17:0/18:1/0:0)	-0.673	-0.692	0.0226	0.0132
DG(18:0/18:1/0:0)	-0.430	-0.306	0.0035	0.0147
DG(18:1/0:0/18:1)	-0.670	-0.332	0.0004	0.0172
DG(18:1/18:2/0:0)	-0.362	-0.546	0.0055	0.0002
DG(18:2/0:0/18:2);DG(18:1/18:3/0:0)	-0.119	-0.568	0.2911	0.0001
TG(34:0)	0.919	1.154	0.0045	0.0007
TG(36:0)	0.789	0.650	0.0005	0.0011
TG(37:0)	0.563	0.527	0.0178	0.0166
TG(38:1)_A	0.574	0.636	0.0047	0.0016
TG(38:1)_B	0.601	0.749	0.0028	0.0004
TG(39:1)	0.188	0.367	0.3051	0.0275
TG(40:1)	0.368	0.426	0.0382	0.0130
TG(40:2)	0.261	0.658	0.2482	0.0043
TG(42:2)	0.206	0.386	0.1345	0.0062
TG(44:3)	0.055	0.465	0.8342	0.0027
TG(49:3)	-0.393	-0.161	0.0008	0.0583

Lipid common name annotation ZZ(X1:Y1/X2:Y2) where ZZ = lipid class; X1 = number of carbons in chain 1; Y1 = number of double bonds in chain 1; X2 = number of carbons in chain 2; Y1 = number of double bonds in chain 2. Identifications with ZZ(X:Y) denotes the total number of carbons (X) and double bonds (Y) in the chains. CE = cholesterol ester; Cer = ceramide; PE-Cer = phosphoethanolamine ceramide; CL = cardiolipin; PC = glycerophosphocholine; PCO and PCP = alkyl and alkenyl glycerophosphocholine, respectively; PE = glycerophosphoethanolamine; PEO and PEP = alkyl and alkenyl glycerophosphoethanolamine, respectively; PG = glycerophosphoglycerol; PI = glycerophosphoinositol; PS = glycerophosphoserine; DG = diacylglycerol; TG = triacylglycerol. Common name with '_A' and '_B' indicates the lipids are structural isomers of each other. Log2 fold change coloring scales from +1 (red) to -1 (blue). Red font p-values indicates p-value less than 0.05.

Control and 2 mutant female fly lines were analyzed. The control is a *tub-Gal80[ts] (*Bloomington # *7019)/UAS-ATPsynδ RNAi* and the mutants are *tub-Gal80[ts] (*Bloomington # *7019)/UAS-ATPsynδ RNAi; da-Gal4* (ubiquitous driver)/+ and *tub-Gal80[ts] (*Bloomington # *7018)/UAS-ATPsynδ RNAi; da-Gal4* (ubiquitous driver)/+. For conditional knock-down of *ATPsynδ RNAi* using *tubP-GAL80*^{ts}*,* flies were reared at room temperature (21°C) during development, and the adult flies were kept for three days at 28°C before metabolomics analysis. Prior to extracting the lipids as in table s2, 0.15 mm zirconia oxide beads and 0.3 mL of methanol were added to tubes containing female and male flies (n=15 and 3 replicates each) and placed in -80 ºC pre-chilled Eppendorf Safe-Lock tube holder. Samples were homogenized using a Bullet Blender (BB50-DX) for 3 mins at speed 10. The homogenized samples were centrifuged at 8000 *x g* for 10 min at 4 ºC and the lysate was transferred into to 2.0 mL Sorenson low-binding microcentrifuge tubes. An addition 100 µl of methanol, 800 µl chloroform, and 300 µl of water was then added to the lysate and processed as the plasma samples outlined above. Lipids were analyzed as outlined above with the plasma samples. The algorithm RMD-PAV and Pearson correlation were used to identify any outlier biological samples, with none removed.[10] Molecules with inadequate data for either qualitative or quantitative statistical tests were also removed from the datasets prior to normalization via global median centering.[11] ANOVA with a Dunnett test correction and a Bonferroni-corrected g-test was used to compare each mutant to the control.

Supplemental References

1. Smet, J., Seneca, S., de Paepe, B., Meulemans, A., Verhelst, H., Leroy, J., De Meirleir, L., Lissens, W., Van Coster, R. (2009). Subcomplexes of mitochondrial complex V reveal mutations in mitochondrial DNA. *Electrophoresis* 30, 3565-3572.

2. Dietzl, G., Chen, D., Schnorrer, F., Su, K.-C., Barinova, Y., Fellner, M., Gasser, B., Kinsey, K., Oppel, S., Scheiblauer, S., et al. (2007). A genome-wide transgenic RNAi library for conditional gene inactivation in Drosophila. *Nature* 448, 151–156.

3. Bischof, J., Maeda, R.K., Hediger, M., Karch, F., and Basler, K. (2007). An optimized transgenesis system for Drosophila using germ-line-specific phiC31 integrases. *Proc. Natl. Acad. Sci. U. S. A.* 104, 3312–3317.

4. Jaiswal, M., Haelterman, N.A., Sandoval, H., Xiong, B., Donti, T., Kalsotra, A., Yamamoto, S., Cooper, T.A., Graham, B.H., and Bellen, H.J. (2015). Impaired mitochondrial energy production causes light-induced photoreceptor degeneration independent of oxidative stress. *PLoS Biol.* 13 (7): e1002197.

5. Chennamsetty, I., Coronado, M., Contrepois, K., Keller, M.P., Carcamo-Orive, I., Sandin, J., Fajardo, G., Whittle, A.J., Fathzadeh, M., Snyder, M., et al. (2016). Nat1 Deficiency Is Associated with Mitochondrial Dysfunction and Exercise Intolerance in Mice. *Cell Rep* 17, 527–540.

6. Contrepois, K., Jiang, L., and Snyder, M. (2015). Optimized Analytical Procedures for the Untargeted Metabolomic Profiling of Human Urine and Plasma by Combining Hydrophilic Interaction (HILIC) and Reverse-Phase Liquid Chromatography (RPLC)–Mass Spectrometry. *Mol. Cell. Proteomics* 14, 1684–1695.

7. Nakayasu, E.S., Nicora, C.D., Sims, A.C., Burnum-Johnson, K.E., Kim, Y.-M., Kyle, J.E., Matzke, M.M., Shukla, A.K., Chu, R.K., Schepmoes, A.A., et al. (2016). MPLEx: a Robust and Universal Protocol for Single-Sample Integrative Proteomic, Metabolomic, and Lipidomic Analyses. *mSystems* 1, e00043-16.

8. Kyle, J.E., Crowell, K.L., Casey, C.P., Fujimoto, G.M., Kim, S., Dautel, S.E., Smith, R.D., Payne, S.H., and Metz, T.O. (2017). LIQUID: An-open source software for identifying lipids in LC-MS/MS-based lipidomics data. *Bioinformatics* 33, 1744–1746.

9. Pluskal, T., Castillo, S., Villar-Briones, A., and Orešič, M. (2010) MZmine 2: Modular framework for processing, visualizing, and analyzing mass spectrometry- based molecular profile data. *BMC Bioinformatics* 11:395.

10. Matzke, M.M., Waters, K.M., Metz, T.O., Jacobs, J.M., Sims, A.C., Baric, R.S., Pounds, J.G., and Webb-Robertson, B.-J.M. (2011). Improved quality control processing of peptide-centric LC-MS proteomics data. *Bioinformatics* 27 (20) 2866–2872.

11. Webb-Robertson, B.-J.M., Mccue, L.A., Waters, K.M., Matzke, M.M., Jacobs, J.M., Metz, T.O., Varnum, S.M., and Pounds, J.G. (2010) Combined Statistical Analyses of Peptide Intensities and Peptide Occurrences Improves Identification of Significant Peptides from MS-Based Proteomics Data. *J Proteome Res.* 9(11):5748-56.
